# Germline-encoded amino acid-binding motifs drive immunodominant public antibody responses

**DOI:** 10.1126/science.adc9498

**Published:** 2023-04-07

**Authors:** Ellen L. Shrock, Richard T. Timms, Tomasz Kula, Elijah L. Mena, Anthony P. West, Rui Guo, I-Hsiu Lee, Alexander A. Cohen, Lindsay G. A. McKay, Caihong Bi, Keerti, Yumei Leng, Eric Fujimura, Felix Horns, Mamie Li, Duane R. Wesemann, Anthony Griffiths, Benjamin E. Gewurz, Pamela J. Bjorkman, Stephen J. Elledge

**Affiliations:** 1 Department of Genetics, Harvard Medical School, Boston, MA 02115, USA; 2 Division of Genetics, Department of Medicine, Howard Hughes Medical Institute, Brigham and Women’s Hospital, Boston, MA 02115, USA; 3 Program in Biological and Biomedical Sciences, Harvard University, Boston, MA 02115, USA; 4 Cambridge Institute of Therapeutic Immunology and Infectious Disease, Jeffrey Cheah Biomedical Centre, Cambridge Biomedical Campus, University of Cambridge, Cambridge, UK; 5 Present address: Society of Fellows, Harvard University, Cambridge, MA 02138, USA; 6 Division of Biology and Biological Engineering, California Institute of Technology, Pasadena, CA 91125, USA; 7 Division of Infectious Disease, Department of Medicine, Brigham and Women’s Hospital, Harvard Medical School, Boston, MA 02115, USA; 8 Department of Microbiology, Harvard Medical School, Boston, MA 02115, USA; 9 Broad Institute of Harvard and MIT, Cambridge, MA, 02142, USA; 10 Center for Systems Biology, Department of Radiology, Massachusetts General Hospital and Harvard Medical School, Boston, MA 02114, USA; 11 National Emerging Infectious Diseases Laboratories, Boston University School of Medicine, Boston University, Boston, MA 02118, USA; 12 Division of Allergy and Immunology, Division of Genetics, Department of Medicine, Brigham and Women’s Hospital, Harvard Medical School, Boston, MA 02115, USA; 13 Massachusetts Consortium on Pathogen Readiness, Boston, MA 02115, USA; 14 Department of Bioengineering, Department of Applied Physics, Chan Zuckerberg Biohub and Stanford University, Stanford, CA 94305, USA; 15 Ragon Institute of MGH, MIT, and Harvard, Cambridge, MA, 02139 USA; 16 Graduate Program in Virology, Division of Medical Sciences, Harvard Medical School, Boston, MA 02115, USA

## Abstract

Despite the vast diversity of the antibody repertoire, infected individuals often mount antibody responses to precisely the same epitopes within antigens. The immunological mechanisms underpinning this phenomenon remain unknown. Here, by mapping 376 immunodominant “public epitopes” at high resolution and characterizing several of their cognate antibodies, we conclude that germline-encoded sequences in antibodies drive recurrent recognition. Systematic analysis of antibody–antigen structures uncovered 18 human and 21 partially overlapping mouse germline-encoded amino acid-binding (GRAB) motifs within heavy and light V gene segments which, in case studies, are critical for public epitope recognition. GRAB motifs represent a fundamental component of the immune system’s architecture that ensures antibody recognition of pathogens and promotes species-specific reproducible responses that can exert selective pressure on pathogens.

## One-sentence summary

Antibody genes harbor GRAB motifs that bind specific amino acids and drive reproducible responses to certain immunodominant epitopes across individuals.

## Introduction

The adaptive immune system relies on an extremely diverse antibody repertoire to mount a response to any pathogen encountered. Antibody diversity is generated by a DNA recombination mechanism occurring in the heavy and light chain genes in which modular VDJ (for heavy) and VJ (for light) gene segments are combinatorially assembled to generate a vast repertoire of variable domain sequences. Immunoglobulin G (IgG) is composed of two heavy and two light chains arranged as a heterodimer with two identical antigen-binding sites, each formed by paired heavy and light chain variable domains. A given antibody has either a kappa or a lambda light chain, which have no known functional difference. Antigen recognition is accomplished primarily by complementarity determining regions (CDRs), which are hypervariable loops within the heavy and light chain variable domains (three in each domain). The heavy and light chain CDR1s and CDR2s are encoded by the V gene segments, whereas the CDR3s span the junctions of the recombined VDJ or VJ gene segments and are thus highly diverse and generally thought to play a dominant role in determining specificity (3.1).

The complexity of the antibody repertoire enables the generation of antibodies to virtually any antigen, yet isolated examples of recurrent responses in different individuals to particular epitopes have been reported (3.2–3.13). Given the challenge of mapping antibody epitopes at high resolution, it has been unclear how common recurrent antibody responses are and how widely they are shared across human populations. Recently, we developed VirScan, a phage display platform programmed to display peptides spanning the human virome, which enabled the high-throughput identification of antiviral antibody epitopes (3.14–3.18). We used VirScan to profile hundreds of human serum samples (3.14) and found that although many viral peptides recognized by an individual were relatively specific to that person, many other viral peptides—which we termed “public epitopes”—were recognized by a substantial percentage (≤98%) of individuals seropositive for the given virus (3.14). Public epitopes were also observed in VirScan studies of antibody responses to allergens and symbiotic microbiota (3.19–3.21). These findings raised a fundamental question: what mechanisms drive recurrent responses to public epitopes?

## Results

### Public epitopes are a general feature of the human antibody response

**Fig. 3.1.**
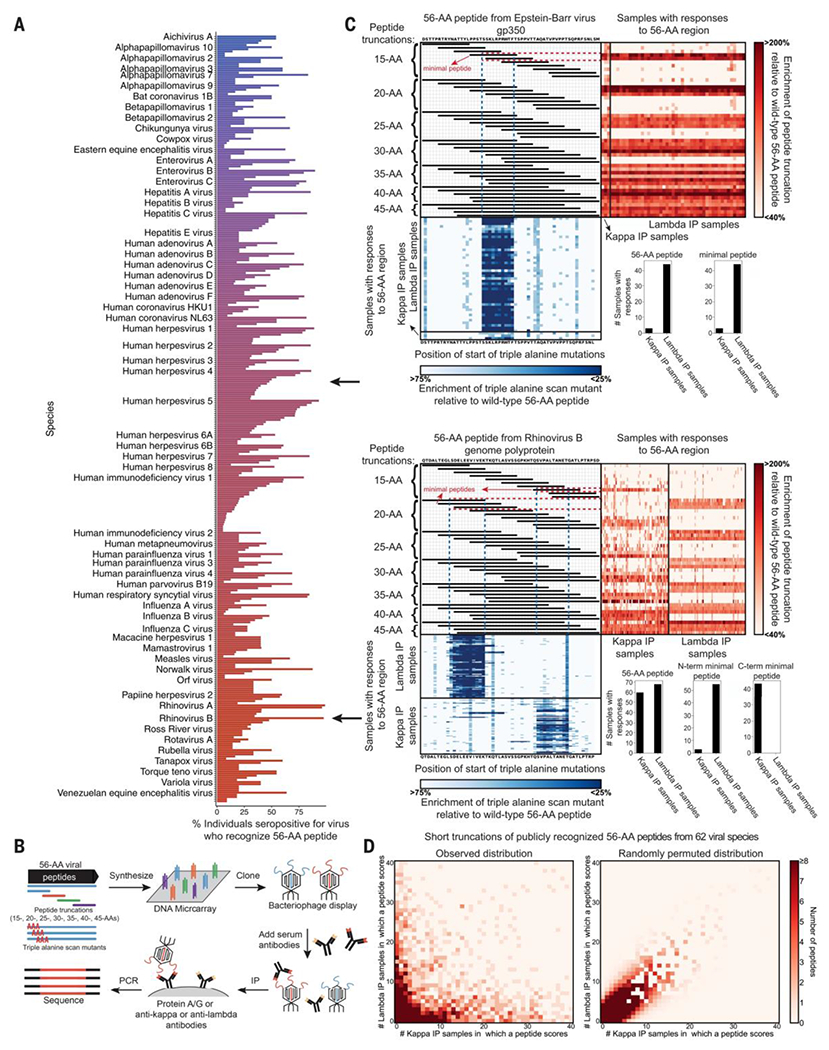
Recurrent antibody responses to public epitopes are a general feature of humoral immunity and antibodies that recognize a given public epitope have biased light chain isotype usage. (**A**) Percentage of individuals seropositive for a given virus who exhibit antibody responses to each publicly recognized 56-AA peptide. For each viral species, publicly recognized 56-AApeptides are arranged in descending order by their value on the x axis. Arrows indicate the peptides detailed in (C). (**B**) Schematic representation of the VirScan assay using the public epitope truncation and alanine scan library (IP, immunoprecipitation). (**C**) Antibody responses to public epitopes from EBV envelope glycoprotein gp350(top) and rhinovirus B genome polyprotein (bottom) as characterized by VirScan using the kappa and lambda isotype-specific IP protocol. The subset of serum samples exhibiting antibody responses to at least one peptide from the 56-AA region are shown; data are the mean of two technical replicates. AAs are identified by their standard one-letter abbreviations. (**D**) Number of kappa and lambda IP samples that recognize short truncations (15, 20, 25, and 30 AAs in length) of publicly recognized 56-AA peptides from 62 viral species. Observed data (left) and randomly permuted data (right) are shown. Axes are capped at 40 because most data points fell within this range.

To identify a collection of publicly recognized viral peptides from a VirScan analysis of 569 human sera samples (3.14), we chose the 5 most commonly recognized peptides from all viruses for which there were at least 5 seropositive individuals. This yielded a list of 363 viral peptides, 199 of which were recognized by at least 30% of seropositive individuals (Fig. 3.1A and table S3.1). These peptides were derived from 62 viral species spanning a broad range of viral classes and encompassed both structural and nonstructural proteins. Antibody responses to publicly recognized peptides appeared unrelated to donor age or geographic location and thus appeared to be a general feature of the human antibody response.

The publicly recognized viral peptides could harbor either a single epitope recognized by many different individuals or multiple epitopes. To distinguish between these possibilities and map individual epitopes more precisely, we designed an additional VirScan library containing tiled truncations and triple alanine-scan mutations of the 363 publicly recognized 56–amino acid (AA) viral peptides (Fig. 3.1B and table S3.2). We profiled the serum antibody responses of ~70 diverse donors with a wide range of viral exposures with this library and observed that the positions of the epitopes recognized by different individuals within these peptides were often identical (fig. S3.1, A and B).

### Antibodies recognizing public epitopes have biased light chain isotype usage

Next, we examined whether antibodies from different individuals that recognized the same public epitope were structurally similar. We adapted the VirScan assay to separately immunoprecipitated (IP) antibodies with kappa versus lambda light chains (“kappa antibodies” and “lambda antibodies,” respectively) (Fig. 3.1B). If different individuals made structurally similar antibodies against a given public epitope, we expected to detect responses to the epitope mainly in kappa or in lambda IP fractions, but if they made structurally diverse antibodies, we would expect no light chain bias. We reprofiled the ~70 human serum samples with the public epitope truncation and alanine-scan library using the kappa- and lambda-specific IP protocol and found that antibody responses to public epitopes were strongly biased in light chain isotype usage. For example, peptides from a 56-AA region of Human Herpesvirus 4 [Epstein-Barr virus (EBV)] were primarily recognized by lambda antibodies across individuals (Fig. 3.1C). Additionally, a 56-AA region from Rhinovirus B contained two distinct public epitopes, one predominantly recognized by lambda and the other by kappa antibodies across individuals (Fig. 3.1C and fig. S3.2). Overall, we observed an inverse distribution in which epitopes tended to be recognized in many donors’ lambda IP samples and few kappa IP samples, or vice versa (Fig. 3.1D). This contrasted with the distribution expected if there were no systematic light chain isotype bias (Fig. 3.1D and fig. S3.3). In the few cases where a peptide was recognized by many kappa and many lambda IP samples, distinct kappa and lambda epitopes were evident from the triple-alanine-scan data. Thus, across human populations, antibodies specific for a given public epitope frequently use the same light chain isotype, suggesting that they may share structural similarity. Moreover, light chains appear to be important for antibody recognition of public epitopes.

### Antibodies recognizing public epitopes exhibit similar high-resolution footprints

**Fig. 3.2.**
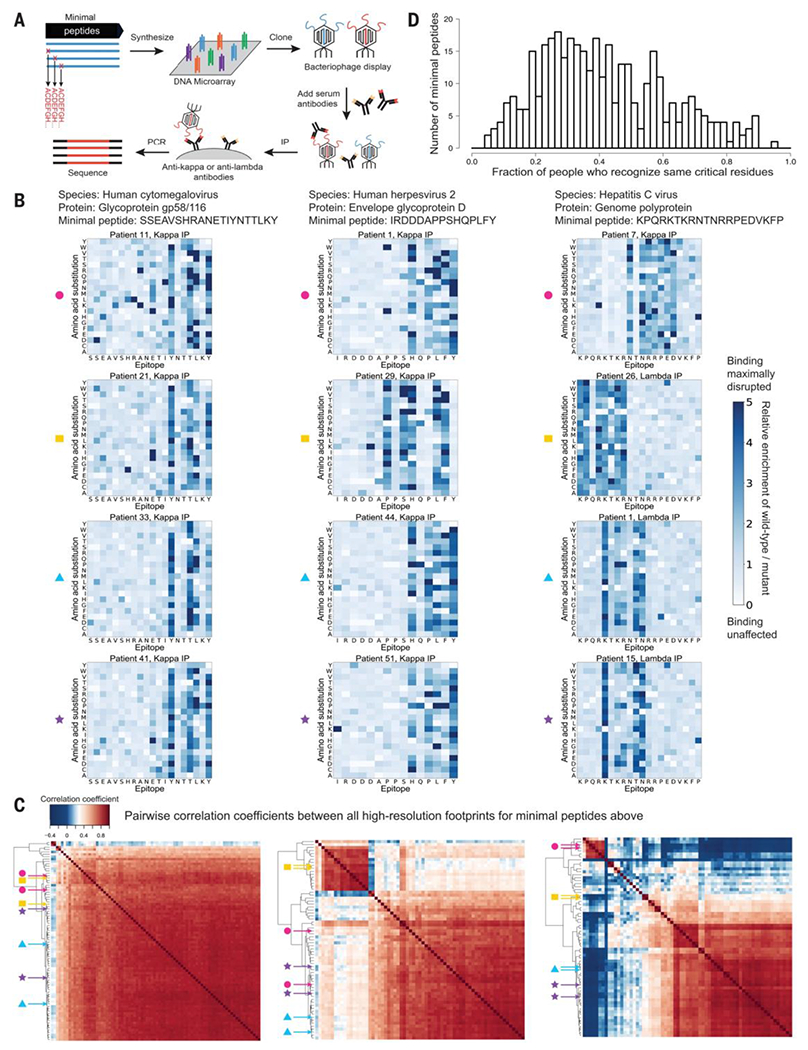
Antibodies recognizing public epitopes often exhibit similar high-resolution footprints. (**A**) Construction of the saturating mutagenesis public epitope library and VirScan screening procedure. Throughout, AAs are identified by their standard one-letter abbreviations. (**B**) Representative high-resolution antibody footprints from different individuals for minimal peptides from human cytomegalovirus (left), human herpesvirus 2 (center), and hepatitis C virus (right). The sequence of the minimal peptide is shown on the x axis and the AA substitution on the y axis. The darker the blue color, the greater the disruption of antibody binding. Colored shapes to the left side of each high-resolution footprint are coordinated with colored arrows in (C) to indicate the location of the high-resolution footprints and their technical replicates within the clustered heatmaps. (**C**) Clustered heatmaps illustrating the similarities between all high-resolution antibody footprints for the three example minimal peptides in (B); these were obtained by calculating the pairwise Pearson correlation coefficients between the enrichment matrices (see methods) of each serum sample that recognized the minimal peptide. Colored arrows are coordinated with colored shapes in (B) to indicate the location of the high-resolution footprints from (B) and their technical replicates within the clustered heatmaps. (**D**) Histogram depicting the fraction of individuals whose kappa or, separately, lambda antibody responses to a given minimal peptide recognized the consensus pattern of critical residues or only differed by one position.

Having found that different individuals recognize similar regions within publicly recognized 56-AA peptides, we next sought to map public epitopes at even higher resolution. We designed a VirScan library with 407 short peptide truncations (“minimal peptides”) that captured most of the antibody responses to the original 363 publicly recognized 56-AA peptides (Fig. 3.1C and table S3.3), as well as associated saturating mutants in which each AA was substituted with each of the other 19 possible AAs (3.19, 3.22) (Fig. 3.2A, fig. S3.4, and table S3.4). We profiled the ~70 human serum samples with the saturating mutagenesis public epitope library, using the kappa- and lambda-specific IP protocol, thus generating a set of high-resolution antibody footprints that identified residues critical for antibody recognition.

For most minimal peptides, different human serum samples produced high-resolution footprints that were similar (Fig. 3.2B). In many cases, most individuals’ high-resolution footprints for a given minimal peptide were highly correlated, often as much so as technical replicates. In some cases, two or more distinct groups of highly correlated high-resolution footprints for a given minimal peptide were evident. Thus, in many cases, different individuals appear to generate antibodies to precisely the same epitopes.

For each minimal peptide, we identified the dominant pattern of critical residues recognized by kappa or, separately, by lambda IP samples. This resulted in a set of 376 consensus viral public epitopes defined at AA resolution: 189 recognized by kappa antibodies (“kappa public epitopes”) and 187 recognized by lambda antibodies (“lambda public epitopes”) (tables S3.5 and S3.6). For 232 of the 376 consensus public epitopes, sera from more than one-third of individuals that recognized the minimal peptide targeted the same consensus pattern of critical residues (Fig. 3.2D and table S3.7). The public epitopes had an average of four critical residues and spanned an average of seven AAs from the first to last critical residue (tables S3.5 and S3.6).

Substitution of critical residues with chemically related AAs (3.23, 3.24) were frequently tolerated (e.g., A-S, I-L, I-V, Y-F) (fig. S3.5), with some exceptions: neither K-R nor D-E swaps were particularly well tolerated. Some differences in substitution tolerance in kappa versus lambda public epitopes were observed, (e.g., for W-F, W-Y, and A-P), suggesting different modes of binding certain AAs.

### Critical residues of public epitopes have a distinctive AA composition

**Fig. 3.3.**
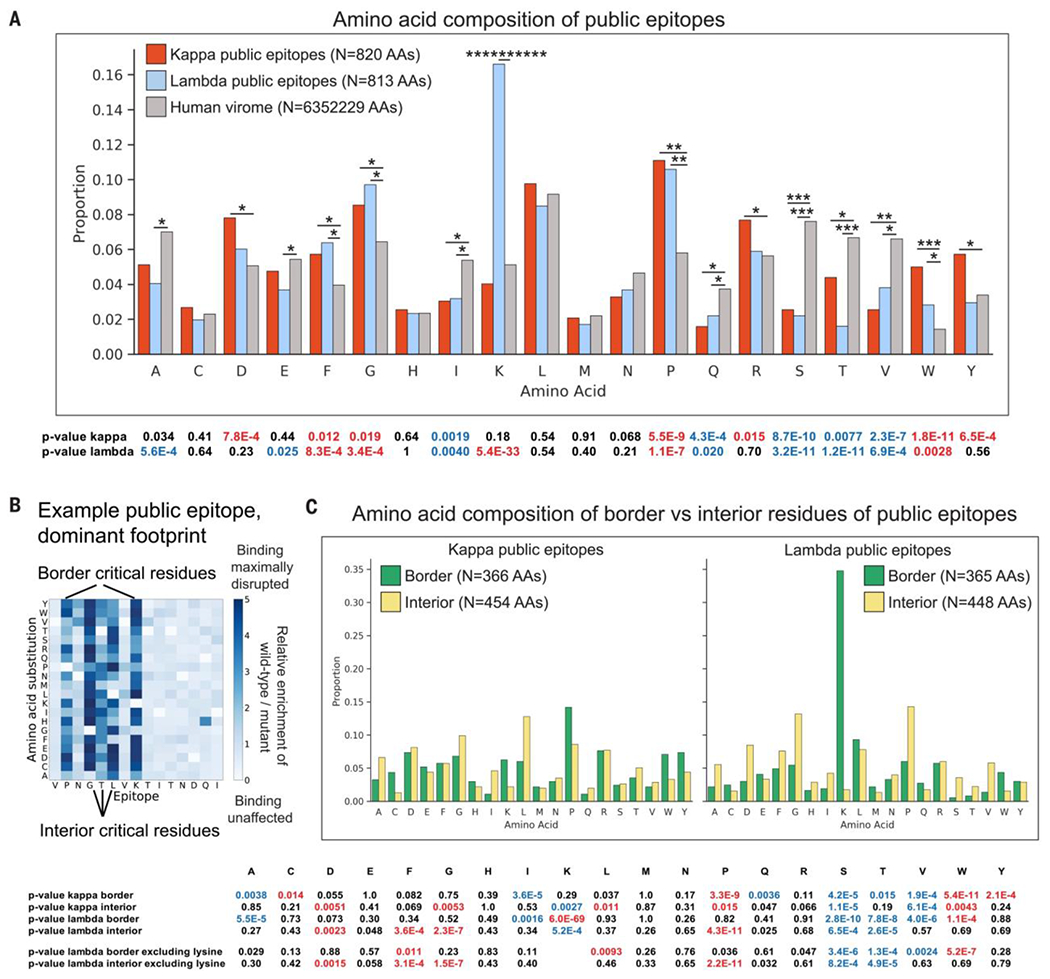
Critical residues of public epitopes have a distinctive AA composition, including profound enrichment of lysine at the borders of lambda public epitopes. (**A**) AA composition of critical residues of lambda and kappa public epitopes, relative to the entire human virome library. P values (binomial test) are listed below. Those that remain below the significance threshold of 0.05 after correcting for multiple hypothesis testing with the Benjamini–Hochberg false discovery rate method are indicated with asterisks in the bar chart (*P < 0.05, **P < 10–6, ***P < 10–9,**********P < 10–30) and colored in red (enriched AAs) or blue (depleted AAs). (**B**) Diagram indicating the positions of border and interior critical residues in a representative high-resolution antibody footprint. (**C**) AA composition of critical residues found at border or interior positions of kappa and lambda public epitopes. P values (binomial test) as in (A)are listed at the bottom of the figure.

The large number of consensus public epitopes we mapped allowed us to examine their AA composition, relative to the human viral proteome. Most notably, lysines were significantly enriched among critical residues of lambda (P < 5 x 10^−33^, binomial test) but not kappa public epitopes. Among other differences, prolines and tryptophans were strongly enriched, whereas serines, threonines, and valines were depleted in all public epitopes. (Fig. 3.3A). Although some of these differences may have resulted from the enrichment of particular residues on protein surfaces, others may have reflected preferential recognition of these AAs by kappa and/or lambda antibodies.

### The majority of lambda public epitopes have border lysine residues

Next, we investigated whether lysine residues were preferentially situated at particular position(s) within lambda public epitopes. We examined the frequency of each AA at border (first or last critical residues) or interior (all other critical residues) positions of public epitopes, relative to their frequency in the human viral proteome (Fig. 3.3, B and C). Several AAs were enriched or depleted at border or interior positions of kappa and/or lambda public epitopes. Most notably, lysine was enriched at border positions of public epitopes (enrichment P < 5 x 10^−69^). Of all 135 lysines in critical residues of lambda public epitopes, 127 were located at border positions and 61% of all lambda public epitopes featured a border lysine (table S3.6). We hypothesized that some lambda antibodies may harbor specificity for lysine.

### B cell receptors specific for three public epitopes exhibit conserved gene segment usage but distinct heavy chain CDR3 sequences

**Fig. 3.4.**
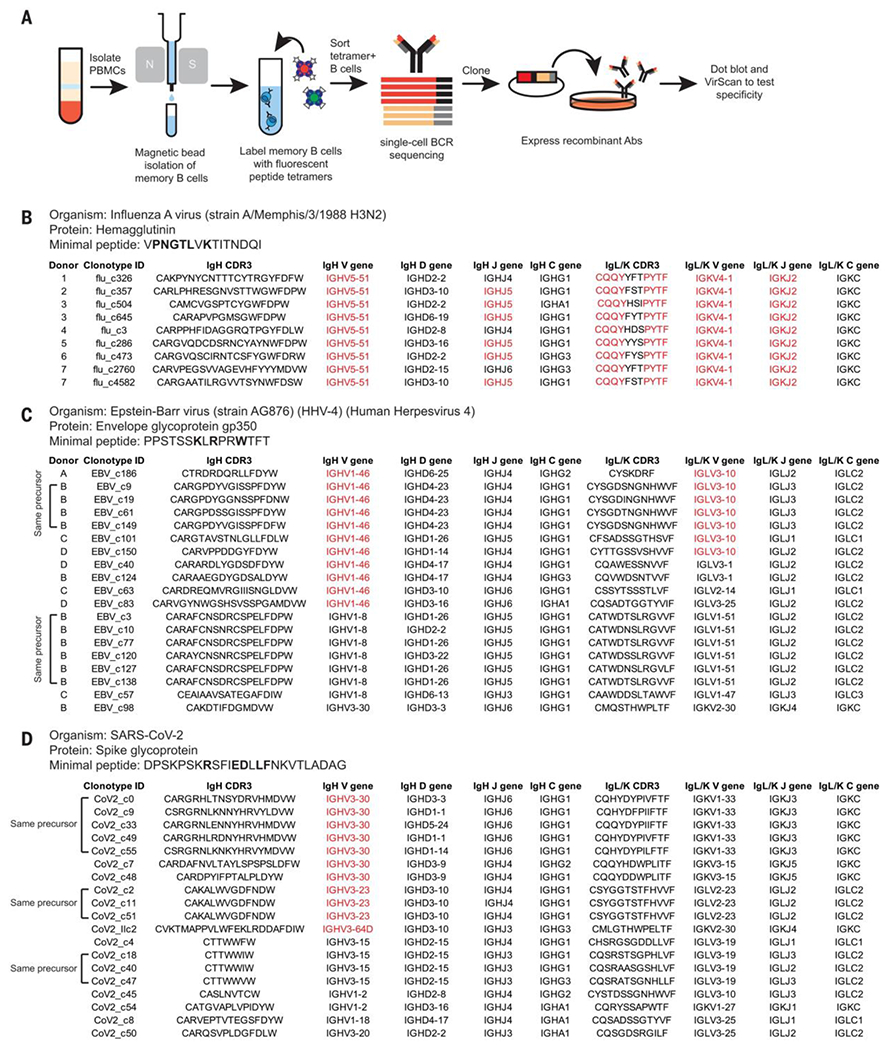
BCRs specific for example public epitopes share conserved gene segment usage but not heavy chain CDR3 sequences. (**A**) Schematic representation of the workflow to isolate public epitope-specific B cells. PBMCs were isolated from multiple healthy donors and pooled. Switched(IgG+ and IgA+) memory B cells were then purified by magnetic-activated cell sorting, split into aliquots to be labeled with customized CITE-Seq antibody barcodes, and then stained with fluorescent peptide tetramers. Fluorescent cells were isolated by FACS and analyzed by single-cell BCR sequencing. (**B to D**) Sequence characteristics of BCRs validated to bind three example minimal peptides. Consensus critical residues within the minimal peptides are shown in bold and conserved gene segments and CDR3 sequences are shown in red.

To explore sequence determinants of specificity for public epitopes, we initially selected two minimal peptides as case studies, both of which elicited highly conserved high-resolution antibody footprints across individuals: a kappa minimal peptide from influenza A hemagglutinin and a lambda minimal peptide from EBV gp350. We then isolated and sequenced B cell receptors (BCRs) specific for these peptides (Fig. 3.4A).

We obtained nine BCRs that recognized the influenza A minimal peptide from six donors (Fig. 3.4B; fig. S3.6, A to C; and table S3.8). All had kappa light chains and conserved gene segment usage: IgHV5-51 paired with IgKV4-1. They also featured similar light chain CDR3 sequences. The heavy chain CDR3 sequences were not conserved although they were longer than average (~20 versus ~15 AAs for the overall antibody repertoire) (3.25, 3.26). We profiled each of these antibodies with the saturating mutagenesis public epitope VirScan library and observed similar high-resolution footprints (fig. S3.7). All nine antibodies bound to intact influenza A H3 hemagglutinin trimers, but none were neutralizing (fig. S3.8).

We obtained 19 BCRs that recognized the EBV minimal peptide from four donors (Fig. 3.4C; fig. S3.6, A, D, E, and F; and table S3.9). Many shared conserved gene segment usage (IgHV1-46, frequently paired with IgLV3-10) but did not share conserved heavy chain CDR3 sequences. The IgHV1-46/IgLV3-10 BCRs from different donors exhibited almost identical high-resolution footprints (fig. S3.9), indicating that different individuals generate BCRs that recognize the EBV minimal peptide in extremely similar ways. A representative subset of the EBV minimal peptide-specific antibodies bound to full-length gp350 (fig. S3.10).

As a third case study, we isolated 19 BCRs that bound a publicly recognized SARS-CoV-2 spike peptide that overlaps with the fusion peptide (Fig. 3.4D) (3.18). These BCRs exhibited more diverse V gene segment usage than the flu and EBV BCRs. Nevertheless, 11 BCRs featured IgHV3 genes (IgHV3-30, IgHV3-23, and IgHV3-64D) and diverse heavy chain CDR3 sequences, and these BCRs exhibited very similar high-resolution footprints (fig. S3.11). Thus, IgHV3-30, IgHV3-23, and IgHV3-64D may share common features that enable recognition of the spike fusion peptide epitope. All 19 antibodies bound to the S2 domain of spike though only a few bound to full-length spike, and none out of a representative subset were neutralizing (fig. S3.12). This was consistent with reports of other antibodies that bound to this fusion peptide epitope but only when spike was engaged with ACE2 and thus constrained in the up conformation (3.27–3.29). From these three case studies, the theme of conserved V gene segment usage in the absence of the conserved heavy chain CDR3 sequence suggested that antibodies may recognize public epitopes through germline-encoded sequences within the V gene segments.

To examine potential polyspecificity of public epitope-reactive antibodies we profiled each of our influenza A-, SARS-CoV-2-, and a representative subset of our EBV minimal peptide-reactive antibodies against the human virome VirScan library (>100,000 peptides from >200 viral species) (3.15). Almost all the monoclonal antibodies we tested specifically bound peptides containing their cognate public epitope sequences; some cross-reacted with peptides that shared very similar sequences (fig. S3.13). Thus, the phenomenon of public epitopes is due to recurrently generated antibodies specific for these epitopes rather than polyreactive antibodies.

### A germline-encoded aspartic acid at position 51 of several lambda V gene segments drives specificity for border lysines

**Fig. 3.5.**
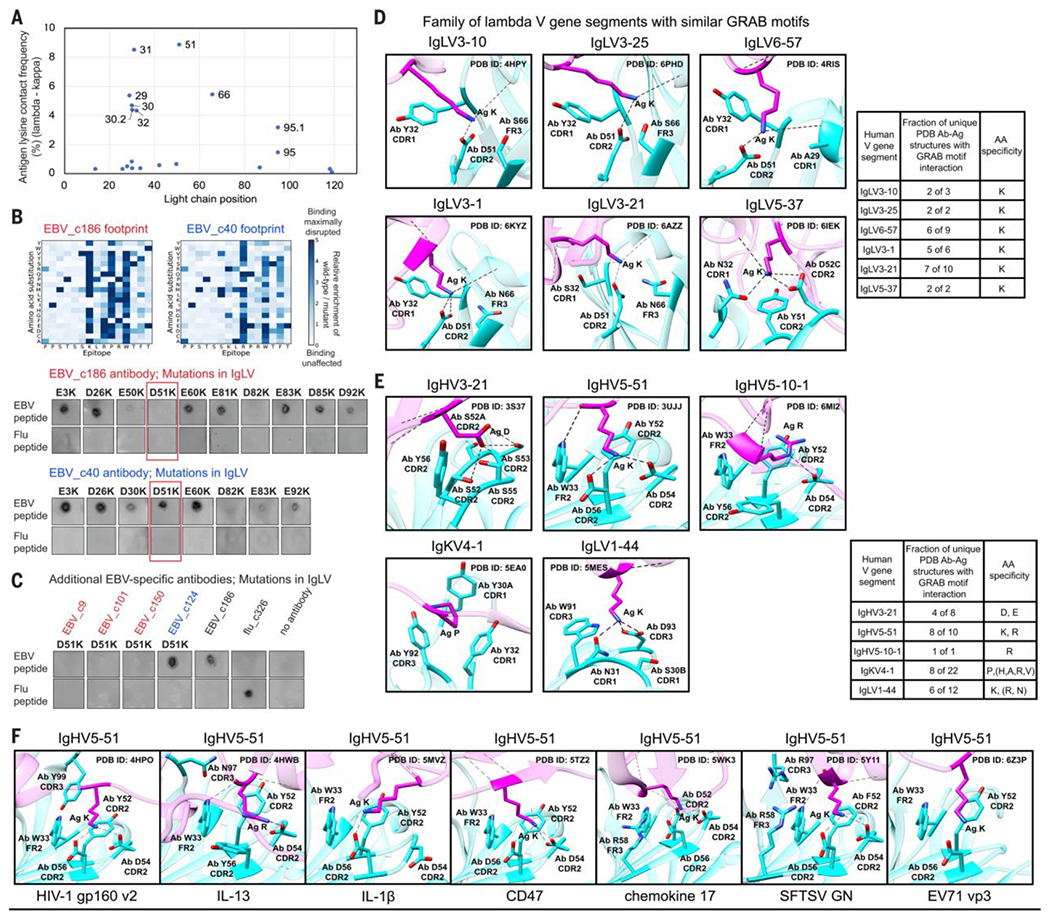
Many antibody V gene segments feature GRAB motifs that recurrently recognize specific AAs in antigens. (**A**) An aspartic acid germline-encoded at position 51 of several lambda V gene segments drives specificity for border lysines in epitopes. Differences in frequencies (lambda-kappa) with which each position of the light chain contacts lysine residues in the antigen in human lambda (n = 297) and kappa Ab-Ag complexes (n = 631) in the PDB. (**B and C**) The indicated mutations were introduced into a panel of EBV minimal peptide-specific antibodies and the impacts on antigen binding assayed by dot blot. The epitopes of antibodies shown in red include border lysines, whereas the epitopes of those shown in blue do not. EBV and influenza A minimal peptides (146786 and 72153 in table S3.3, respectively) were spotted on the nitrocellulose membranes; flu_c326 served as a control. (**D and E**) Representative GRAB motif interactions for the indicated human V gene segments. The antigen residue is shown in magenta and all antibody residues whose sidechains interact with the antigen residue are shown in cyan (FR, framework region). Summary tables (right) show the fraction of unique PDB Ab-Ag structures with the relevant V gene segment that feature the GRAB motif interaction. Throughout, PDB structures are visualized with UCSF Chimera (3.72). (**F**) Images of the other seven unique Ab-Ag structures involving IgHV5-51 that feature the GRAB motif interaction, labeled as in (D) and (E). Throughout, all residue positions follow the Chothia antibody numbering system. References for PDB Ab-Ag structures are provided in table S3.10.

The border lysine enrichment in lambda public epitopes suggested that lysine might specifically interact with lambda light chains, possibly through pairing with a germline-encoded acidic residue. We searched the Protein Data Bank (PDB) (3.30–3.32) for human lambda (n = 297) and kappa (n = 631) antibody-antigen (Ab-Ag) complexes and found that light chain position 51 directly interacted with lysines in antigens much more frequently in lambda than kappa Ab-Ag complexes (Fig. 3.5A). In these interactions, lambda light chain position 51 was almost always a germline-encoded aspartic acid.

We selected two antibodies that bound the same EBV minimal peptide described above but that recognized distinct critical residues: EBV_c186, which recognized a border lysine, and EBV_c40, which did not (Fig. 3.5B and fig. S3.9). We individually mutated each aspartic acid (D) or glutamic acid (E) of their lambda light chains to lysine (K) (for maximal disruption) and assessed impacts on binding by dot blot. The D51K mutation disrupted the binding of EBV_c186 but not EBV_c40, suggesting that D51 was important for border lysine recognition (Fig. 3.5B). The D51K mutation also disrupted the binding of three additional antibodies (EBV_c9, EBV_c101, and EBV_c150) whose epitopes within the EBV minimal peptide contained a border lysine, but had no effect on EBV_c124, whose epitope lacked a border lysine (Fig. 3.5C).

### At least six lambda V gene segments share similar germline-encoded lysine-specific binding motifs

Although a single salt bridge may stabilize an interaction, it alone cannot confer lysine specificity. To define additional residues involved, we investigated all Ab-Ag structures from the PDB with light chain position 51-antigen lysine interactions and uncovered a family of six lambda V gene segments (IgLV3-10, IgLV3-25, IgLV6-57, IgLV3-1, IgLV3-21, and IgLV5-37) that shared similar germline-encoded lysine-specific binding motifs. We called these germline-encoded amino acid–binding (GRAB) motifs (Fig. 3.5D and table S3.10). The GRAB motif in IgLV3-1 encompassed germline-encoded residues Y32 from CDR1, D51 from CDR2, and N66 from framework region 3 to specifically bind lysine in the antigen. D51 made a salt bridge with the lysine amine and Y32 made nonpolar interactions with the carbons of the side chain. Five of the six unique IgLV3-1 Ab-Ag complexes in the PDB featured this lysine-GRAB motif interaction (Fig. 3.5D and table S3.11, tab A). IgLV3-10, IgLV3-25, IgLV6-57, and IgLV3-21 harbored nearly identical lysine-specific GRAB motifs to IgLV3-1, whereas the IgLV5-37 GRAB motif differed somewhat, with Y51, D52C, and N32 making cation-pi, salt-bridge, and hydrogen bond interactions with the lysine amine, respectively. Cumulatively, 75% (24 of 32) PDB Ab-Ag structures involving these six V gene segments featured a lysine-GRAB motif interaction (Fig. 3.5D and table S3.11, tab A). Of these 24 structures, 16 involved conformational epitopes, indicating that GRAB motifs are important for recognition of both conformational and linear epitopes. Additionally, the lysine was almost always found at the edge of the epitope in both conformational and linear antigens (fig. S3.14 and table S3.11, tab A), possibly because these GRAB motifs are largely encoded by CDR1 and CDR2, the loops of which are oriented on the “outside” of the variable domain structure relative to the “interior” CDR3 loops. Alignment of Ab-Ag structures with the lysine-GRAB motif interaction showed similar interaction orientations (fig. S3.15A). Thus, IgLV lysine GRAB motifs encode specificity for border lysines and may drive the enrichment of lysine at the borders of lambda public epitopes, profoundly influencing the AA composition of public epitopes.

In addition to the six lambda V gene segments with lysine-specific GRAB motifs, four additional lambda V gene segments (IgLV3-9*02, IgLV3-16, IgLV3-22, and IgLV3-27) had germline-encoded Y/S32, D51, and S/T66 residues that were predicted by AlphaFold2 (3.33, 3.34) to fold into similar structures as the GRAB motifs described above (fig. S3.15B and table S3.11, tab C). However, there are currently no Ab-Ag complexes in the PDB involving these gene segments to confirm their specificity.

### Multiple heavy, kappa, and lambda V gene segments harbor GRAB motifs specific for particular AAs

The principle of GRAB motifs is not necessarily limited to lambda V gene segments or lysines. Therefore, we expanded our PDB analysis to search all human V gene segments (heavy, kappa, and lambda) for GRAB motifs that recurrently bound any given AA. We identified five additional GRAB motifs (Fig. 3.5E and table S3.11, tab B). For example, IgHV3-21 harbored a GRAB motif encompassing germline-encoded CDR2 residues (S52, S52A, S53, S55, and Y56) to specifically bind aspartate or glutamate in the antigen. A subset of the serine residues hydrogen-bonded with the carboxylate moiety whereas the tyrosine made nonpolar interactions with the carbons in the aspartate/glutamate side chain. Four of eight distinct PDB Ab-Ag complexes involving IgHV3-21 featured this aspartate/glutamate–GRAB motif interaction. The closely related IgHV3-11 had three alleles (*03, *05, and *06) that were not represented in the PDB but had germline-encoded S52, S52A, S53, S55, and Y56 residues predicted to form the same GRAB motif (fig. S3.15C and table S3.11, tab C).

IgHV5-51 encoded a GRAB motif comprising germline-encoded CDR2 and framework region 2 residues W33, Y52, D54, D56, and sometimes R58 that specifically interacted with a lysine in the antigen in 8 of the 10 distinct PDB Ab-Ag complexes involving IgHV5-51 (Fig. 3.5, E and F, and table S3.11, tab B). Similar to the IgLV lysine GRAB motif interactions, in the IgHV5-51 GRAB motif, D54 and D56 made salt bridges with the lysine amine whereas W33 and Y52 engaged in nonpolar interactions with the carbons of the side chain.

Some GRAB motifs exhibited recognition flexibility. IgKV4-1 harbored a GRAB motif involving Y30A, Y32, and Y92, germline-encoded by the CDR1 and CDR3, which formed nonpolar interactions with antigens. Although primarily recognizing proline (four examples), this GRAB motif could also interact with histidine, valine, arginine, or alanine (one example each), underscoring the chemical utility of tyrosine for protein-protein interactions. Of the 22 distinct PDB IgKV4-1 Ab-Ag complexes, 8 featured interactions between this GRAB motif and the AAs listed above (Fig. 3.5E and table S3.11, tab B).

If GRAB motif interactions were important for antigen recognition, they should contribute substantially to the DG of the Ab-Ag complex. Using computational alanine scanning (3.35, 3.36), we predicted the effects on the Ab-Ag complexes of mutating AAs recognized by GRAB motifs. The median predicted DDG was 1.9 kcal/mol and the median predicted fold change in binding affinity (KD) was 21.9, indicating that the GRAB motif interactions were important for Ab-Ag binding (table S3.11, tabs A and B).

We also observed five recurrent germline-encoded interactions present in distinct Ab-Ag structures involving the same antigen—evidence of shared antibody responses to public epitopes. For example, recent reports described a public antibody response to the receptor binding domain of SARS-CoV-2 spike that used IgHV3-53 or the closely related IgHV3-66, frequently paired with IgKV1-9 or IgKV3-20 (3.10, 3.11, 3.37). These antibodies had unmutated or nearly unmutated sequences, yet potently neutralized SARS-CoV-2 (3.38). We observed that IgHV3-53/IgHV3-66 engaged in multiple stereotyped interactions between germline-encoded residues fromCDR1, framework region 2, CDR2, and framework region 3 and Y473, the backbone near A475, and Y421 of spike (fig. S3.16 and table S3.11, tab D). Additionally, IgKV1-9/IgKV3-20 exhibited stereotyped interactions between germline-encoded residues of framework region 1, CDR1, framework region 3 (for IgKV3-20 only), and CDR3 and Y505 of spike (fig. S3.16 and table S3.11, tab D). These germline-encoded interactions appeared to contribute to the prevalence of the IgHV3-53/IgHV3-66 + IgKV1-9/IgKV3-20 neutralizing antibody response among individuals exposed to SARS-CoV-2 (3.10, 3.11), much in the way we hypothesize that GRAB motif interactions do. These stereotyped germline-encoded interactions were included in our count of GRAB motifs, with the caveat that their generalizability to other antigens was uncertain.

### GRAB motifs mediate recognition of an influenza A public epitope

**Fig. 3.6.**
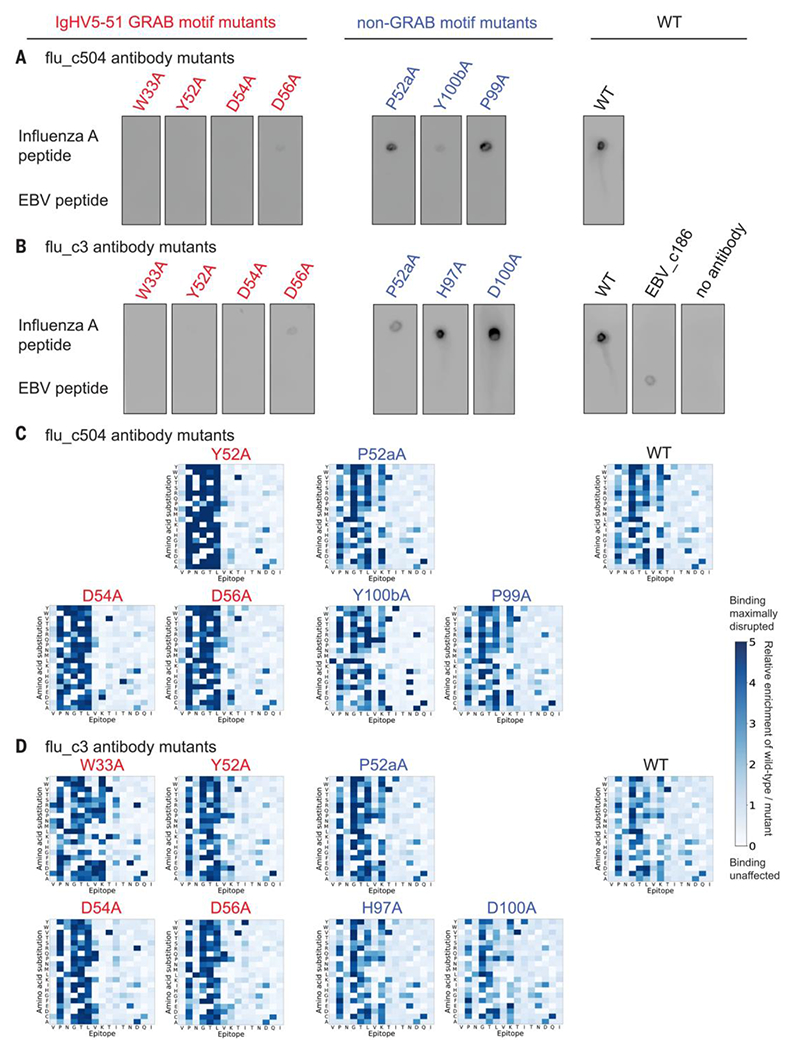
The IgHV5-51 GRAB motif mediates antibody recognition of an influenza A public epitope. (**A and B**) Mutation of IgHV5-51 GRAB motif residues in influenza A public epitope-specific antibodies severely weakens recognition of the epitope. The indicated mutations were introduced into two influenza A public epitope-specific antibodies, flu_c504 (A) and flu_c3 (B) and the impact on binding assayed by dot blot. Influenza A and EBV minimal peptides (146786 and 72153 in table S3.3, respectively) were spotted onto the nitrocellulose membranes; the EBV_c186 antibody served as a control. (**C and D**) High-resolution footprints for the monoclonal antibodies from (A and B). Heatmaps are labeled as described in Fig. 3.2B. Note that the heatmaps do not depict absolute enrichment values, but rather the relative enrichment of the wild-type peptide compared with the mutant peptide for a given sample.

If GRAB motifs mediate antibody recognition of public epitopes, mutation of GRAB motif residues should weaken this recognition. As a test case, we used influenza A public epitope-specific antibodies, because these shared conserved IgHV5-51 and IgKV4-1 gene segments, which we knew harbored GRAB motifs specific for lysine and proline, respectively. We individually mutated each AA of the IgHV5-51 and IgKV4-1 GRAB motifs to alanine in flu_c504 and flu_c3 and observed that these mutations often severely reduced binding, whereas mutations of nearby non–GRAB motif or of CDR3 residues often did not affect binding (Fig. 3.6, A and B, and fig. S3.17, A and B). We profiled the mutant versions of flu_c504 and flu_c3 using the saturating mutagenesis public epitope VirScan library. Use of substantial quantities of monoclonal antibody allowed us to obtain high-resolution antibody footprints for most mutants despite weakened binding. Although flu_c504 and flu_c3 originally recognized the critical residues P-GTL-K, mutation of IgHV5-51 GRAB motif residues specifically abolished recognition of the lysine while increasing the dependence on the other critical residues (Fig. 3.6, C and D). Likewise, IgKV4-1 GRAB motif mutations reduced proline recognition (fig. S3.17, C and D). In all cases, mutations outside of GRAB motifs did not affect the high-resolution antibody footprints. Thus, the IgHV5-51 and IgKV4-1 GRAB motifs likely mediate binding to the influenza A public epitope through specific recognition of lysine and proline, respectively, as predicted by the PDB analysis of these V gene segments.

### Public epitopes are largely species-specific

We next asked whether different species recognized the same or distinct public epitopes. We used VirScan to map antibody responses to peptides from SARS-CoV-2 spike in 30 SARS-CoV-2–infected humans (3.18), 9 SARS-CoV-2–infected nonhuman primates (NHPs) (3.39), and 8 C57BL/6 mice vaccinated with adeno-associated virus (AAV) encoding SARS-CoV-2 spike (3.40).

**Fig. 3.7.**
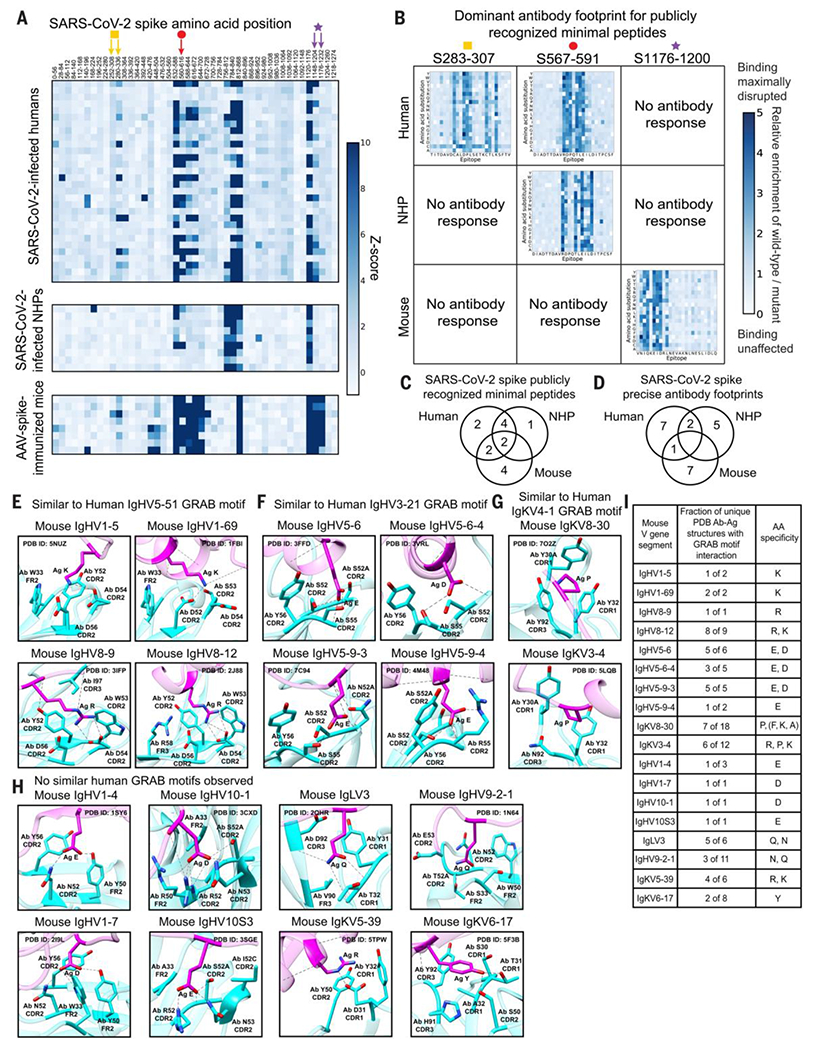
Public epitopes are largely species-specific, consistent with only partially overlapping sets of GRAB motifs. (**A**) Antibody responses to peptides fromSARS-CoV-2 spike in SARS-CoV-2-infected humans (n = 30) (3.18), SARS-CoV-2–infected NHPs (n = 9) (3.39), and C57BL/6 mice immunized with an AAV vector carrying stabilized prefusion SARS-CoV-2 spike (n = 8) (3.40). Each row represents a unique individual and each column represents a peptide tile. Darker colors indicate greater enrichment (Z-score) of a peptide in a given sample. Colored arrows are coordinated with colored shapes in (B) to show the positions of select public epitopes within spike. (**B**) Representative high-resolution footprints for minimal public epitope peptides recognized by one or more of the indicated species. Heatmaps are labeled as in Fig. 3.2B. Colored shapes are coordinated with colored arrows in (A)to show the positions of these public epitopes within spike. (**C and D**) Venn diagrams depicting the number of public epitopes recognized by one or more of the indicated species. (C) shows the number of publicly recognized minimal peptides, whereas (D) shows the number of precise antibody footprints (these were only considered to be shared if different species recognized the same pattern of critical residues). (**E to H**) Representative GRAB motif interactions for the indicated mouse V gene segments (table S3.13). Mouse GRAB motifs for which we found analogous human GRAB motifs are shown in (E) to (G). Those for which we did not find analogous human GRAB motifs are shown in (H). Images are labeled as in Fig. 3.5,D and E. (**I**) Summary table showing the fraction of unique PDB Ab-Ag structures with the relevant V gene segment that feature the GRAB motif interaction.

The general regions of spike recognized by all three species were similar (Fig. 3.7A), but when we reprofiled these samples using a SARS-CoV-2 public epitope saturating mutagenesis VirScan library (table S3.12), we observed that the precise public epitopes recognized by each species were most often distinct (Fig. 3.7, B to D, and fig. S3.18). Thus, mice and NHPs do not recapitulate the human antibody response to public epitopes.

### Different species have partially overlapping sets of GRAB motifs

We hypothesized that different species might recognize different public epitopes in part because of distinct sets of GRAB motifs. To investigate, we performed a similar analysis of PDB Ab-Ag complexes as described above, for mouse V gene segments (we analyzed V gene segments from several different mouse strains). This revealed 21 murine GRAB motifs, which partially overlapped with human GRAB motifs (Fig. 3.7, E to I, figs. S3.19 to S3.21, and table S3.13).

For example, four mouse V gene segments (denoted with “m”)—mIgHV1-5, mIgHV1-69, mIgHV8-9, and mIgHV8-12—had germline-encoded lysine/arginine-specific GRAB motifs resembling the human IgHV5-51 GRAB motif (Fig. 3.7, E and I, and table S3.13). Although the AAs (W, Y, D, and D) that constituted the human IgHV5-51, mIgHV8-9, and mIgHV8-12 GRAB motifs were equivalent, inmIgHV8-9 and mIgHV8-12, the W was encoded by the CDR2 whereas in human IgHV5-51 it was encoded by the framework region 2, illustrating convergent strategies to form similar GRAB motifs.

Several mouse GRAB motifs had no discernable human equivalents (Fig. 3.7, H and I, and table S3.13). These included similar aspartate/glutamate–specific GRAB motifs in mIgHV1-4 and mIgHV1-7, a distinct set of aspartate/glutamate-specific GRAB motifs in mIgHV10-1 and mIgHV10S3, asparagine/glutamine–specific GRAB motifs in mIgLV3 and mIgHV9-2-1, an arginine/lysine–specific GRAB motif in mIgKV5-39, and a tyrosine-specific GRAB motif in IgKV6-17, among others. Furthermore, mice have only three functional lambda V gene segments and these did not share the lysine GRAB motifs present in human lambda V gene segments. In several cases, additional mouse V gene segments shared conserved residues with known mouse GRAB motifs (table S3.13, tab B), but Ab-Ag structures were not present in the PDB to validate their specificity. Thus, only partially overlapping sets of GRAB motifs in mice and humans, potentially coupled with distinct CDR3 sequences and subtle differences in the positions of GRAB motif residues within CDR loops and framework regions, could affect the geometry of antibody binding and hence epitope selection, thereby explaining why human and mice antibodies rarely recognize the same public epitopes.

## Discussion

**Fig. 3.8.**
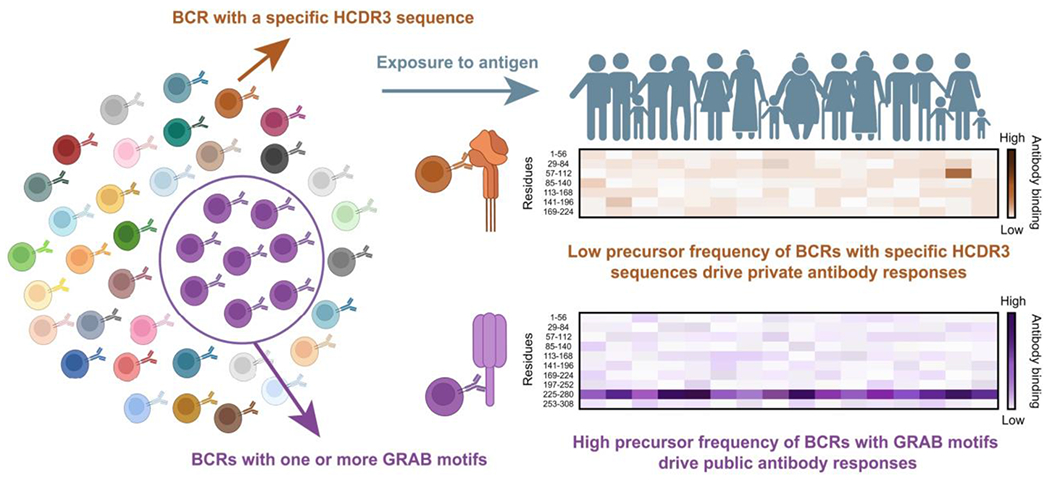
Proposed model for development of recurrent antibody responses to public epitopes through recognition by GRAB motifs. The B cell repertoire has a relatively low precursor frequency of BCRs with a specific heavy chain CDR3 (HCDR3) sequence, but a relatively high precursor frequency of BCRs with a certain combination of V gene segments (e.g., IgHV5-51 and IgKV4-1). If GRAB motifs within these V gene segments are sufficient to bind a certain epitope, this can lead to a public antibody response. By contrast, if a specific HCDR3 sequence is required to bind an epitope, this will likely lead to a private antibody response.

A fundamental question in immunology is why antibodies recognize particular regions of proteins more frequently than others. Our data support a model in which public epitopes arise in part because they are best aligned for recognition by GRAB motifs. A certain threshold binding energy is required to initiate an antibody response to an epitope. If a GRAB motif within a particular germline V gene segment provides a substantial portion of this binding energy, a larger number of CDR3 sequences would be compatible in the antibody because the CDR3 would need to contribute less binding energy to reach the threshold to progress to affinity maturation. Thus, there would be a relatively abundant precursor population of naïve B cells with adequate affinity for the epitope, and this could lead to an immunodominant, public antibody response (3.41, 3.42). Conversely, if a specific CDR3 sequence were required to provide most of the binding energy for a particular epitope, the antibody response to this epitope would be rarer because of the low precursor frequency of naïve B cells with this CDR3 sequence (Fig. 3.8). This model is supported by evidence of precursor frequency and binding affinity affecting antibody selection for VRC01-class HIV neutralizing antibodies from quantitative B cell transfer experiments in studies of immunodominance (3.43).

The evolution of antibody genes with germline-encoded sequences that bind AAs commonly found on the surface of proteins is clearly advantageous, allowing the immune system to recognize pathogens quickly and efficiently. However, given their prevalence, shared antibody responses can exert population-wide selective pressures on pathogens. This has been observed clearly for SARS-CoV-2: variants of concern have evolved to evade recognition by the public IgHV3-53/IgHV3-66 + IgKV1-9/IgKV3-20 class of neutralizing antibodies described above, among others (3.44–3.48). Alternatively, if public antibody responses are nonprotective to the host, in principle viruses could exploit this by conserving the cognate epitopes. They could also evolve additional non-neutralizing epitopes easily recognized by host GRAB motifs, thereby eliciting frequent nonprotective antibody responses across the host population and potentially delaying the production of more protective antibodies. Such epitopes could be removed from vaccine formulations.

An outstanding question is what selective pressure drove the expansion of lambda V gene segments with lysine-specific GRAB motifs in humans. Although mice do not share these GRAB motifs, 8 rhesus macaque lambda V gene segments share the same residues as known human lysine-specific GRAB motifs (3.49, 3.50) and as many as 39% (12 of 31, including hypothetical GRAB motifs) of the functional human lambda V gene segments have germline-encoded specificity for lysine. The expansion of lysine-specific GRAB motifs in primates suggests adaptation to pathogens, which in turn suggests an advantage for pathogens to be enriched in lysines. Notably, in the SARS-CoV-2 omicron BA.1 variant, 8 of the 30 AA substitutions in spike involve mutation to lysine (3.51). Recent modeling has suggested that positive charges on viruses may recruit heavily sialated mucins to enhance survival in aerosols or aid in interactions with lung surfaces (3.52, 3.53), providing a hypothesis for further study.

We have likely discovered only a fraction of all GRAB motifs as our analysis was limited by available PBD Ab-Ag structures (3.54). Indeed, 39% of human V gene segments (50 of 127 annotated by IMGT) were not represented and an additional 15% were only represented in one or two distinct Ab-Ag structures. Additional structural data and enhanced computational approaches will be needed to bridge this gap. Furthermore, GRAB motifs may have specificity for combinations of AAs or more complex topological structures, which were beyond the scope of our current analysis. Nevertheless, the GRAB motifs we identified—18 human, 21 mouse with structural evidence, and an additional 6 human and 27 mouse predicted GRAB motifs based on conservation to known motifs—likely influence the selection and composition of public epitopes, as illustrated by the profound enrichment of border lysines in lambda public epitopes and potentially also the enrichment of proline in kappa public epitopes.

This work has several implications: first, it suggests that private rather than public neutralizing antibodies may be superior candidates for inclusion in therapeutic monoclonal antibody cocktails, because private antibodies are less likely to exert population-wide selective pressures on pathogens and may thus retain efficacy for future variants. Second, the fact that public, immunodominant antibody responses appear to be largely species-specific may limit our ability to consistently predict how vaccines tested in nonhuman species will perform in humans, especially with respect to cross protection to variants. However, in another context species specificity may prove beneficial: vaccines administered to nonhuman species may elicit neutralizing antibody responses to epitopes that are not publicly recognized by humans. Because human viruses are not under evolutionary pressure to evade these antibodies, they may have therapeutic efficacy for humans against a broad range of variants. Third, the data presented here may enable exploration of the functional consequences of antibody responses to public epitopes, with relevance to vaccine design. Fourth, the set of viral public epitopesmay be useful in diagnostic applications. Fifth, knowledge of the GRAB motifs should aid species-specific B cell epitope prediction algorithms and computational methods to predict and design Ab-Ag interactions. Overall, this study reveals a fundamental structural code inherent in our humoral immune response that shapes epitope selection and composition and drives recurrent antibody responses across individuals and differing epitope selection among species, thus affecting host-pathogen coevolution and human health. Additionally, as T cell receptors are structurally similar to BCRs, it is highly likely that a similar structural code exists within T cell receptor V gene segments that contributes to T cell epitope immunodominance.

## Materials and Methods

### Human donor samples

Human specimens were collected in accordance with the local protocol governing human research after obtaining informed written consent from the donors. Secondary use of all human samples for the purposes of this work was exempted by the Brigham and Women’s Hospital Institutional Review Board (protocol number 2013P001337). Samples included serum and plasma from donors residing in Peru (n = 24), France (n = 2), and the United States (n = 52) (3.14). The United States cohort included donors with hepatitis C virus (n = 24), donors with human immunodeficiency virus 1 (n = 24), and healthy donors (n = 4). Human serum and plasma samples were stored in aliquots at −80°C until use. Apheresis leukoreduction collars from healthy platelet donors were obtained from the Brigham and Women’s Hospital Specimen Bank under protocol T0276. Access to COVID-19 patient samples was facilitated by the MassCPR.

### Design and cloning of the public epitope truncation and alanine scanning library

We designed peptide sequences of 15, 20, 25, 30, 35,40, and 45AAs in length, tiling through all of the 56-AA publicly recognized peptides with 5-AA overlap. For these shorter peptide truncations, we added random filler AA sequences after the stop codon so that downstream PCR steps would produce amplicons of the same size for all the members of the library. Additionally, we made triple-mutant sequences scanning through the 56-AA peptides. Non-alanine AAs were mutated to alanine, and alanines were mutated to glycine. We reverse translated the peptide sequences into DNA sequences that were (a) codon-optimized for expression in Escherichia coli, (b) lacked restriction sites used in downstream cloning steps (EcoRI and XhoI), and (c) were unique in the 50 nucleotides (nt) at the 5′ end to allow for unambiguous mapping of the sequencing reads. Then we added the adapter sequences AGGAATTCCGCTGCGT to the 5′ end and CAGGGAAGAGCTCGAA to the 3′ end or ATGAATTCGGAGCGGT to the 5′ end and CACTGCACTCGAGACA to the 3′ end to form the 200-nt oligonucleotide sequences, which were synthesized on a releasable DNAmicroarray (Agilent). We PCR-amplified the DNA oligonucleotide library with the primers T7-PFA 5′-AATGATACGGCGGGAATTCCGCTGCGT-3′ and T7-PRA 5′-CAAGCAGAAGACTCGAGCTCTTCCCTG-3′ and, separately, with the primers T7-Pep2-PFb 5′-AATGATACGGCGTGAATTCGGAGCGGT-3′ and T7-Pep2-PRb 5′-CAAGCAGAAGACGTCTCGAGTGCAGTG-3′, digested the products with EcoRI and XhoI, and cloned them into the EcoRI/SalI site of the T7FNS2 vector (3.17). We packaged the resultant library into T7 bacteriophage using the T7 Select Packaging Kit (EMD Millipore) and amplified the library according to the manufacturer’s protocol.

### VirScan

We performed VirScan (3.14–3.16), which is based on the phage immunoprecipitation and sequencing (PhIP-Seq) methodology (3.17), as described previously (3.14–3.16, 3.18) or with slight modifications. For the light chain isotype-specific IPs, we substitutedmagnetic protein A and protein G Dynabeads (Invitrogen) with 5 mg of biotinylated goat anti-human kappa (Southern Biotech) or 4 mg of biotinylated goat anti-human lambda (Southern Biotech) antibodies. The day after establishing the phage and serum mixtures, we added these antibodies and incubated the reactions overnight at 4°C. Afterward, we added 20 ml of Pierce streptavidin magnetic beads (Thermo Fisher Scientific), incubated the reactions for 4 hours at room temperature, then continued with the washing steps and the remainder of the protocol, as previously described (3.14–3.16, 3.18).

For the isotype-specific depletions, we substituted magnetic protein A and protein G Dynabeads with 15 mg of biotinylated goat anti-human kappa or 10 mg of biotinylated goat anti-human lambda antibodies. The day after establishing the phage and serum mixtures, we added these antibodies to the phage and serum mixtures and let the reactions incubate overnight at 4°C. We then added 60 ml (for kappa depletions) or 40 ml (for lambda depletions) of Pierce streptavidin magnetic beads, incubated the reactions for 4 hours at room temperature, then moved the supernatants into new plates. We added 40 ml of mixed protein A and protein G Dynabeads to the supernatants, incubated the reactions for 4 hours at room temperature, and continued with the IPs and library preparation for multiplexed Illumina sequencing as described previously (3.14–3.16, 3.18).

To test whether we could successfully profile antibody responses to the saturating mutagenesis public epitope library, we used the following antibodies against HA tag (which was included in the saturating mutagenesis public epitope library as a control): mouse anti-HA-biotin clone HA-7 (Sigma), rat anti-HA-biotin clone 3F10 (Sigma), and anti-HA magnetic beads clone 2-2.2.14 (Thermo Fisher Scientific) (fig. S3.4).

For mouse serum samples, 0.6 ml of mouse serum was used for each VirScan reaction and 40 ml of mixed protein A and protein G Dynabeads were used as the IP reagent. For NHP samples, 0.2 ml of NHP serum was used for each VirScan reaction and 40 ml of mixed protein A and protein G Dynabeads were used as the IP reagent. For monoclonal antibodies, ~50 ml of cell culture supernatant was used as input for VirScan reactions involving the saturating mutagenesis public epitope library to generate high-resolution footprints. Unless otherwise specified, 20 ng of purified antibody was used as input for VirScan reactions involving the human virome library to investigate potential polyspecificity. Forty microliters of mixed protein A and protein G Dynabeads were used as the IP reagent. All samples were run in duplicate except for the mouse sera samples profiled with the CoV 56-AA library (however, this library contains duplicate barcoded versions of each peptide, and the measurements for each of the duplicate peptides were averaged).

### Statistical analysis of VirScan data generated with the public epitope truncation and alanine scanning library

We first mapped the sequencing reads to the reference library sequences using Bowtie (3.55) and counted the number of reads corresponding to each peptide in the input library and each sample “output”. For each sample, we normalized the read counts for each peptide by the total read counts for the sample. Then, we divided the normalized read counts of each peptide in the sample by the normalized read counts of each peptide in the input library to obtain an enrichment value. We averaged enrichment values for technical replicates of a sample.

For each truncation and alanine scanning mutant peptide, we calculated relative-to-wildtype enrichment values as follows: we first calculated the average enrichment value of the middle 50% of the alanine scanning mutants of a given 56-AA peptide, as we assumed most alanine scanning mutations throughout the peptide would not disrupt the epitope. We found this to be a more robust (i.e., less noisy) representation of the enrichment of the wild-type 56-AA peptide than the enrichment value of the single wild-type 56-AA peptide. Next, we divided the enrichment value of a given peptide truncation or alanine scanning mutant by the average enrichment value of the middle 50% of the alanine scanning mutants to obtain a relative-to-wild-type enrichment value. Finally, for each 56-AA peptide, we generated a heatmap using Python matplotlib to illustrate the relative-to-wild-type enrichment values of the peptide truncations and alanine scanning mutants for all the samples that recognized the wild-type version of the 56-AA peptide (i.e., where the enrichment value of the wildtype 56-AA peptide was >1.5). For the alanine scanning mutants, the values in the heatmap were 1 / (relative-to-wild-type enrichment value), with darker blue colors indicating greater disruption of the epitope.

### Permutation analysis

We limited this analysis to the short peptide truncations (15, 20, 25, and 30-AA in length) as some 56-AA peptides contained more than one distinct public epitope, and we sought to isolate these with the shorter peptides. To perform one permutation, we randomized the kappa and lambda assignments of the pair of IPs for each serum sample. We then counted the number of kappa and lambda IP fractions in which each short peptide truncation was enriched. We performed a total of 1000 permutations. Based on these permutations, we calculated an average distribution of kappa and lambda IP samples in which the short peptide truncations were expected to score, and used this distribution to calculate the fold-enrichment of each value in the observed distribution. We also calculated a P-value for each observed value based on how many random permutations had resulted in at least such a high number of peptides scoring in the given numbers of kappa and lambda IP samples.

### Design and cloning of the saturating mutagenesis public epitope library

From the VirScan data generated with the public epitope truncation and alanine scanning library using the kappa and lambda isotype-specific IP protocol, we identified peptide truncations whose relative-to-wild-type enrichment values were at least 0.75 in at least half of the samples in which the wild-type 56-AA peptide scored. We filtered for peptide truncations with read counts of at least 5 in the input library to avoid spurious enrichment values, and peptide truncations for which at least 6 samples recognized the wild-type 56-AA peptide. We first chose 15-AA and 20-AA peptide truncations that met these requirements. This was a relatively stringent set of criteria, so to capture the remaining epitopes within the 56-AA peptides of the public epitope truncation and alanine scanning library, we next set the threshold for the relative-to-wild-type enrichment values to 0.5 and chose the shortest peptide truncation that captured at least half of the samples’ responses to the wild-type 56-AA peptide.

With this list of minimal public epitope-containing peptides (“minimal peptides”), we designed saturating mutants such that each AA of the peptide was mutated to the other 19 AAs. As a positive control and to calibrate how antibody responses to saturating mutants would be detected in a VirScan assay, we included HA tag and saturating mutants of this epitope. Because the public epitope peptides were of varying sizes, we added random filler AA sequences after the stop codon so that downstream PCR steps would yield products of the same size for all the members of the library. We reverse-translated the peptide sequences into DNA sequences that were codon-optimized for expression in E. coli, that lacked restriction sites used in downstream cloning steps (EcoRI and XhoI), and that were unique in the 50 nt at the 5′ end to allow for unambiguous mapping of the sequencing reads. Then we added the adapter sequence GGAATTCCGCTGCGT to the 5′ end and CAGG-GAAGAGCTCGA to the 3′ end to form the 198-nt oligonucleotide sequences. These oligonucleotide sequences were synthesized on a releasable DNA microarray (Agilent). We PCR-amplified the DNA oligonucleotide library with the primers T7-PFA 5′-AATGATACGGCGGGAATTCCGCTGCGT-3′ and T7-PRA 5′-CAAGCAGAAGACTCGAGCTCTTCCCTG-3′, digested the product with EcoRI and XhoI, and cloned it into the EcoRI/SalI site of the T7FNS2 vector (3.17).We packaged the resultant library into T7 bacteriophage using the T7 Select Packaging Kit (EMD Millipore) and amplified the library according to the manufacturer’s protocol.

### Statistical analysis of VirScan data generated with the saturating mutagenesis public epitope library and the SARS-CoV-2 public epitope saturating mutagenesis library

We first mapped the sequencing reads to the reference library sequences using Bowtie (3.55) and determined the read counts of each peptide in the input library and each sample “output”. For each sample, we normalized the read counts corresponding to each peptide by the total read counts for the sample. Then, for each peptide, we divided the normalized read counts of each peptide in the sample by the normalized read counts of each peptide in the input library in order to obtain the enrichment value.

For each minimal peptide and for each sample, we next calculated relative-to-wild-type enrichment values for each AA substitution mutant as follows: we first calculated the average enrichment value of the middle 50%of the alanine substitution mutants, most of which we assumed would be minimally disruptive to the epitope contained in the peptide. We found that the average enrichment value of the middle 50% of the alanine substitution mutants was a more robust representation of the enrichment of the wild-type peptide than the enrichment value of the single wildtype peptide. Next, we divided the enrichment value of a given substitution mutant by the average enrichment value of the middle 50% of the alanine substitution mutants in order to obtain a relative-to-wild-type enrichment value. Finally, for each minimal peptide, if a sample recognized the wild-type version of the peptide (i.e., the enrichment value of the wildtype peptide was >1 and the average enrichment value of the middle 50% of all substitution mutants of the peptide was >1), we then generated an “enrichment matrix” with the relative-to-wild-type enrichment values of all the substitution mutants of the peptide. We also generated a heatmap using Python matplotlib displaying values of 1 / (relative-to-wild-type enrichment value + 0.2) for all the substitution mutants of the peptide.

### Statistical analysis of VirScan data generated with the human virome library and the CoV 56-AA library

VirScan data generated with the human virome library and the CoV 56-AA library were analyzed as previously described (3.15, 3.16, 3.18).

### Critical residue analysis and definition of kappa and lambda public epitopes

For every minimal peptide in the saturating mutagenesis public epitope library, we converted all enrichment matrices from samples that recognized the given peptide into binary matrices: if the relative-to-wild-type enrichment value of a given mutant peptide was <0.5 (i.e., the mutant enriched less than half as well as the middle 50% of all alanine substitution mutants for that peptide), then the mutant was considered to disrupt the epitope and given a value of “1”. Alternatively, the mutant was considered to be permitted and given a value of “0”. Next, we collapsed each binary enrichment matrix into a one-row summary by adding the number of mutants at each position that disrupted the epitope. Then, for each minimal peptide, we converted the one-row summaries into binary one-row summaries: if at least one third of the 19 substitutions at a given position disrupted the epitope, then the position was considered a critical residue and given a value of “1”. Alternatively, the position was given a value of “0”. These data are available on the Harvard Dataverse, doi: 10.7910/DVN/AIXWW2. Next, for each minimal peptide, we counted the number of samples that exhibited the same binary summaries (i.e., that recognized the same pattern of critical residues). The pattern of critical residues shared by the greatest number of samples was considered to be the consensus public epitope, also called the dominant footprint. Thus, we defined the critical residues of the kappa and lambda public epitopes. Samples that recognized the consensus public epitope or a pattern of critical residues that only differed from the consensus public epitope by one position were considered to be part of the dominant footprint group. The counts and proportions of kappa or lambda IP samples that recognized each minimal peptide and that were part of the dominant footprint group are provided in table S3.7. We performed this analysis separately for kappa samples and lambda samples and limited the analysis to minimal peptides recognized by at least five kappa samples or at least five lambda samples.

### Analysis of tolerated AA substitutions

For each minimal peptide, we took the average of all the binary matrices for samples that were part of the dominant footprint group. Then, for every critical residue of a given public epitope, we determined which AA substitutions were permitted (i.e., the AA substitutions for which the average of the binary matrices was >0.5). Finally, for each of the 20 AAs, we calculated the frequency at which each of the other 19 AA substitutions were permitted at a critical residue.

### Public epitope-specific memory B cell isolation and sequencing

For most experiments, ~10 fresh (<6 hours from collection) apheresis leukoreduction collars from healthy platelet donors were obtained from the Brigham and Women’s Hospital Specimen Bank under protocol T0276. For the experiment to isolate SARS-CoV-2 public epitope-specific memory B cells, five cryopreserved peripheral blood mononuclear cell (PBMC) samples and one fresh leukopak sample from COVID-19-recovered donors were purchased from Cellero and obtained from the MassCPR COVID-19 Biorepository, respectively. PBMCs were purified on a Ficoll-Paque density gradient. Briefly, 8ml of donor blood was diluted 1:1 with PBS, slowly layered on 16 ml of Ficoll-Paque (Thermo Fisher Scientific) and centrifuged at 400g for 30 min with the brake off. The upper layer containing plasma and platelets was removed and frozen, and the mononuclear cell layer at the interface was extracted and washed four times with PBS at 400g for 10 min with the brake on. PBMCs from the different apheresis collars were counted, pooled together, and switched memory B cells were purified using the Human Switched Memory B cell Kit (Miltenyi) according to the manufacturer’s instructions. We used this kit, which employs a negative-selection protocol, rather than a kit that positively selects for IgG+ memory B cells, to avoid labeling the BCR with an antibody and potentially influencing the ability of the BCR to bind the viral peptide. Purified memory B cells were resuspended in RPMI 1640 (Life Technologies) with 10% (v/v) FBS (Hyclone), 100 U/ml of penicillin, 100 mg/ml of streptomycin, and incubated at 37°C and 5% CO2 for a few hours (≤6 hours, to ensure high cell viability). Memory B cells were stained with biotinylated minimal peptides conjugated to fluoresceinated streptavidin and then fluorescent cells were isolated by FACS using the MoFlo Astrios EQ Cell Sorter (Beckman Coulter) (3.56) (Fig. 3.4A). We typically began with 8x10^9^ PBMCs from ~10 donors and ultimately purified 5x10^7^ switched memory B cells. In cases where we wanted to sort for multiple minimal peptide specificities, we split the switched memory B cells into multiple aliquots, and labeled the different aliquots with distinct CITE-seq barcodes (3.57) customized to be compatible with the Chromium 5′ V(D)J solution (10x Genomics). We sorted ~0.002% of the switched memory B cells for any given minimal peptide specificity, then sequenced their BCRs using the Chromium 5′ V(D)J solution (10x Genomics) according to the manufacturer’s instructions. In addition to amplifying and sequencing the BCR transcripts, we designed custom primers to amplify MHC transcripts and the customized CITE-seq barcodes (3.58, 3.59). SARS-CoV-2 public epitope-reactive BCRs were sequenced using the Chromium Next GEM Single Cell 5′ Kit v2 (10x Genomics). MHC transcripts were not sequenced for these cells.

Minimal peptides used for staining and sorting memory B cells were designed to have an N-terminal biotin or biotin-Ahx modification followed by a GGGGS linker sequence, and neutral charge. If a minimal peptide sequence was not neutral, the linker was extended with charged AAs to achieve net neutral charge. Biotinylated peptides were ordered from Thermo Fisher Scientific or GenScript, reconstituted in a small amount of DMSO (20–40 ml, or 2 to 4% of the final volume), then diluted to a final concentration of 1 mg/ml in ultrapure water and stored at −20°C in aliquots. The sequences of the biotinylated public epitope peptides were as follows:

146786_neutral_linker_flu: N terminus-Biotin-Ahx-GGGGSVPNGTLVKTITNDQI-C terminus

72153_neutral_linker_EBV: N terminus-Biotin-Ahx-EGEGGGGSPPSTSSKLRPRWTFT-C terminus

SARS-CoV-2_S807-832: N terminus-Biotin-GGGGSDPSKPSKRSFIEDLLFNKVTLADAG-C terminus

Biotinylated peptides were conjugated to fluorescent streptavidin by combining the reagents in the following ratios:

For a 20-AA peptide:

6.3 mg of public epitope peptide: 9.0 mg of streptavidin-APC (Thermo Fisher Scientific)

19 mg of public epitope peptide: 9.5 mg of steptavidin-488 (Thermo Fisher Scientific)

3.9 mg of irrelevant peptide: 10.5 mg of streptavidin-PE (Thermo Fisher Scientific)

3.3 mg of irrelevant peptide: 10 mg of streptavidin-BV421 (Biolegend)

For a 25-AA biotinylated peptide:

7.7 mg of public epitope peptide: 9.0 mg of streptavidin-APC

23.4 mg of public epitope peptide: 9.5 mg of streptavidin-488

4.7 mg of irrelevant peptide: 10.5 mg of streptavidin-PE

4.0 mg of irrelevant peptide: 10 mg of streptavidin-BV421

Streptavidin-peptide complexes were incubated at 4°C for ~4 hours on a rotator, then purified using a Bio-Spin^®^ P-30 Gel Column into Tris Buffer (Bio-Rad) according to the manufacturer’s instructions.

Immediately prior to staining switched memory B cells, two customized CITE-Seq ADTs per memory B cell aliquot were pooled and cleaned on a 50 kDa cutoff column as previously described (3.57). Switched memory B cells were centrifuged at 300g for 10 min, then washed once with 1ml of staining buffer (PBS + 2% BSA + 0.02% Tween 20) and centrifuged at 400g for 4 min. Next, cells were resuspended in 100 ml of staining buffer and 100 ml of cleaned ADT pool (containing ~1–2 mg of each ADT) and incubated with end-over-end mixing for 30 min at 4°C. ADT-labeled switched memory B cells were washed once with staining buffer, then resuspended in purified streptavidin-peptide complexes plus 150 ml of staining buffer and incubated with end-over-end-mixing for 1 hour at 4°C. Then, cells were centrifuged at 400g for 4 min, washed twice in staining buffer, resuspended in 750 ml of staining buffer, and filtered over a 35-mm nylon mesh cell strainer. Cells that were negative for the two fluorophores conjugated to irrelevant peptides and positive for the two fluorophores conjugated to the minimal public epitope peptide were sorted using the MoFlo Astrios EQ Cell Sorter (Beckman Coulter) into 4 ml of RPMI-1640 supplemented with 0.2% BSA, 100 U/ml of penicillin, 100 mg/ml of streptomycin in one well of a 96-well plate. After sorting, cells were immediately used as input for single-cell BCR sequencing using the Chromium 5′ V(D)J solution (10x Genomics) according to the manufacturer’s protocol with slight modifications (described below) to amplify ADT barcodes and MHC transcripts in addition to BCR transcripts. V, D, and J gene segments assigned by Cell Ranger (10x Genomics) were double checked by entering the nucleotide sequence of the BCR variable region as a query sequence in IgBlast (3.60), using the IMGThumanV, D, and J (F + ORF) germline gene databases. Where the IgBlast and Cell Ranger gene segment assignments differed, the IgBlast assignments were used.

### CITE-Seq ADT customization for Chromium 5′ V(D)J solution (10x Genomics)

ADT barcodes were designed as follows to be compatible with the Chromium 5′ V(D)J solution (10x Genomics):

5′ to 3′: 4 nt linker–10xVDJ_ADT_inner primer binding site–Read 2 adaptor sequence–random barcode–13 nt homology to the template switch oligo (10x Genomics)

An example sequence is given below:

5′- /5AmMC12/ATCT–GCGTTCGAGCTCTTCCCTG–GTGACTGGAGTTCAGACGTGTGCTCTTCCGATCT–ATGGACCTTAAGCGCTACCGGAATGGTTCG–CCCATATAAGAAA −3′

This design allowed for a two-step PCR enrichment, the first step using SI-PCR primer (10x Genomics) and 10xVDJ_ADT_inner, and the second using SI-PCR primer and a Sample Index PCR primer (10x Genomics).

After the cDNA amplification step of the Chromium 5′ V(D)J solution (10x Genomics) protocol, amplification products above 400 bp, including MHC and BCR transcripts, were captured on SPRI beads (Beckman Coulter) using 0.6X SPRI. The supernatant containing amplification products under 400 bp, including ADT barcodes, was removed to a separate tube, 1.4X SPRI was added to obtain a final SPRI volume of 2X SPRI, and purified in parallel with the amplification products over 400 bp. ADT Target Enrichment 1 from Amp cDNA was performed using SI-PCR primer and 10xV(D)J_ADT_inner primer. Amplification products from nine cycles of PCR were purified with 2X SPRI. ADTs were indexed and purified using the conditions given by the Chromium 5′ V(D)J solution (10x Genomics) protocol. The Sample Index PCR served both to index the ADTs and to perform a second step of target enrichment. The sequence of the 10xV(D)J_ADT_inner primer was as follows:

10xV(D)J_ADT_inner: 5′-GCGTTCGAGCTCTTCCCTG-3′

After quantification, libraries were mixed (50% BCR transcripts, 25% MHC I transcripts, 12.5% MHC II transcripts, 12.5% ADT barcodes). Sequencing was performed with a NextSeq 500 (Illumina) per manufacturer’s instructions.

Next-generation sequencing reads corresponding to ADTs were separated by cell barcode. For each cell’s ADT reads, the number of times each ADT barcode appeared was counted. The ADT pair with the greatest counts indicated the peptide for which the cell was sorted.

### MHC enrichment primer design and donor identification strategy

Primers to amplify MHC I and DR transcripts were designed by downloading CDS sequences of all HLA-A, HLA-B, HLA-C, DRA, and DRB alleles from the Immuno Polymorphism Database-ImMunoGeneTics information system ^®^ / Human Leukocyte Antigen (IPD-IMGT/HLA) (3.58, 3.59), aligning all alleles of each gene, and designing primers in conserved regions to cover over 95% of alleles and produce reverse transcription products whose lengths would be compatible with the downstream Chromium 5′ V(D)J solution (10x Genomics) protocol. The sequences of the primers designed to amplify the MHC transcripts were as follows:

HLA_A_Outer_1_346 bp: 5′-CAGGGCGATGTAATCCTTGC-3′

HLA_A_Outer_2_450: 5′-CAAGGCGATGTAATCCTTGC-3′

HLA_B_Outer_385bp: 5′-TCCTCGTTCAGGGCGATGT-3′

HLA_C_Outer_360bp: 5′-GCGATGTAATCCTTGCCGTC-3′

HLA_A_Inner_1_346bp: 5′-AACCGGCCTCGCTCTGG-3′

HLA_A_Inner_2_450bp: 5′-GAACCGTCCTCGCTCTGGT-3′

HLA_B_Inner_279bp: 5′-TGTGAGACCCGGCCTCG-3′

HLA_C_Inner_250bp: 5′-CTCGCTCTGGTTGTAGTAGC-3′

DRA_outer: 5′-ATGAAACAGATGAGGACG-3′

DRB_outer_1: 5′-CTCGCCGCTGCACTGTG-3′

DRB_outer_2: 5′-CCCCGTAGTTGTGTCTGCA-3′

DRA_inner: 5′-CTCTCTCAGTTCCACAGGGC-3′

DRB_inner_1: 5′-CCCAGCTCCGTCACCGC-3′

DRB_inner_2: 5′-GTCCTTCTGGCTGTTCCAG-3′

MHC I Target Enrichment 1 from Amp cDNA was performed using SI-PCR primer and MHC I PCR1 RV “outer” mixture (consisting of HLA-A outer 1, HLA-A outer 2, HLA-B outer, HLA-C outer). Amplification products from 10 cycles of PCR were purified with 0.8X SPRI. MHC II Target Enrichment 1 from Amp cDNA was performed using SI-PCR primer and MHC II PCR1 RV “outer” mixture (consisting of DRA_outer, DRB_outer_1, and DRB_outer_2). Amplification products from 10 cycles of PCR were purified with 0.8X SPRI. MHC I Target Enrichment 2 was performed using 10xV(D)J_PCR2F primer and MHC I inner primer mix 2 (consisting of HLA-A inner 1, HLA-A inner 2, HLA-B inner, HLA-C inner). Amplification products from 10 cycles of PCR were purified with a double-sided size selection using 0.5X SPRI and 0.8X SPRI. MHC II Target Enrichment 2 was performed using 10xV(D)J_PCR2F primer and MHC II inner primer mix 2 (consisting of DRA_inner, DRB_inner_1, and DRB_inner_2). Amplification products from 10 cycles of PCR were purified with a double-sided size selection using 0.5X SPRI and 0.8X SPRI. MHCI and MHCII samples were indexed and purified using the conditions given by the Chromium 5′ V(D)J solution (10x Genomics) protocol. The sequence of the 10xV(D)J_PCR2F is as follows:

10xV(D)J_PCR2F: 5′-AATGATACGGCGACCACCGAGATCT-3′

After quantification, libraries were mixed (50% BCR transcripts, 25% MHC I transcripts, 12.5% MHC II transcripts, 12.5% ADT barcodes). Sequencing was performed with a NextSeq 500 (Illumina) per manufacturer’s instructions.

Next-generation sequencing reads corresponding to MHC I and MHC II transcripts were separated by cell barcode. For each cell’s MHC I and MHC II reads, we searched for unique sequences ~90 nt in length that appeared in at least 8% of reads. These unique sequences were considered the MHC alleles for the donor from which the cell originated. Cells with identical MHC alleles were considered to come from the same individual.

### Identification of influenza A minimal peptide-specific memory B cells

We performed two rounds of sorting for memory B cells that bound the influenza A minimal peptide VPNGTLVKTITNDQI, the first using 11 donor blood collars and the second using a new set of 10 donor blood collars. During the first round, we obtained 30 paired heavy and light chain sequences and chose seven to clone and recombinantly express, including four that featured similar gene segment usage. Using dot blot and VirScan with the saturating mutagenesis public epitope library, we validated that only these four antibodies: flu_c326, flu_c357, flu_c504, and flu_c645, originating from three different donors, were specific for the VPNGTLVKTITNDQI peptide (Fig. 3.4B, fig. S3.6A). These four antibodies exhibited highly conserved gene segment usage: they all featured IgHV5-51 in the heavy chain and IgKV4-1 and IgKJ2 in the light chain. These antibodies also shared highly similar light chain CDR3s, but very poorly conserved heavy chain CDR3 sequences. During the second round of the experiment, we obtained 71 paired heavy and light chain sequences and chose 12 of these to clone and recombinantly express, including three that contained the IgHV5-51 / IgKV4-1 / IgKJ2 gene segment combination, as well as all antibodies with either IgHV5-51 or IgKV4-1, but not the combination of both. Using dot blot VirScan with the saturating mutagenesis public epitope library, we validated that only the three antibodies with the IgHV5-51 / IgKV4-1 / IgKJ2 combination: flu_c3, flu_c286, and flu_c473, originating from three different individuals, were specific for VPNGTLVKTITNDQI (Fig. 3.4B, fig. S3.6C). None of the antibodies with only IgHV5-51 or only IgKV4-1 bound the influenza A minimal peptide. Of the 101 BCR sequences obtained from VPNGTLVKTITNDQI-sorted memory B cells from the two sorting experiments, the IgHV5-51 / IgKV4-1 / IgKJ2 gene segment combination was only found in the seven antibodies that we ultimately validated as specific for the peptide. The full list of antibodies we tested for specificity for VPNGTLVKTITNDQI is provided in table S3.8.

### Identification of additional influenza A minimal peptide-specific BCRs from a dataset of BCRs from an individual pre- and post-influenza vaccination

To investigate whether we could identify additional BCRs specific for the influenza A minimal peptide VPNGTLVKTITNDQI by simply selecting for the conserved V gene segments and light chain CDR3 characteristics of known binders,we searched a dataset of ~100,000 BCR sequences from an individual pre- and post-influenza vaccination (3.61, 3.62) for those with IgHV5-51, IgKV4-1, IgKJ2, and a light chain CDR3 similar to our example set. We identified five such class-switched BCRs and found that two (flu_c2760 and flu_c4582) bound the influenza A minimal peptide and exhibited similar high-resolution footprints to those of the peptide tetramer-sorted BCRs (Fig. 3.4B, fig. S3.6B, fig. S3.7; table S3.8). Flu_c2760 and flu_c4582were the only BCRs of the set of five with long heavy chain CDR3s (23 and 22 AA, respectively). Flu_c2760 was flu-responsive, expanding in the post-influenza vaccination repertoire (3.62).

### Identification of EBV minimal peptide-specific memory B cells

We also performed two rounds of sorting for the EBV minimal peptide PPSTSSKLRPRWTFT, the first using 11 donor blood collars and the second using a new set of 11 donor blood collars. During the first round, we obtained 54 paired heavy and light chain sequences and chose 6 to clone and recombinantly express. Using dot blot and VirScan with the saturating mutagenesis public epitope library, we validated that one antibody, EBV_c186, was specific for the PPSTSSKLRPRWTFT peptide (Fig. 3.4C and fig. S3.6A). During the second round of sorting, we obtained 94 paired heavy and light chain sequences. We initially chose 10 of these to clone and recombinantly express, including six antibodies from three different donors that featured the same V gene segment usage as EBV_c186, namely, IgHV1-46 / IgLV3-10. These six antibodies: EBV_c9, EBV_c19, EBV_c61, EBV_c149, EBV_c101, and EBV_c150, validated as specific for PPSTSSKLRPRWTFT, as well as three other antibodies: EBV_c40, which featured a IgHV1-46 / IgLV3-1 gene segment combination, and EBV_c3 and EBV_c120, which were from the same B cell precursor and donor and featured a IgHV1-8 / IgLV1-51 combination (Fig. 3.4C, fig. S3.6D). Because most of the antibodies from this initial selection validated as specific for the PPSTSSKLRPRWTFT peptide, we selected 23 additional antibodies from the same dataset to clone and recombinantly express, including every remaining antibody that shared the IgHV1-46 / IgLV3-1 or IgHV1-8 / IgLV1-51 combinations, several antibodies with either IgHV1-46 or IgLV3-10 but not both, and several antibodies chosen at random. Using dot blot and VirScan with the saturating mutagenesis public epitope library, we validated that nine of these 23 antibodies were specific for the PPSTSSKLRPRWTFT peptide: three antibodies: EBV_c63, EBV_c83, and EBV_c124, which featured IgHV1-46; four antibodies: EBV_c10, EBV_c77, EBV_c127, and EBV_c138, which were from the same B cell precursor and donor as EBV_c3 and EBV_c120and featured the IgHV1-8 / IgLV1-51 combination; one antibody, EBV_c57, which featured a IgHV1-8 / IgLV1-47 combination; and one additional antibody: EBV_c98, which featured a IgHV3-30 / IgKV2-30 combination (Fig. 3.4C, fig. S3.6E). Of all the cells we sorted and sequenced for the EBV minimal peptide, the IgHV1-46 / IgLV3-10 combination was only found among the antibodies we ultimately validated as specific for the peptide and one other antibody, EBV_c626, which exhibited weak binding to the peptide by dot blot (fig. S3.6F). The full list of antibodies we tested for specificity for the PPSTSSKLRPRWTFT is provided in table S3.9.

### Recombinant expression of antibodies

Heavy and light chain sequences of BCRs of interest were synthesized as gene fragments (IDT, Gene Universal) and cloned into human IgG, IgK, and IgL expression vectors (Human IgG Vector Set, Sigma) according to the manufacturer’s instructions. Recombinant expression of antibodies was performed as previously described using the Expi293 Expression System Kit (Thermo Fisher Scientific) (3.63) or the ExpiCHO Expression System (Thermo Fisher Scientific). Filtered cell culture supernatant was used for dot blot and VirScan characterization. Antibody purification was performed using NAb Protein A Plus Spin Column (Thermo Fisher Scientific) according to the manufacturer’s instructions.

### Dot blot

Nitrocellulose membrane was marked with pencil to indicate the regions where the peptides would be spotted. Four micrograms of recombinant peptide was spotted onto each marked region of the nitrocellulose membrane, then the membrane was cut into strips and blocked in Tris Buffered Saline with Tween 20 (Cell Signaling Technology) and 5% bovine serum albumin (TBST 5% BSA) for 30 min at room temperature. The membrane was next incubated in cell culture supernatant from recombinant antibody expression diluted 1:20 in TBST 5% BSA for 1 hour at room temperature. Nitrocellulose was washed three times with TBST for 5 min per wash, then incubated with anti-human IgG-HRP (Southern Biotech) diluted 1:500 in TBST 5% BSA for 1 hour at room temperature. Nitrocellulose was washed three times with TBST for 5min per wash, then once with TBS for 5 min. Nitrocellulose was incubated with SuperSignal West Femto Maximum Sensitivity Substrate enhanced chemiluminescent substrate (Thermo Fisher Scientific) according to the manufacturer’s instructions and chemiluminescent signals were captured using a CCD camera-based imager.

### Influenza A hemagglutinin ELISA

Expression and purification of hemagglutinin (HA) trimers and antibodies: Cloning, expression, and purification of HA ectodomain trimers were carried out as previously described (3.64). Briefly, HAs were expressed as soluble trimers with a C-term foldon trimerization domain and a 6x-His tag (HA1-HA2-glyserthrombin-glyser-foldon-glyser-HHHHHH) (3.65). HA1-HA2 sequences (residue 1-504 H3 numbering) from A/Aichi/02/1968 Aichi (H3), A/ Shanghai/1/2013 SH13 (H7), A/Jiangxi-Donghu/346/2013 JX346 (H10), A/swine/HuBei/06/2009 HB09 (H4N1), A/California/04/2009 CA09 (H1N1), A/Vietnam/1203/2004 Viet04 (H5N1), A/Japan/305/1957 JP57 (H2N2) and A/guinea fowl/Hong Kong/1999 WF10 (H9N2) were cloned into a pTT5 expression vector containing the C-term tags. Soluble HA trimers were expressed by transient transfection in the Expi293 Expression System and purified from supernatants by Ni-NTA chromatography followed by size exclusion chromatography (SEC). Purified soluble HA trimers were stored in TBS buffer (20 mM Tris pH 8.0, 150 mM NaCl and 0.02% sodium azide) at 4°C and used for at least one month. FI6 (3.65) antibody was produced as described for hemagglutinin trimers. Briefly, antibody genes were cloned into the pTT5 expression vector, expressed by transient transfection in Expi293T cells, and purified from cell supernatants using Ni-NTA chromatography followed by SEC. Proteins were stored in TBS buffer at 4°C and used for up to 6 months.

Influenza A hemagglutinin ELISA:

The binding of the recombinant IgG influenza A minimal peptide-specific antibodies to hemagglutinin ectodomain trimers was measured by ELISA as described previously (3.64). Briefly 384-well plates (Sigma) were coated with 10 mg/ml of recombinant HAs in 0.1 M NaHC03 pH 9.8 and stored overnight at 4°C. Plates were then blocked with 3% BSA in TBS-T (TBS with 0.1% Tween 20) for 1 hour at room temperature. Plates were washed with TBS-T after each step. Antibodies were diluted to 10 mg/ml with TBS-T and serially diluted fourfold for a total of eight dilutions and added for 3 hours at room temperature. Secondary HRP-conjugated goat anti-human IgG (Southern Biotech) diluted 1:10,000 was added for 1 hour at room temperature. The SuperSignal ELISA Femto Maximum Sensitivity Substrate (Thermo Fisher Scientific) was used to develop the plates, and the plates were read at 425nm. ELISA binding curves were plotted using Graphpad Prism 8.3.

### In vitro influenza A neutralization assay

Neutralization assays were conducted as described previously (3.64). Briefly, live PB1flankeGFP virus were used for BSL 2 strains A/Aichi/02/1968 (X31; H3N2), A/California/04/2009 (CA09; H1N1) (3.66), using reagents kindly provided by Dr. J. Bloom (Fred Hutchinson). MDCK cells were used for live neutralization assays. Purified antibodies were diluted to 50 mg/ml except for flu_c3 and flu_c286, which were at 30 mg/ml and 6.7 mg/ml, respectively, and then serially diluted fivefold for a total of eight dilutions. Pseudovirus assays (3.67) were conducted for BSL 3 strains A/Shanghai/1/2013 (SH13; H7), A/Jiangxi-Donghu/346/2013 (JX346;H10) and A/Vietnam/1203/2004 (Viet04; H5). TZM-bl cells were used for the pseudoviral neutralization assays. Antibodies were diluted to 50 mg/ml except for flu_c3 and flu_c286, which were at 30mg/ml and 6.7 mg/ml, respectively, and then serially diluted fourfold for a total of eight dilutions. Percent neutralization was then determined for each concentration of antibody and plotted using Graphpad Prism 8.3.

### Live cell immunofluorescence

The EBV+ Burkitt lymphoma cell line P3HR-1-ZHT was used for EBV lytic reactivation (3.68). P3HR1-ZHT cells stably express a conditional BZLF1 allele (BZLF1-HT), in which the ligand binding domain of a modified estrogen receptor responsive to 4HT is fused to the BZLF1 C terminus. In the absence of 4HT, BZLF1-HT is retained in the cytosol and is destabilized. 4HT addition stabilizes BZLF1-HT and drives its rapid nuclear translocation which leads to lytic reactivation. P3HR1-ZHT cells (1x10^6^ cells/ml) were reactivated with 4HT (1 mM) for 24 hours. For live cell staining, 1x10^6^ cells were washed twice with live cell staining buffer (PBS with 1 mM EDTA and 0.5% BSA), followed by incubation with primary antibodies at 2 μg/ml for 30 min on ice. Cells were then washed with the staining buffer twice and subsequently stained with Alexa Fluor 488-conjugated anti-human IgG secondary antibody (Jackson Immunoresearch) at 6 μg/ml and Cy5-conjugated anti-gp350 mouse monoclonal antibody 72A1 (gift of Dr. E. Kieff) at 2 μg/ml for 30 min on ice. Labeled cells were washed three more times with FACS buffer and the nucleus was stained by Hoechst 33258 at 1 μg/ml prior to confocal microscopy using the LSM800 system with an Apochromat 63x/1.4 Oil DIC M27 objective lens (Zeiss). Images were analyzed with Adobe Photoshop.

### SARS-CoV-2 spike ELISA

Ninety-six-well maxisorp ELISA plates (Thermo Fisher Scientific) were coated with 2 mg of full-length SARS-CoV-2 (2019-nCoV) spike protein (S, gift of B. Chen, Ragon Institute of MGH, MIT and Harvard) or S2 (purchased from GenScript) in 35 ml of PBS overnight at 4°C. After discarding coating buffer, ELISA plates were blocked with 50 ml of PBS with 3% BSA at room temperature for 2 hours. During the time of incubation, monoclonal antibodies were serially diluted twofold using 1 mg/ml as a starting concentration in 1% BSA prepared in PBS with 0.03% Tween 20. The anti-peanut PT275-H40 monoclonal antibody (gift of Dr. D. R. Wesemann) was used as a negative control. After blocking, the solution was discarded and ELISA plates were washed once in PBS containing 0.05% Tween 20. Monoclonal antibody dilutions were transferred into the plates in duplicates along with standards and incubated overnight at room temperature. Afterward, primary antibody solution was decanted and plates were washed thrice in PBS containing 0.05% Tween 20. Secondary antibody solutions of anti-human IgG alkaline phosphatase (AP) (Southern Biotech) diluted 1:2000 in PBS containing 1% BSA and 0.03% Tween 20were added to each plate at 30 ml per well. Plates were incubated for 90 min at room temperature and then washed thrice. Alkaline phosphatase substrate p nitrophenyl phosphate tablets (Sigma, St. Louis, MO) were dissolved in 0.1 M glycine, pH 10.4, with 1 mM MgCl2 and 1 mM ZnCl2, pH 10.4, to a concentration of 1.6 mg/ml. One hundred microliters of this development/substrate solution was then added to each well in a 96-well plate. Plates were kept in the dark and allowed to develop for 2 hours prior to reading. Absorbance at 405 nm was measured using a microplate reader (Biotek Synergy H1). All samples were run in duplicate wells.

### In vitro SARS-CoV-2 neutralization assay

#### Cell culture:

NR-596VeroE6 cells (BEI Resources) were maintained in Dulbecco’s modified Eagle medium (DMEM) (Gibco^™^) with the following additives: 10% heat inactivated fetal bovine serum (Gibco^™^), GlutaMAX (Gibco^™^), non-essential amino acids (Gibco^™^), and sodium pyruvate (Gibco^™^). The day prior to the assay, 8.0x10^5^ VeroE6 cells were seeded per well of a six-well plate in 2 ml of media.

#### Virus propagation:

Authentic SARS-CoV-2 viruses were propagated as previously described (3.46) from passage 4 SARS-CoV-2 USA-WA1/2020 (3.69).

#### Viral plaque reduction neutralization assay:

All viral infection quantification assays were performed at biosafety level 4 (BSL-4) at the National Emerging Infectious Disease Laboratories (NEIDL). In brief, an Avicel plaque reduction assay was used to quantify plaques as follows: Antibody samples were serially diluted in Dulbecco’s Phosphate Buffered Saline (DPBS) (Gibco^™^) using half-log or threefold dilutions. Each dilution was incubated at 37°C and 5% CO2 for 1 hour with 1000 plaque forming units/ml (PFU/ml) of SARS-CoV-2 (isolate USA-WA1/2020 – described above). The maintenance media was then removed from each plate and 200 ml of each inoculum dilution was added to confluent monolayers of NR-596 Vero E6 cells (including 1000 PFU/ml SARS-CoV-2 incubated with DPBS as a positive control and a mock DPBS negative control) in triplicate and incubated for 1 hour at 37°C/5% CO2 with gentle rocking every 10–15 min to prevent monolayer drying. The overlay was prepared by mixing by inversion Avicel 591 overlay (DuPont Nutrition & Biosciences, Wilmington, DE) and 2X Modified Eagle Medium (Temin’s modification, Gibco^™^) supplemented with 2X antibiotic-antimycotic (Gibco^™^), 2X GlutaMAX (Gibco^™^) and 10% fetal bovine serum (Gibco^™^) in a 1:1 ratio. After 1 hour, 2 ml of overlay was added to each well and the plates were incubated for 48 hours at 37°C/5% CO2. Six-well plates were then fixed using 10% neutral buffered formalin prior to removal from BSL-4 space. The fixed plates were then stained with 0.2% aqueous Gentian Violet (RICCA Chemicals, Arlington, TX) in 10% neutral buffered formalin for 30 min, followed by rinsing and plaque counting. The half maximal inhibitory concentrations (IC50) were calculated using GraphPad Prism 8.

### PDB analysis to identify GRAB motifs

Nucleotide sequences of human and mouse V gene segments were obtained from IMGT V-QUEST (3.49, 3.50) and translated to AA sequences. The mouse V gene segment sequences were from multiple strains of mice. We used the RCSB PDB Search API (Number of Distinct Protein Entities ≥3, Sequence = the AA sequence of the relevant V gene segment with an identity cutoff of 93%) to identify relevant structures of Ab–Ag complexes involving the V gene segment. The following json query was used:

“query”: {

“type”: “group”,

“logical_operator”: “and”,

“nodes”: [

{

“type”: “group”,

“logical_operator”: “and”,

“nodes”: [

{

“type”: “terminal”,

“service”: “text”,

“parameters”: {

“operator”: “greater_or_equal”,

“negation”: False,

“value”: 3,

“attribute”: “rcsb_entry_info.polymer_entity_

count_protein”

}

}

]

},

{

“type”: “terminal”,

“service”: “sequence”,

“parameters”: {

“evalue_cutoff”: 0.1,

“identity_cutoff”: 0.93,

“target”: “pdb_protein_sequence”,

“value”: seq

}

},

]

}

Next, for each PDB structure, we loaded the Chothia numbered version of the PDB file from SAbDab (3.70, 3.71) into UCSF Chimera (3.72) and used the command findclash (with parameters VDW overlap −1, hbond 0.0) to detect residues in the antigen chain that interacted with the relevant antibody heavy or light chain. For each PDB structure, we generated a rowby-row summary of each antigen residue (in column“Antigen_Residue”) that interacted with the relevant antibody chain, the antibody residues it interacted with (in column “Interacts_With”), and the subset of those antibody residues whose side chain atoms interacted with the antigen AA (in column “Interacts_With_SC”). Next, using SAbPred ANARCI (3.73, 3.74), we annotated the subset of the residues in the column “Interacts_With_SC” thatwere germline encoded (in column “Interacts_With_Germline_SC”). We also included counts of the number of interacting residues (in columns “Num_Interactions”, “Num_SC_Interactions”, “Num_Germline_SC_Interactions”). As a quality control, we entered the relevant antibody heavy or light sequence into IgBlast and annotated the top hit, % alignment, and Evalue (in columns “IgBlast_TopHit”, “Alignment”, and “Evalue”, respectively), and performed a sequence match between the top hit and the original V gene segment query name (in column “Check”) in order to confirm the identity of the V gene segment. We also annotated whether the antigen was a protein or peptide in the column “Antigen_Type”. Then, we collected the output for all the structures with the relevant V gene segment into a file (“_antigenSummary.csv”). We performed a similar analysis for all the antibody residues that interacted with the antigen and collected the output into a file (“_antibody-Summary.csv”). These files are available on the Harvard Dataverse, doi: 10.7910/DVN/WZCLMB; 10.7910/DVN/DXWJ2Y.

Finally, we searched each “_antigen.csv” V gene segment file for recurrent interactions found in multiple unique Ab-Ag structures between certain germline-encoded residues in the antibody and a given AA (or a small set of biochemically similar AAs) in the antigen. These recurrent interactions were considered GRAB motif interactions. V gene segments for which there was a single example of an Ab-Ag interaction that strongly resembled a GRAB motif interaction present in another V gene segment were also considered to harbor GRAB motifs.

### AlphaFold2 predictions

BCR sequences containing IgLV3-16, IgLV3-27, IgLV1-47, and IgHV3-11*06 were identified from our single-cell sequencing datasets. If residues of the predicted GRAB motifs were somatically hypermutated, we reverted them to the germline-encoded residues at those positions. We could not find BCRs containing IgLV3-22 or IgLV3-9*02 in our datasets, so we substituted these germline V gene segments within the sequence of the BCR containing IgLV3-27. A (GGS)x12 linker was inserted between the heavy variable and light variable region of each BCR sequence, and the structures of these “fusion proteins” predicted using AlphaFold2 (3.33, 3.34).

### Quantification and Statistical Analysis

Statistical details of experiments can be found in the figure legends and materials and methods. Data analysis was performed in Python, R, Graphpad Prism, and Excel.

## Supplementary Material

Table S1

Table S3

Table S2

Table S5

Table S6

Table S7

Table S8

Table S9

Table S10

Table S11

Table S12

Table S13

Table S14

Table S4

Table S16

Table S15

Table S17

18
