# Supplementary material for: Germline-encoded amino acid-binding motifs drive immunodominant public antibody responses
Source: Science. Author manuscript; Available in PMC 2023 Jun 16. (PMC10273302; doi:10.1126/science.adc9498)
Supplement: 18 [file NIHMS1901999-supplement-18.pdf]

Supplemental materials

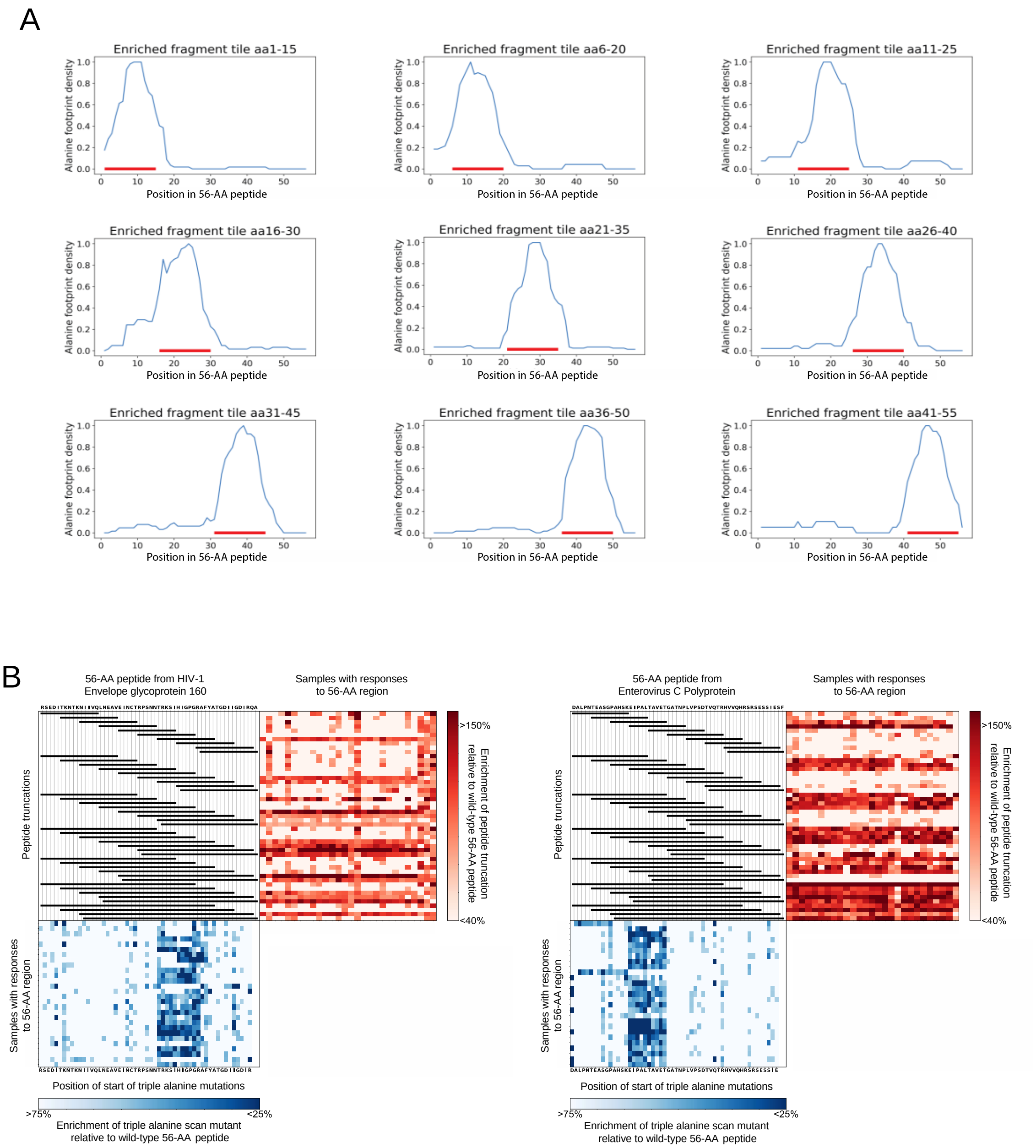

Figure S3.1. Strong concordance observed between position of alanine scan footprints and enriched peptide truncations; locations of epitopes within publicly recognized viral 56-AA peptides are conserved across individuals. (**A**) For each peptide-patient pair, the most strongly enriched 15-AA peptide truncation was identified. Samples were then grouped based on the position of their most enriched 15-AA peptide truncation. To incorporate the alanine scanning data, each plot shows the cumulative density of the alanine scanning footprints among all peptide-patient pairs that most strongly enriched a given 15-AA peptide truncation. For clarity, the position of each 15-AA peptide truncation is plotted in red. (**B**) We performed VirScan using the public epitope truncation and alanine scanning library and the standard Protein A/G IP protocol to map patient antibody responses to the recurrently recognized viral 56-AA peptides. The samples

shown are the subset of individuals with antibody responses to the 56-AA region. The quadrants are as described in Fig. 3.1C. Data are the mean of two technical replicates.

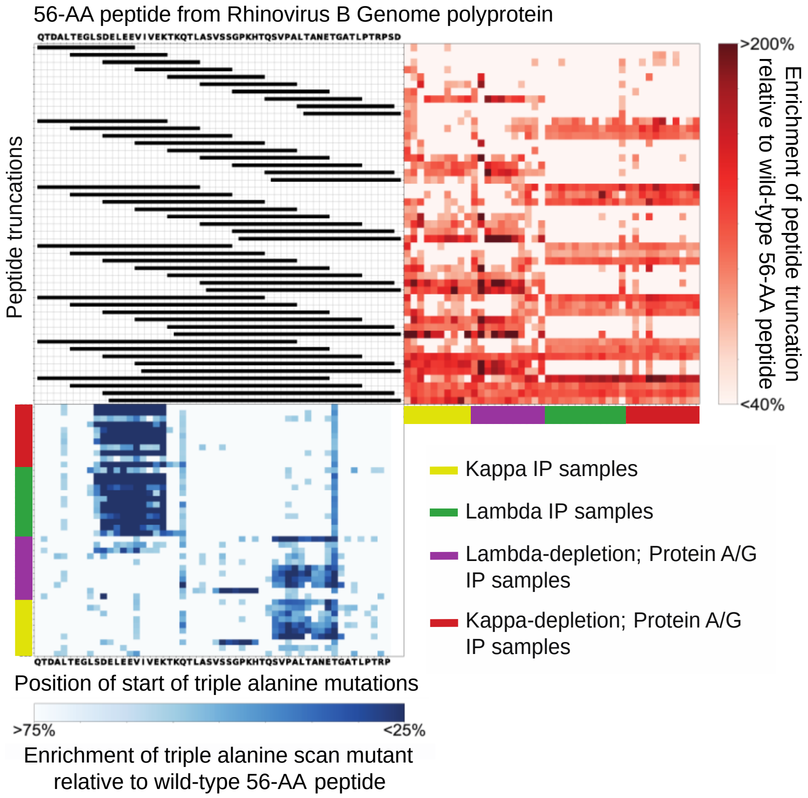

Figure S3.2. Isotype-specific depletions yield results consistent with isotype-specific IPs. To ensure the results in Fig. 3.1C were not artifacts of the isotype-specific IP protocol, we reprofiled antibody responses from several serum samples to the public epitope truncation and alanine scanning library using an alternate approach in which we first depleted kappa or lambda antibodies from the sera samples, then IPed all remaining immunoglobulin using Protein A/G (which binds all IgG antibodies regardless of light chain isotype). This depletion method yielded results consistent with those from the original isotype-specific IPs. Patient antibody responses to a 56-AA region from Rhinovirus B Genome polyprotein are shown. In the heatmaps, the yellow, purple, green, and red colored bars labeled below and to the side of the quadrants indicate the IP or depletion protocol that was used for each sample. The samples shown are the subset of individuals with antibody responses to the 56-AA region. The quadrants are as described in Fig. 3.1C. Data are the mean of two technical replicates.

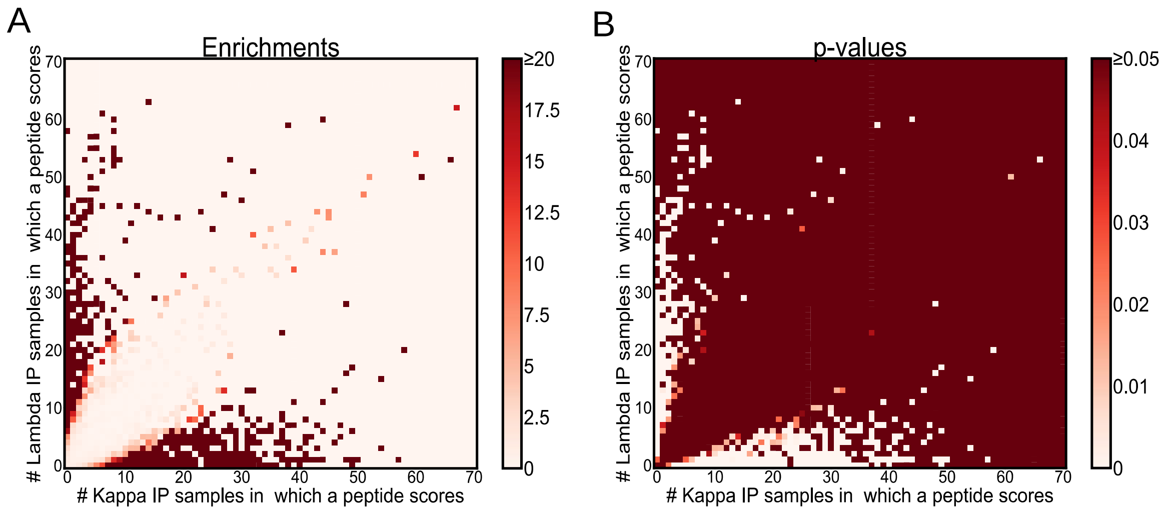

Figure S3.3. Permutation analysis related to Figure 3.1D. (**A**) Enrichments were calculated by dividing the values in Fig. 3.1D, left, by the values in Fig. 3.1D, right. (**B**) P-values were calculated based on how many random permutations resulted in at least such a high number of peptides scoring in the given number of kappa and lambda IP samples (see methods).

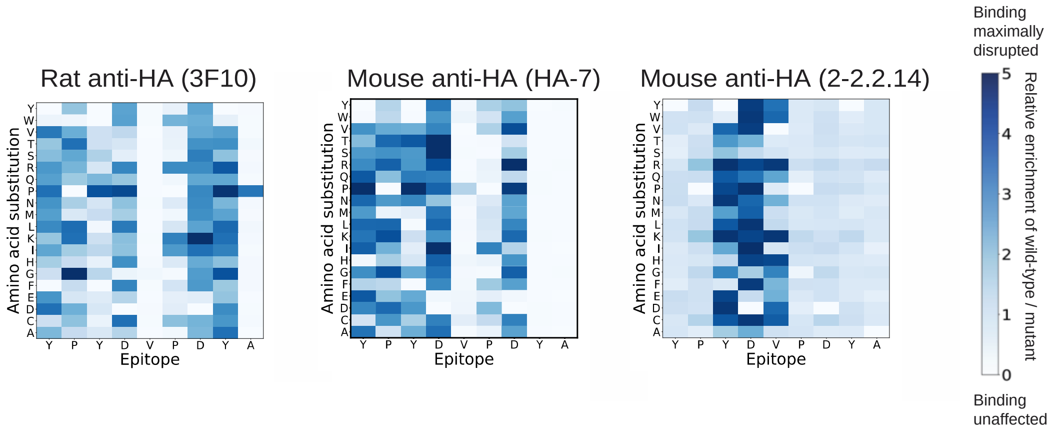

Figure S3.4. High-resolution footprints of HA-tag-specific antibodies. (**A**) High-resolution footprints for commercially available monoclonal antibodies specific for the HA-tag. The axes for the high-resolution footprints are as described in Fig. 3.2B.

A

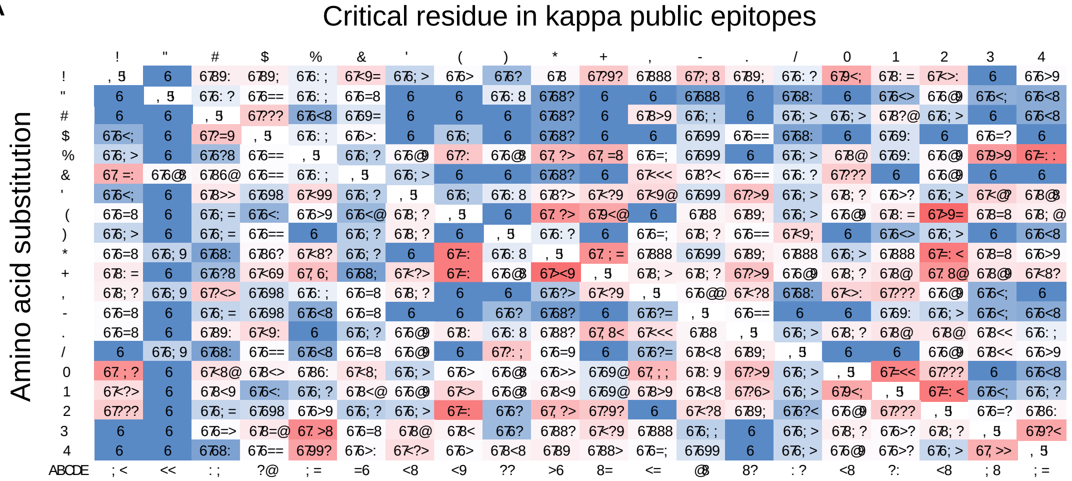

B

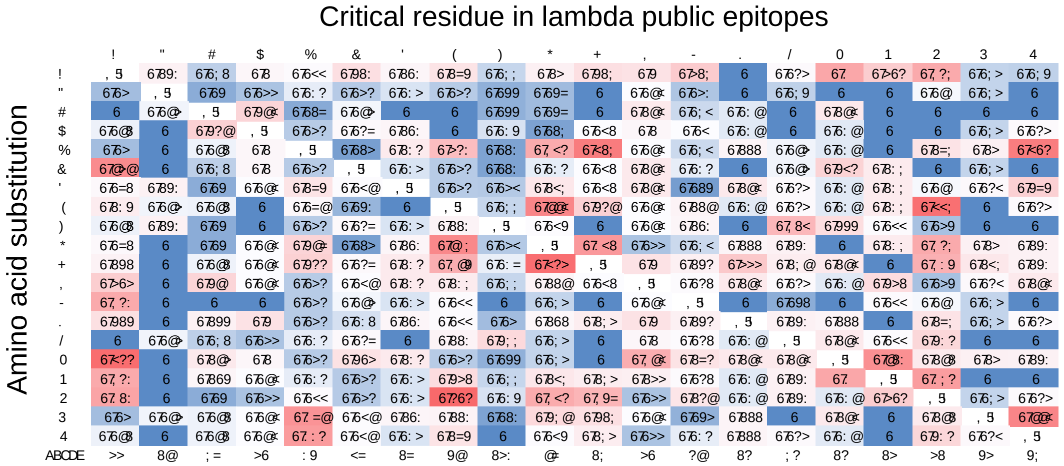

Figure S3.5. Degree to which AA substitutions at critical residues of kappa and lambda public epitopes are tolerated. The heatmaps indicate the frequency with which the AA substitutions indicated by the *y*-axis are permitted at critical residues of kappa (**A**) and lambda (**B**) public epitopes (*x*-axis). Higher values indicate higher degrees of tolerance for the AA substitution. The counts of each AA in critical residues of kappa and lambda public epitopes are at the bottom row of each table.

A

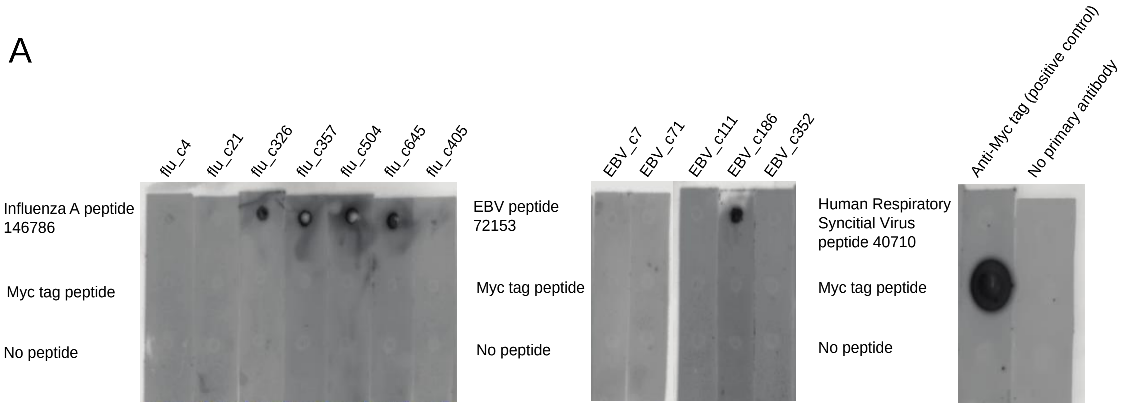
flu_c4
flu_c21
flu_c326
flu_c357
flu_c504
flu_c645
flu_c405
Influenza A peptide 146786
Myc tag peptide
No peptide
EBV_c7
EBV_c71
EBV_c111
EBV_c186
EBV_c352
EBV peptide 72153
Myc tag peptide
No peptide
Anti-Myc tag (positive control)
No primary antibody
Human Respiratory Syncitial Virus peptide 40710
Myc tag peptide
No peptide

B

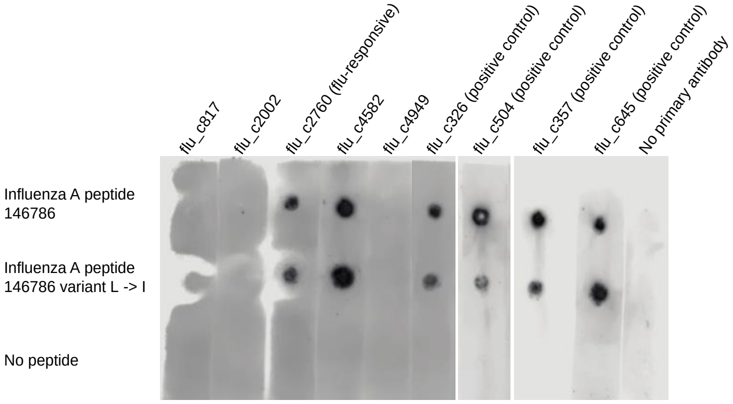
flu_c817
flu_c2002
flu_c2760 (flu-responsive)
flu_c4582
flu_c4949
flu_c326 (positive control)
flu_c504 (positive control)
flu_c357 (positive control)
flu_c645 (positive control)
No primary antibody
Influenza A peptide 146786
Influenza A peptide 146786 variant L -> I
No peptide

C

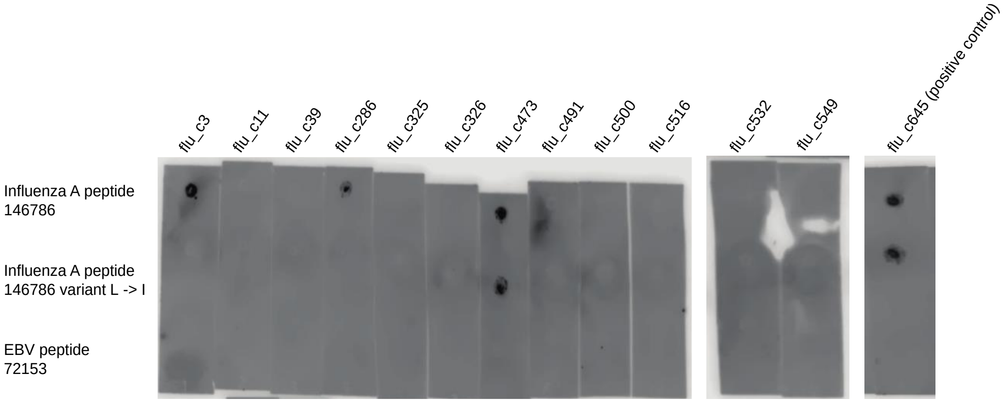
flu_c3
flu_c11
flu_c39
flu_c286
flu_c325
flu_c326
flu_c473
flu_c491
flu_c500
flu_c516
flu_c532
flu_c549
flu_c645 (positive control)
Influenza A peptide 146786
Influenza A peptide 146786 variant L -> I
EBV peptide 72153

Figure S3.6 continued

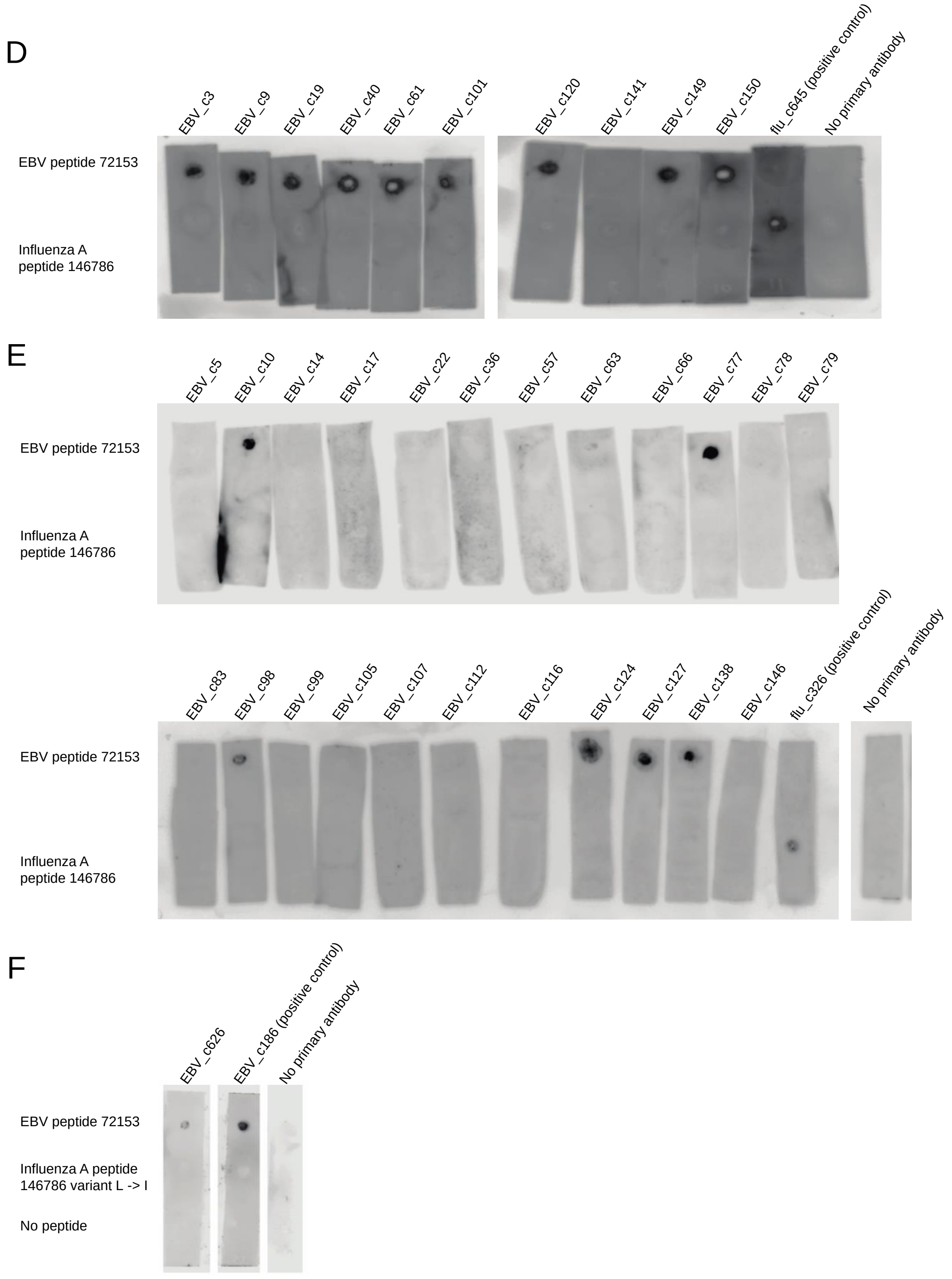

Figure S3.6. Dot blots to test specificity of antibodies for influenza A and EBV minimal peptides. Dot blots to test specificity of antibodies sorted for influenza A minimal peptide VPNGTLVKTITNDQI (peptide 146786 in table S3.3) (**A, C**), antibodies from a patient post-

influenza vaccine that resembled validated influenza A minimal peptide-specific antibodies (**B**), and antibodies sorted for EBV minimal peptide PPSTSSKLRPRWTFT (peptide 72153 in table S3.3) (**A, D, E, F**). The peptides spotted on the nitrocellulose membrane are indicated to the left of each set of dot blots. A Myc tag peptide and anti-Myc tag antibody were used as a positive control for panel (A). In panels B-F, influenza A and EBV minimal peptides were used as controls for each other.

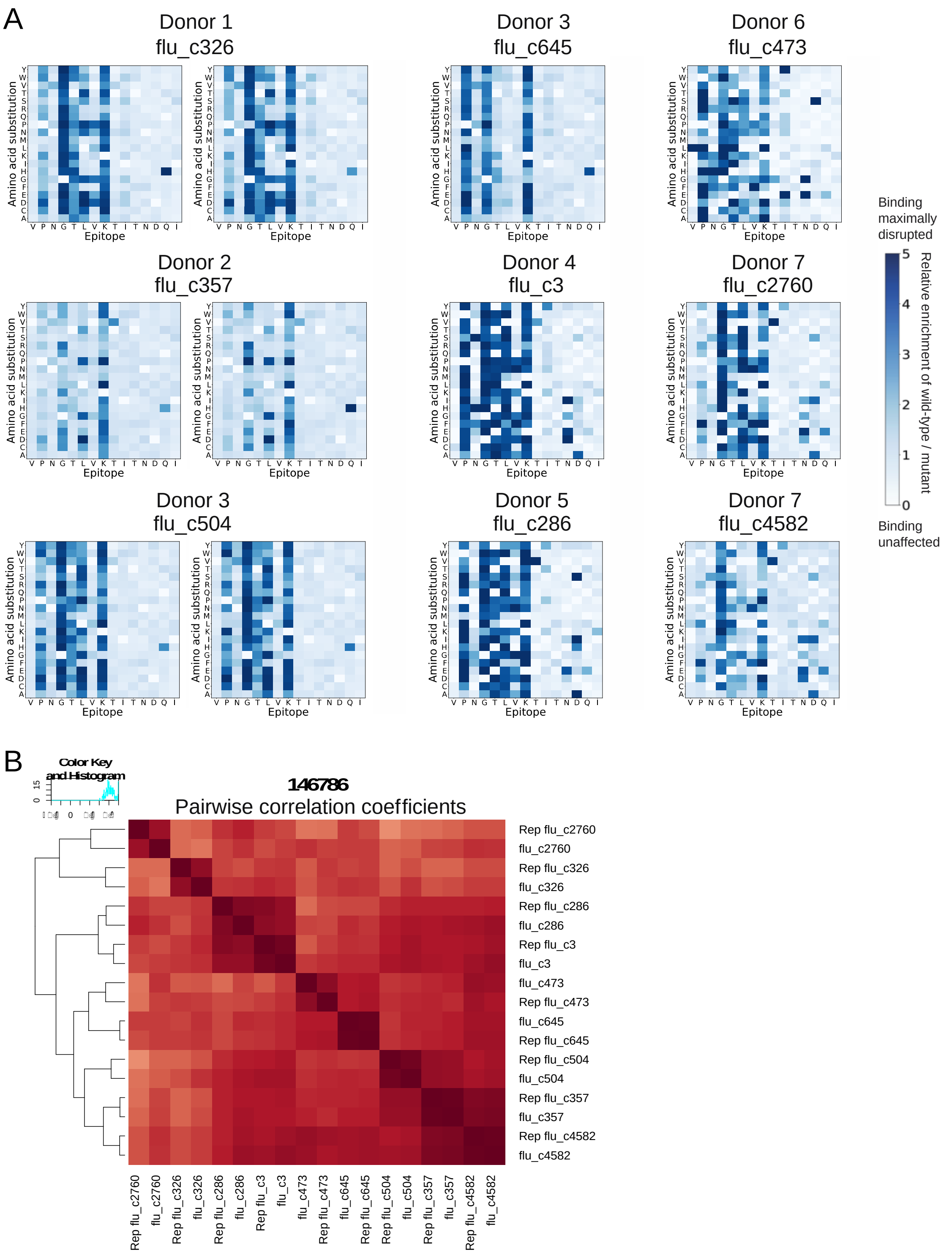

Figure S3.7. High-resolution footprints of influenza A minimal peptide-specific antibodies. (**A**) High-resolution footprints of monoclonal antibodies validated to be specific for the influenza A minimal peptide VPNGTLVKTITNDQI (peptide 146786 in table S3.3). The axes for the high-resolution footprints are as described in Fig. 3.2B. The blood collar donor from which each antibody originated is noted above the footprints. Examples of technical replicates are shown side

by side for flu_c326, flu_c357, and flu_c504. (**B**) We calculated all pairwise Pearson correlation coefficients between the enrichment matrices (see methods) of all of the monoclonal antibodies validated to be specific for the influenza A minimal peptide VPNGTLVKTITNDQI. We plotted these correlations as a clustered heatmap. In the upper left corner of the clustered heatmap is a density distribution of all the correlation coefficient values in the heatmap.

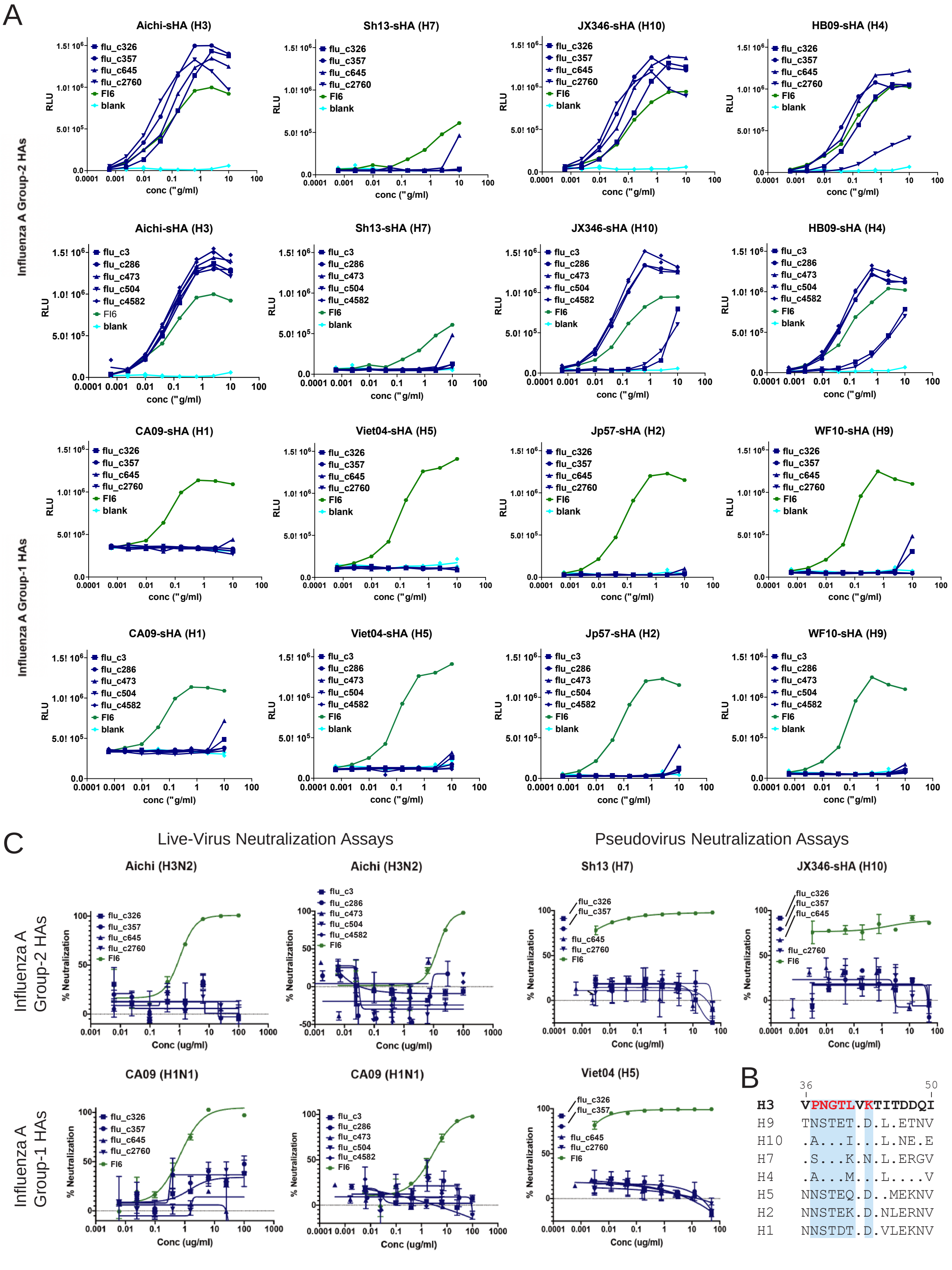

Figure S3.8. Influenza A minimal peptide-specific antibodies bind to HA trimers but are not neutralizing. (**A**) ELISAs were carried out using influenza A minimal peptide-specific antibodies against a panel of recombinant HA ectodomains from both Group 1 and Group 2 influenza A

strains. All antibodies were set at a top concentration of 10 µg/ml and serially diluted 4-fold, and incubated on 384-well plates coated with recombinant influenza A HAs from the following strains: A/Aichi/02/1968 X31 (H3N2), A/Shanghai/1/2013 SH13 (H7N9), A/Jiangxi-Donghu/346/2013 JX346 (H10N8), A/swine/HuBei/06/2009 HB09 (H4N1), A/California/04/2009 CA09 (H1N1), A/Vietnam/1203/2004 Viet04 (H5N1), A/Japan/305/1957 JP57 (H2N2), A/guinea fowl/Hong Kong/1999 WF10 (H9N2). The FI6 broadly neutralizing antibody was used as a positive control; primary antibody was omitted as a blank condition. (**B**) Alignment of HA sequences from influenza A strains used in (A). The region containing the minimal peptide is shown; the consensus critical residues of the public epitope are in red font. (**C**) Neutralization assays were carried out using influenza A minimal peptide-specific antibodies against a panel of live virus and pseudovirus from both Group 1 and Group 2 influenza A strains. Live virus assays were performed with MDCK cells, and pseudovirus assays were performed with TZM-bl cells. Antibodies were set at different top concentrations, serially diluted 4-fold, and incubated with live virus strains A/Aichi/02/1968 X31 (H3N2) and A/California/04/2009 CA09 (H1N1), as well as pseudovirus strains A/Shanghai/1/2013 SH13 (H7N9), A/Jiangxi-Donghu/346/2013 JX346 (H10N8), and A/Vietnam/1203/2004 Viet04 (H5N1). The FI6 broadly neutralizing antibody was used as a positive control. Some data points have error bars that are too small to be easily visible. Dotted lines indicate zero values.

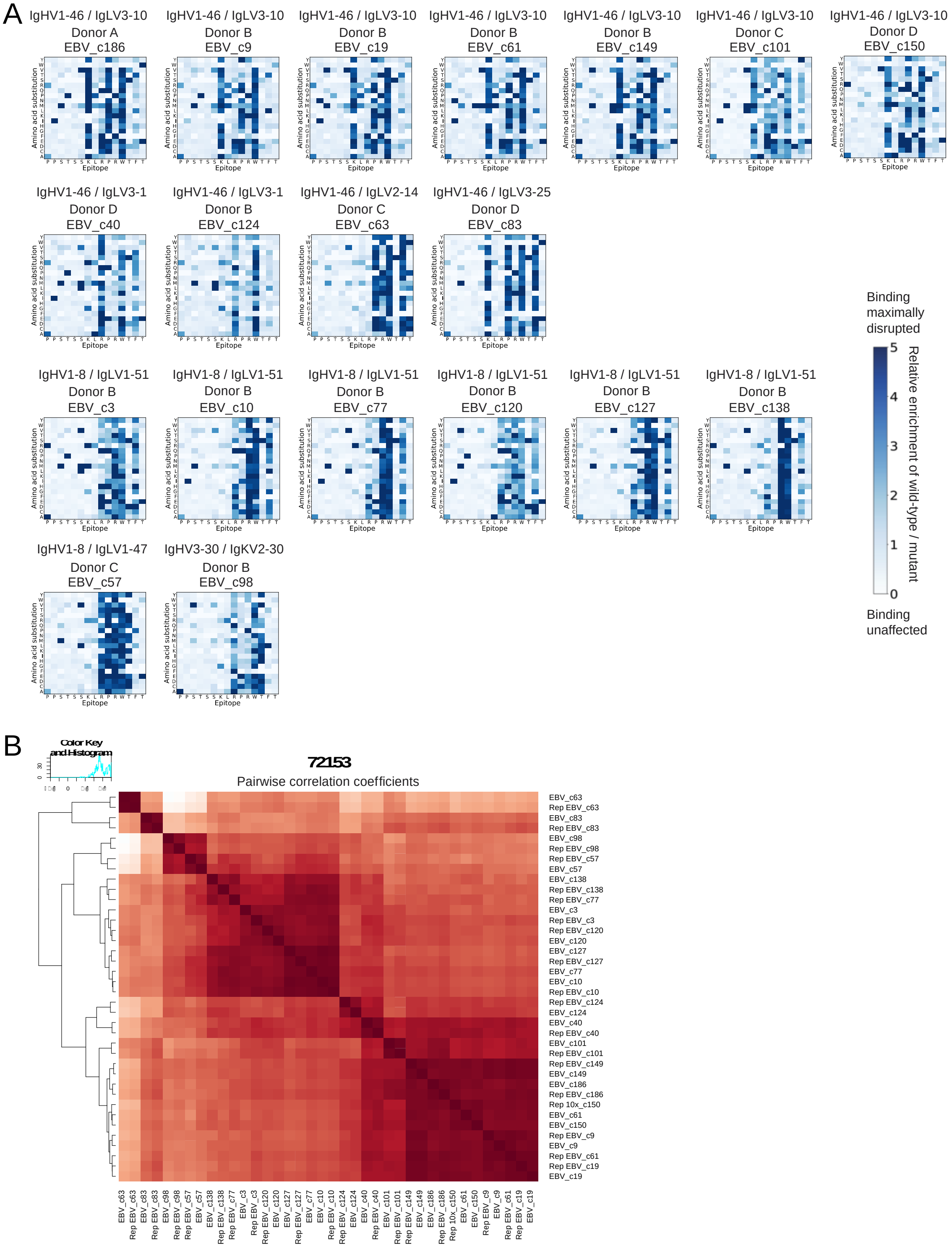

Figure S3.9. High-resolution footprints of EBV minimal peptide-specific monoclonal antibodies. (**A**) High-resolution footprints of all monoclonal antibodies validated to be specific for the EBV minimal peptide PPSTSSKLRPRWTFT (peptide 72153 in table S3.3). The axes for the high-resolution footprints are as described in Fig. 3.2B. Antibody footprints are organized in groups according to their sequence characteristics. For example, all the antibodies in the first row

feature IgHV1-46 and IgLV3-10 gene segment usage and all the antibodies in the second row feature IgHV1-46 paired with other lambda V gene segments. The donor from which each antibody originated is noted above the footprints. (**B**) We calculated all pairwise Pearson correlation coefficients between the enrichment matrices (see methods) of all of the monoclonal antibodies validated to be specific for the EBV minimal peptide PPSTSSKLRPRWTFT. We plotted these correlations as a clustered heatmap. In the upper left corner of the clustered heatmap is a density distribution of all the correlation coefficient values in the heatmap.

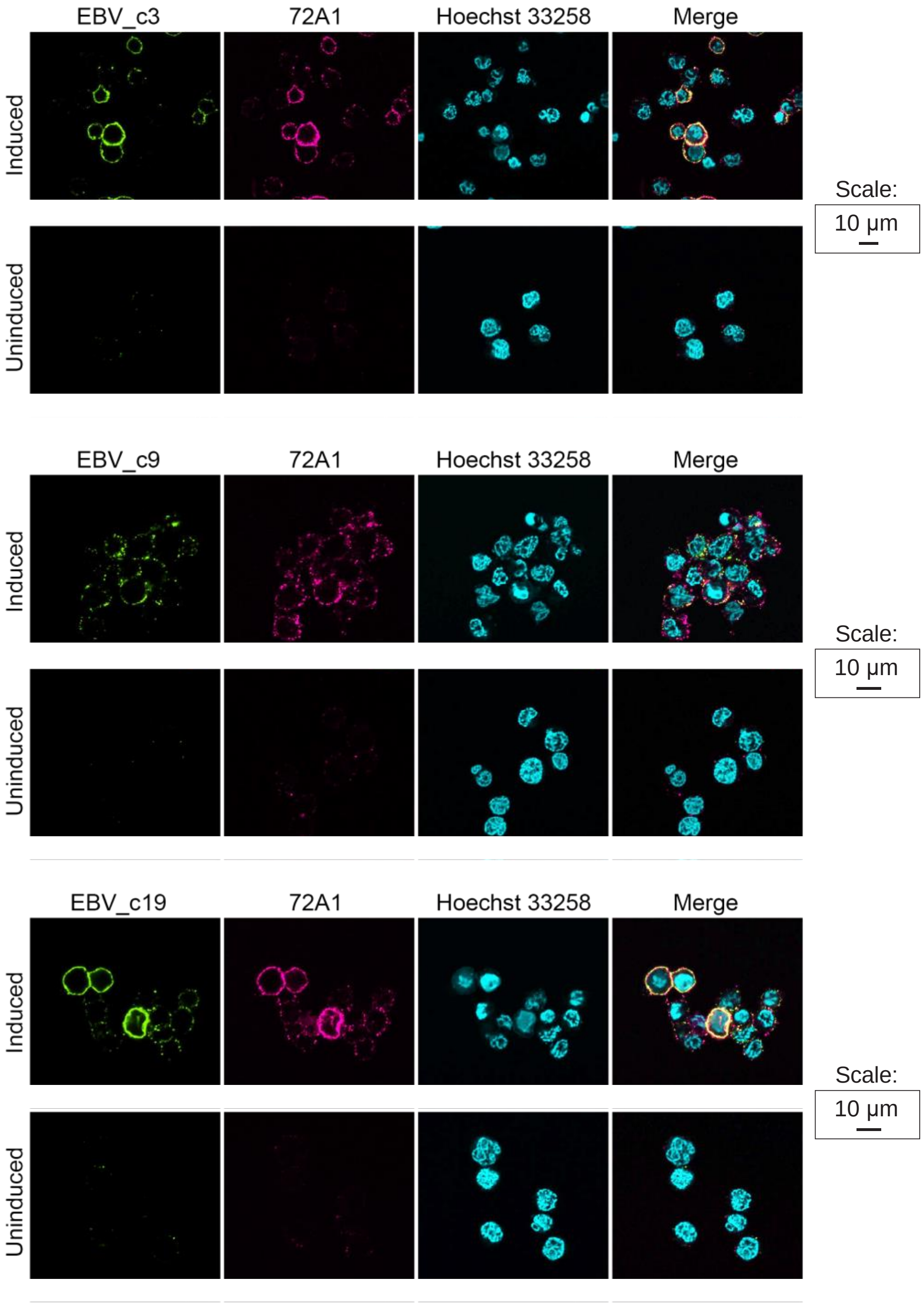
EBV_c3
72A1
Hoechst 33258
Merge
Induced
Uninduced
Scale:
10 µm
EBV_c9
72A1
Hoechst 33258
Merge
Induced
Uninduced
Scale:
10 µm
EBV_c19
72A1
Hoechst 33258
Merge
Induced
Uninduced
Scale:
10 µm

Figure S3.10 continued

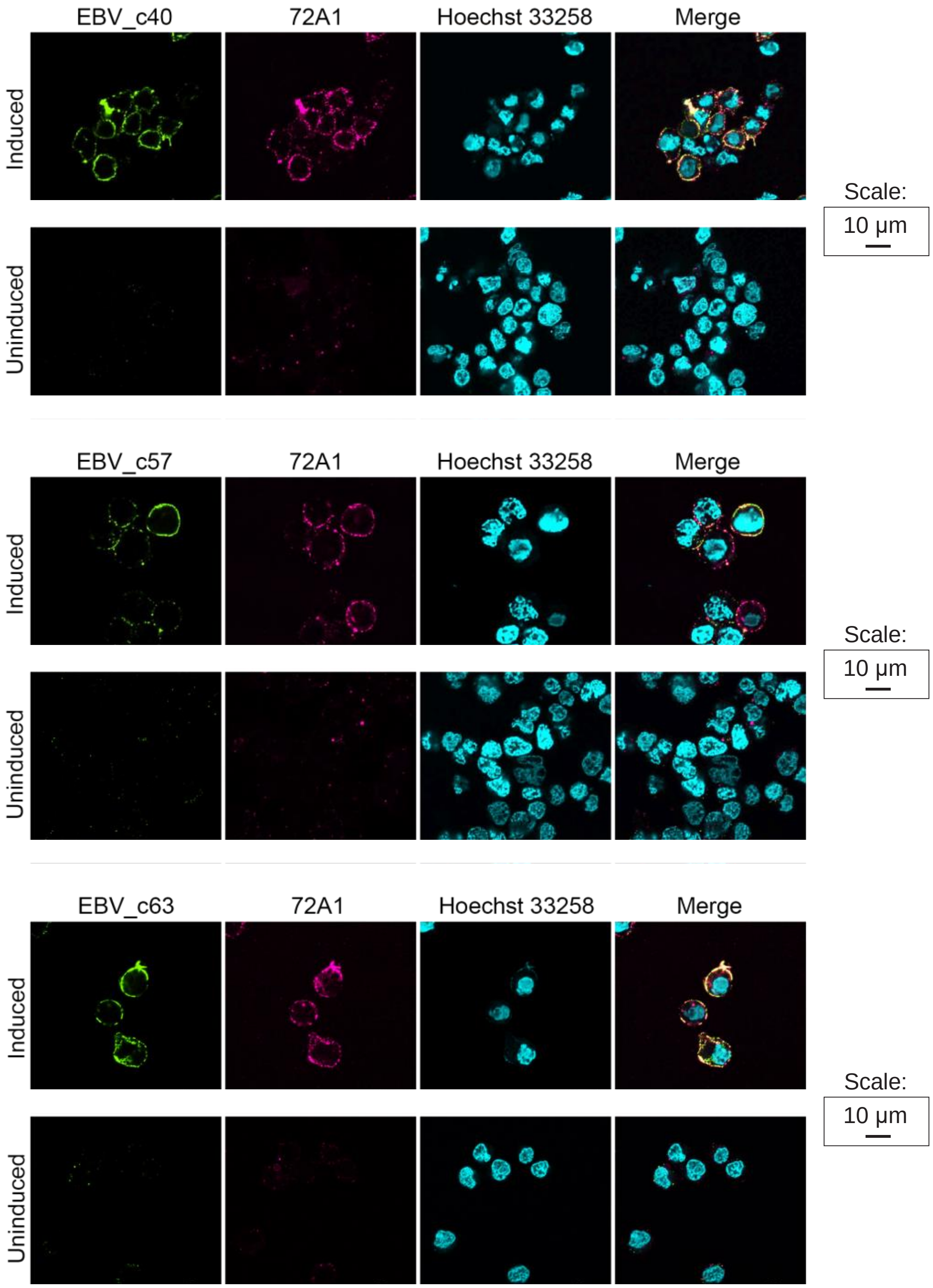

Figure S3.10 continued

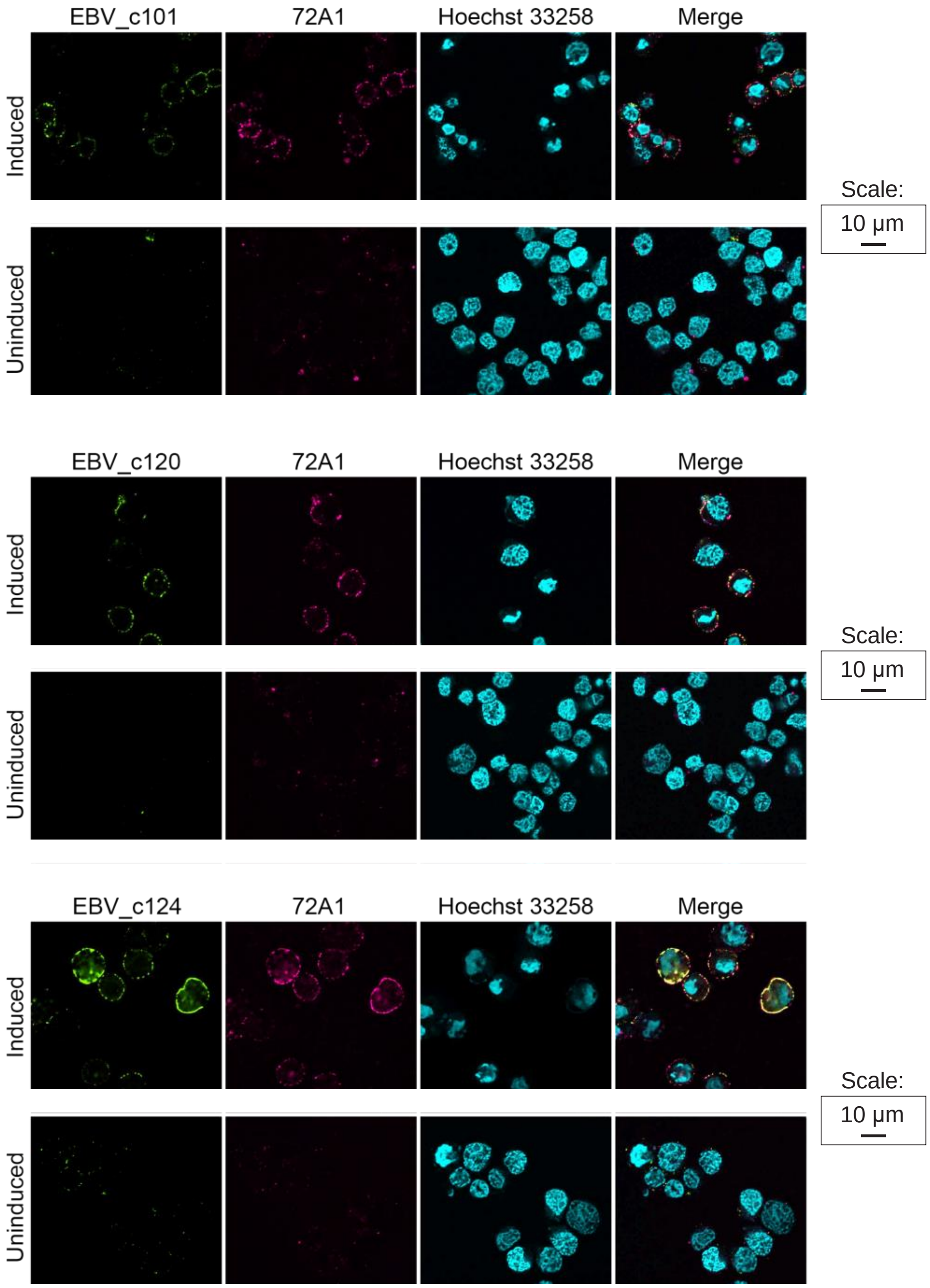

Figure S3.10 continued

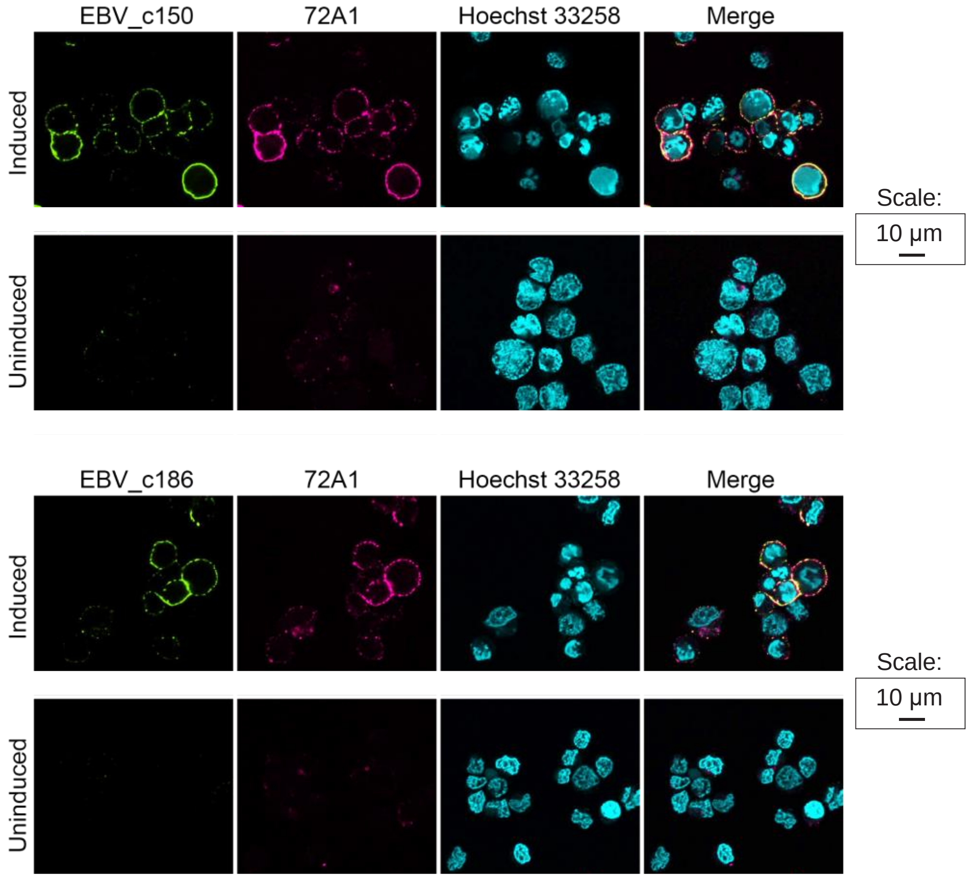

Figure S3.10. EBV minimal peptide-specific antibodies bind full-length gp350. The EBV+ Burkitt lymphoma cell line P3HR-1-ZHT was treated with 4HT to induce lytic EBV reactivation, then the live cells were stained with EBV minimal peptide-specific antibodies. The cells were subsequently stained with Alexa Fluor 488-conjugated anti-human IgG secondary antibody (Jackson Immunoresearch) and Cy5-conjugated anti-gp350 mouse monoclonal antibody 72A1 as a positive control, and finally with Hoechst 33258 to visualize nuclei. Live cells were imaged by confocal microscopy.

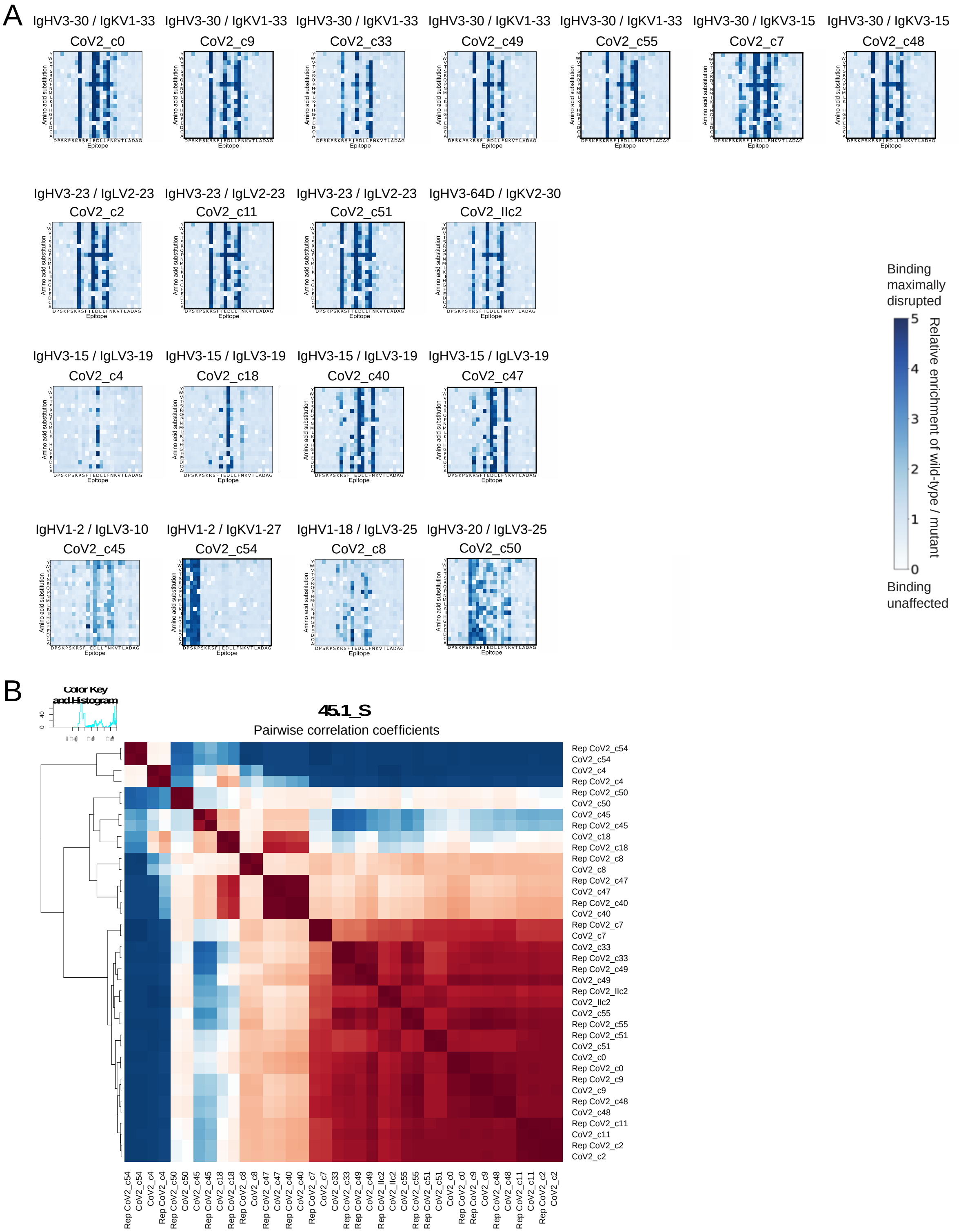

Figure S3.11. High-resolution footprints of SARS-CoV-2 minimal peptide-specific monoclonal antibodies. (**A**) High-resolution footprints of all monoclonal antibodies validated to be specific for the SARS-CoV-2 peptide DPSKPSKRSFIEDLLFNKVTLADAG (peptide 45.1_S in

table S3.12). The axes for the high-resolution footprints are as described in Fig. 3.2B. Antibody footprints are organized in groups according to their sequence characteristics. Analysis of MHC transcripts to identify which donor each BCR originated from was not performed, as the protocol for the Chromium Next GEM Single Cell 5' Kit v2 (10x Genomics) was updated from with the original Chromium 5' V(D)J solution (10x Genomics) protocol and was no longer compatible with our custom MHC analysis. (**B**) We calculated all pairwise Pearson correlation coefficients between the enrichment matrices (see methods) for the monoclonal antibodies validated to be specific for the SARS-CoV-2 peptide DPSKPSKRSFIEDLLFNKVTLADAG. We plotted these correlations as a clustered heatmap. In the upper left corner of the clustered heatmap is a density distribution of all the correlation coefficient values in the heatmap.

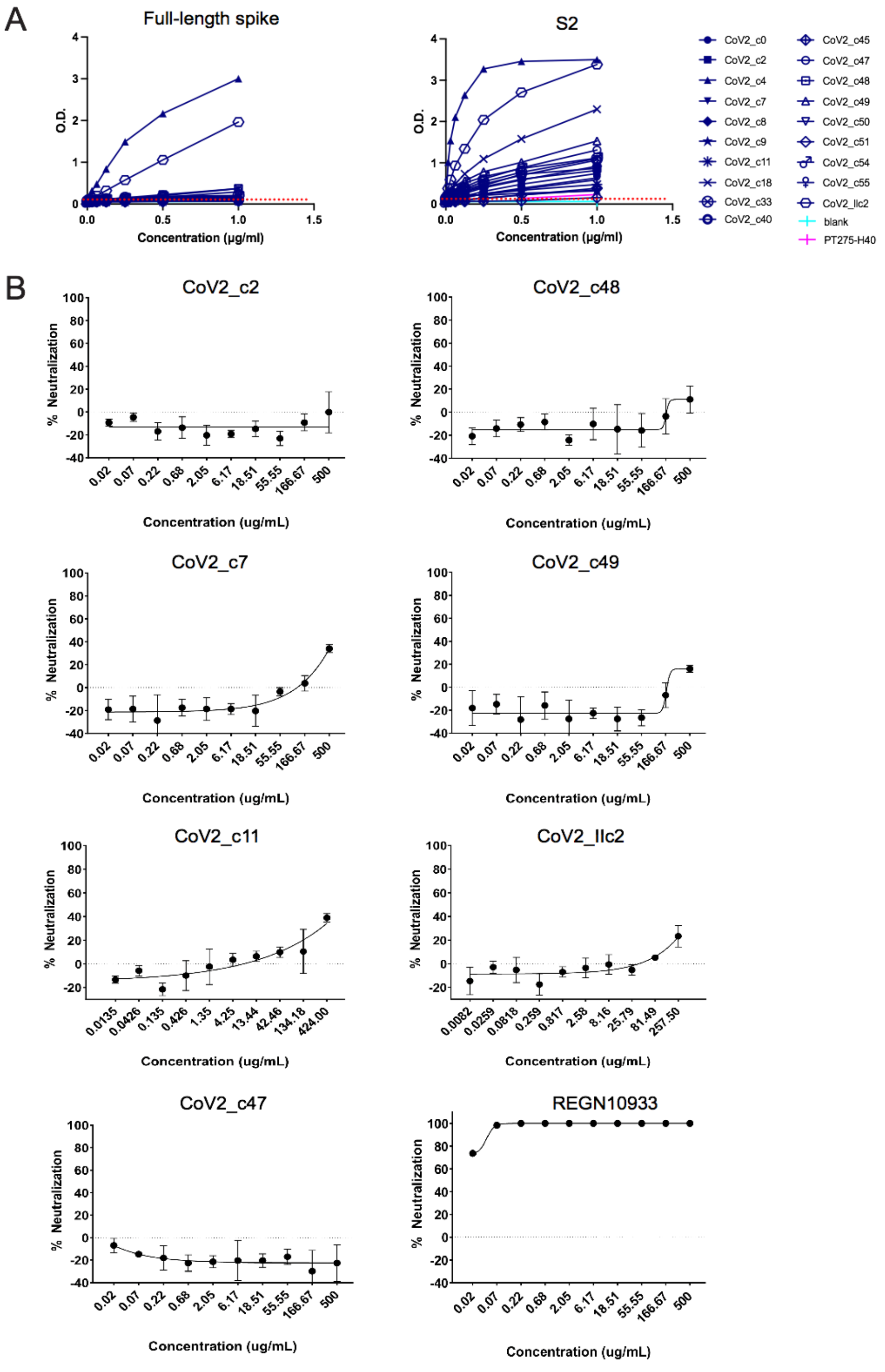

Figure S3.12. SARS-CoV-2 minimal peptide-specific antibodies bind to the S2 domain of spike and are not potently neutralizing. (**A**) ELISAs were carried out using SARS-CoV-2 minimal peptide-specific antibodies. All antibodies were set at a top concentration of 1 μg/ml and serially diluted 2-fold, and incubated on 96-well plates coated with full-length SARS-CoV-2 (2019-

nCoV) spike protein or S2. The anti-peanut (PT275-H40) monoclonal antibody was used as a negative control; primary antibody was omitted as a blank condition. Values are an average of technical replicates. The dotted red lines indicate zero values. (**B**) Neutralization assays were carried out using SARS-CoV-2 minimal peptide-specific monoclonal antibodies against live SARS-CoV-2 virus. Live virus assays were performed with NR-596 VeroE6 cells. Antibodies were set at different top concentrations, serially diluted 3-fold, and incubated with live virus strain SARS-CoV-2 USA-WA1/2020 (3.69). The neutralizing monoclonal antibody REGN10933 (3.75) was used as a positive control. Percent neutralization was determined by quantifying the reduction in viral plaque formation. The dotted gray lines indicate zero values.

A

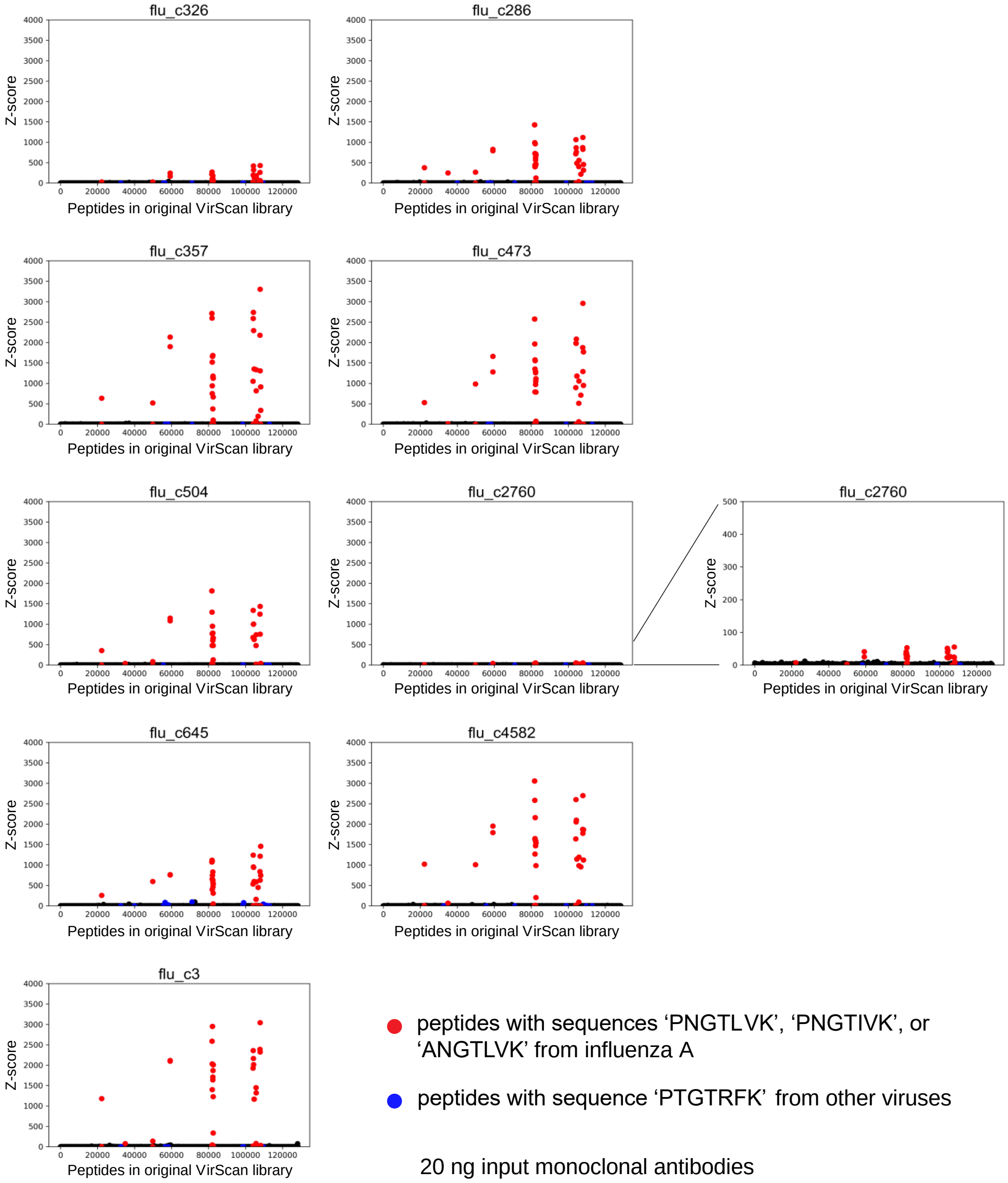
flu_c326
flu_c286
flu_c357
flu_c473
flu_c504
flu_c2760
flu_c2760
flu_c645
flu_c4582
flu_c3
Z-score
Peptides in original VirScan library
0 20000 40000 60000 80000 100000 120000
0 500 1000 1500 2000 2500 3000 3500 4000
0 100 200 300 400 500
peptides with sequences ‘PNGTLVK’, ‘PNGTIVK’, or ‘ANGTLVK’ from influenza A
peptides with sequence ‘PTGTRFK’ from other viruses
20 ng input monoclonal antibodies

Figure S3.13 continued

B

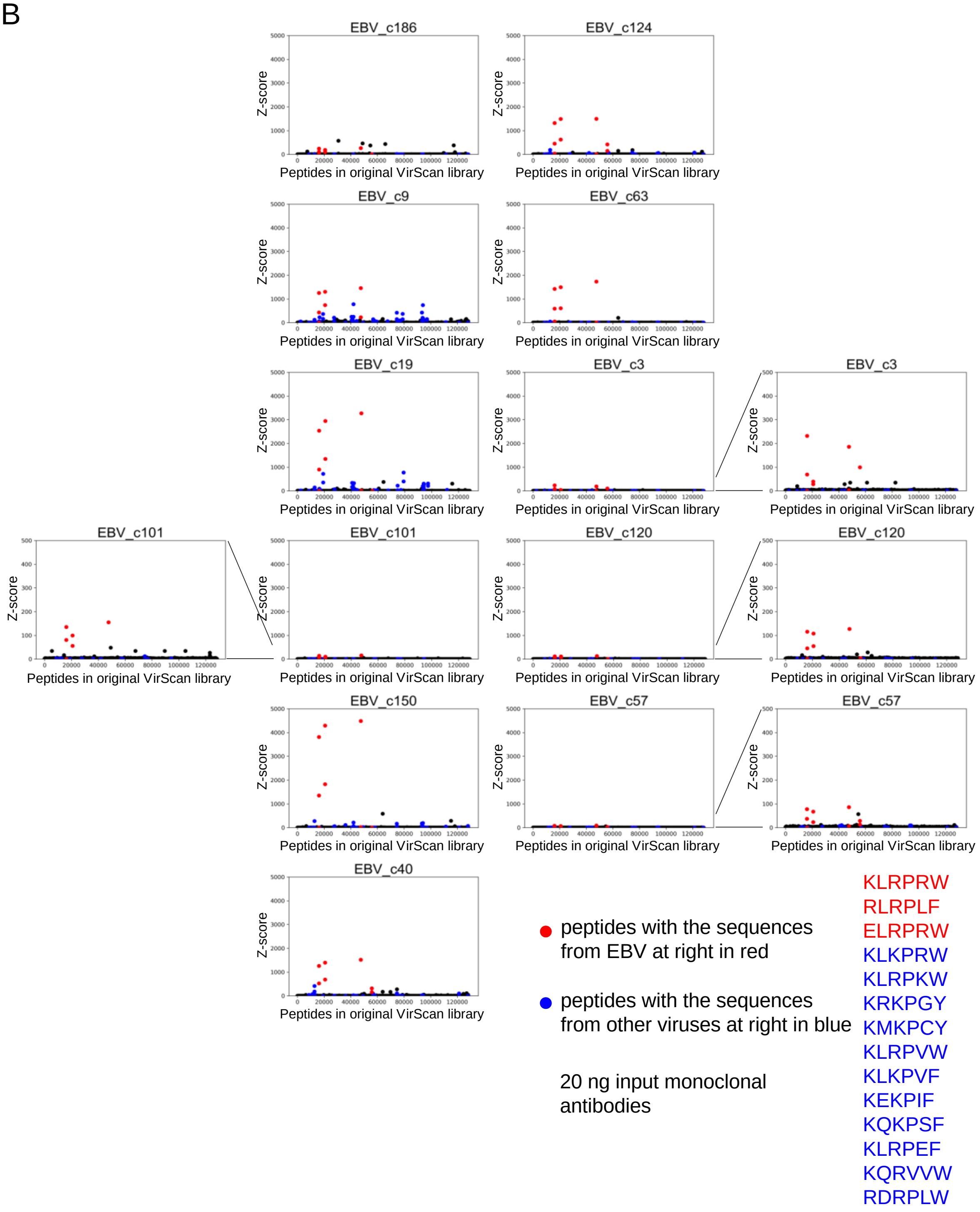

Figure S3.13 continued

C

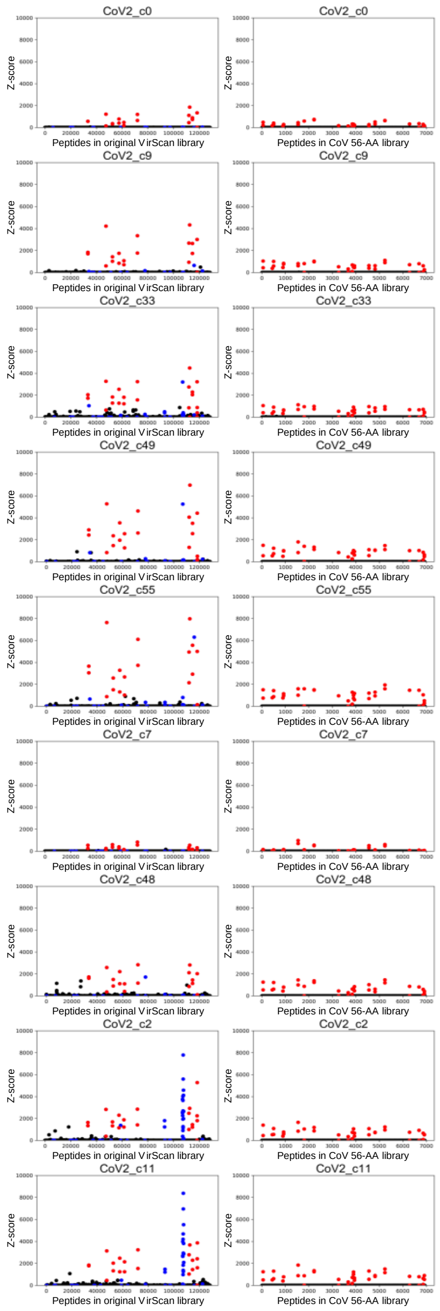

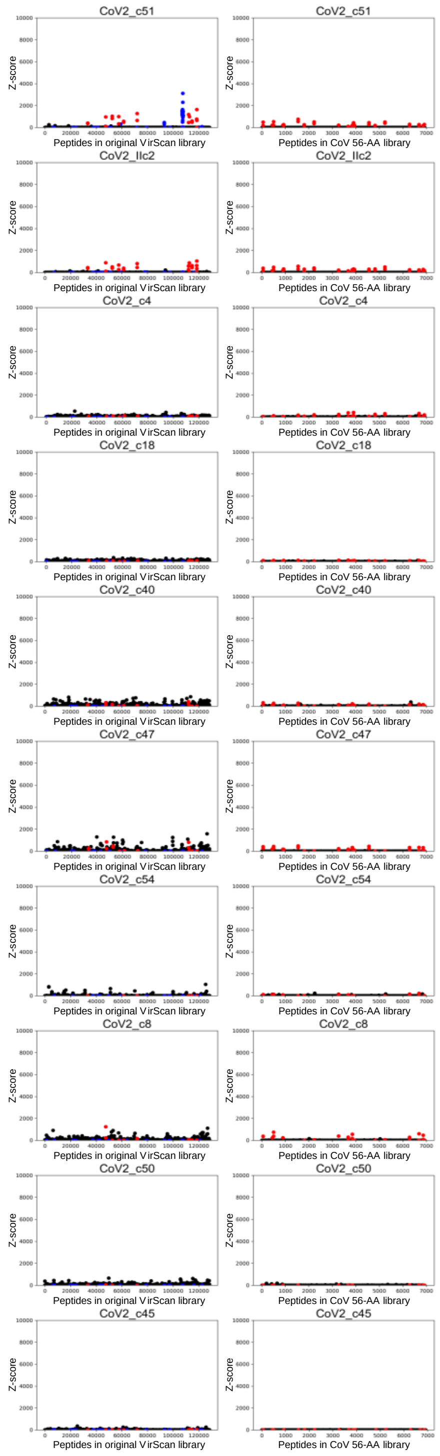

CoV 56-AA library:
56-AA peptides tiling across all proteins from human coronaviruses and closely related bat coronaviruses

● peptides from coronaviruses with the sequences at right in red

● peptides from any other pathogens with the sequences at right in blue

50 μl input cell culture supernatant

EDLLF
EDILF
EELLF
EEAIF
EDCLY
EDKLW
DDCCVF
EDAMY

Figure S3.13. Influenza A-, EBV-, and SARS-CoV-2 minimal peptide-reactive antibodies are not polyspecific. Influenza A- (**A**), EBV- (**B**), and SARS-CoV-2 (**C**) minimal peptide-specific monoclonal antibodies were individually profiled with the human virome VirScan library (3.15, 3.16, 3.18), which contains >100,000 56-AA peptides spanning all proteins from >200 species of human viruses. Additionally, we profiled the SARS-CoV-2 antibodies with the 56-AA human coronavirus supplemental library (3.18). On-target peptides that contain the minimal peptide sequences are shown in red. Cross-reacting peptides with similar sequences to the on-target minimal peptides are shown in blue.

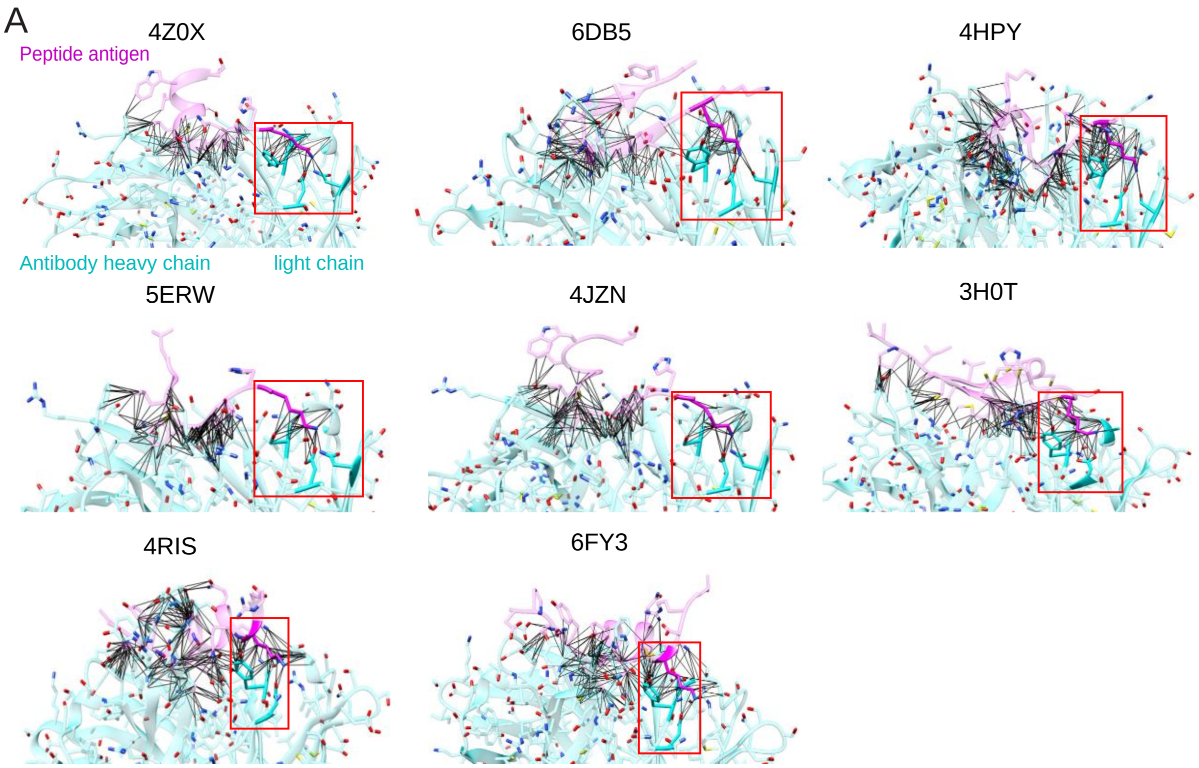
A
4Z0X
6DB5
4HPY
Peptide antigen
Antibody heavy chain
light chain
5ERW
4JZN
3H0T
4RIS
6FY3

Figure S3.14 continued

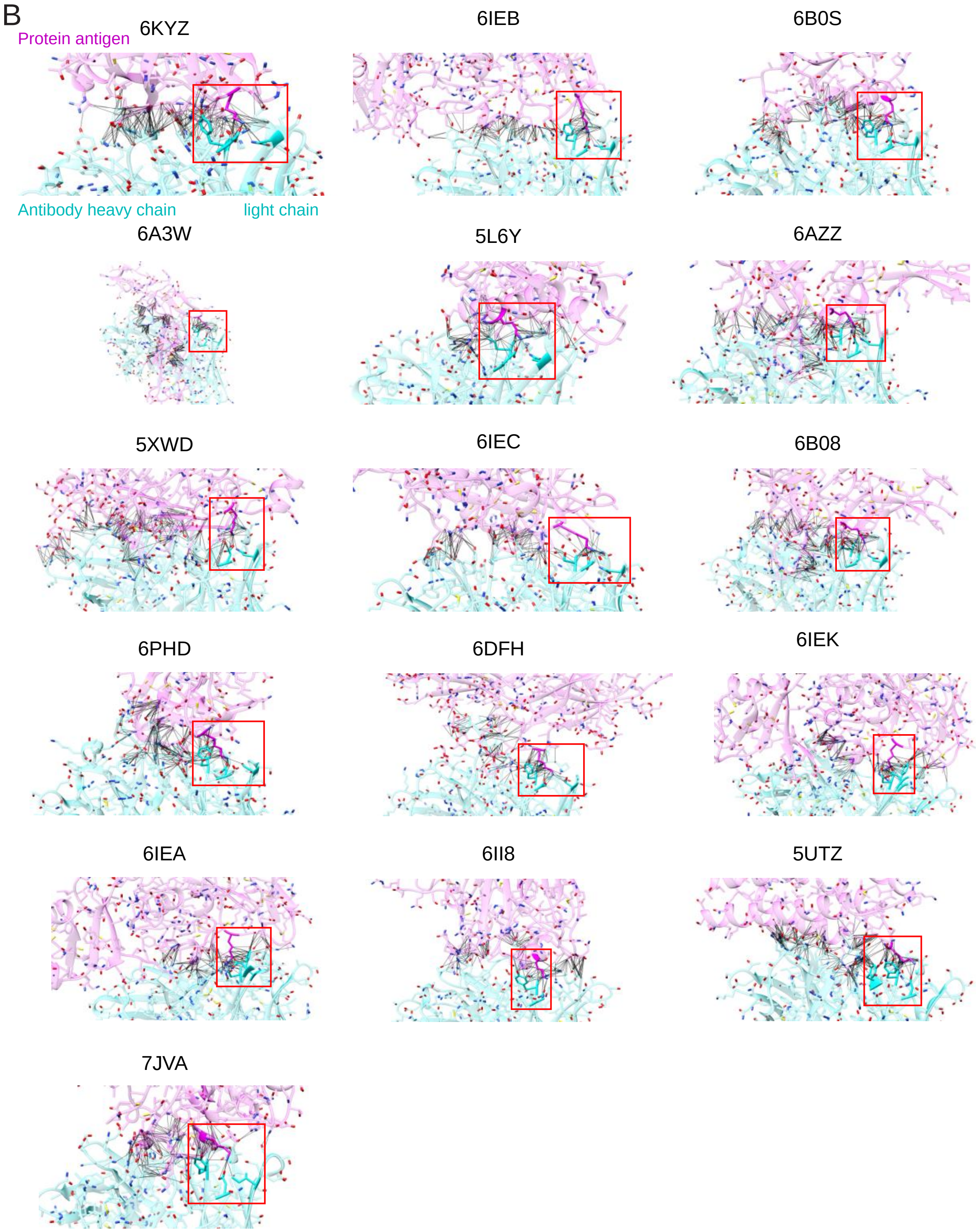

Figure S3.14. The family of human lambda V gene segments with lysine-specific GRAB motifs recognizes border lysines in linear and conformational epitopes. (**A**, **B**) Images of

the epitope-paratope interactions in all unique PDB Ab–Ag structures involving the family of lambda V gene segments described in Fig. 3.5D and table S3.11, tab A, that feature lysine GRAB motif interactions. Ab–Ag structures with peptide antigens are shown in (A); those with protein antigens are shown in (B) (table S3.10). The UCSF Chimera function findclash was used to annotate all contacts between the antibody (colored in translucent cyan) and the antigen (colored in translucent magenta) with black lines. The GRAB motif interactions are shown in red boxes. The GRAB motif is colored in solid cyan and the antigen lysine is colored in solid magenta.

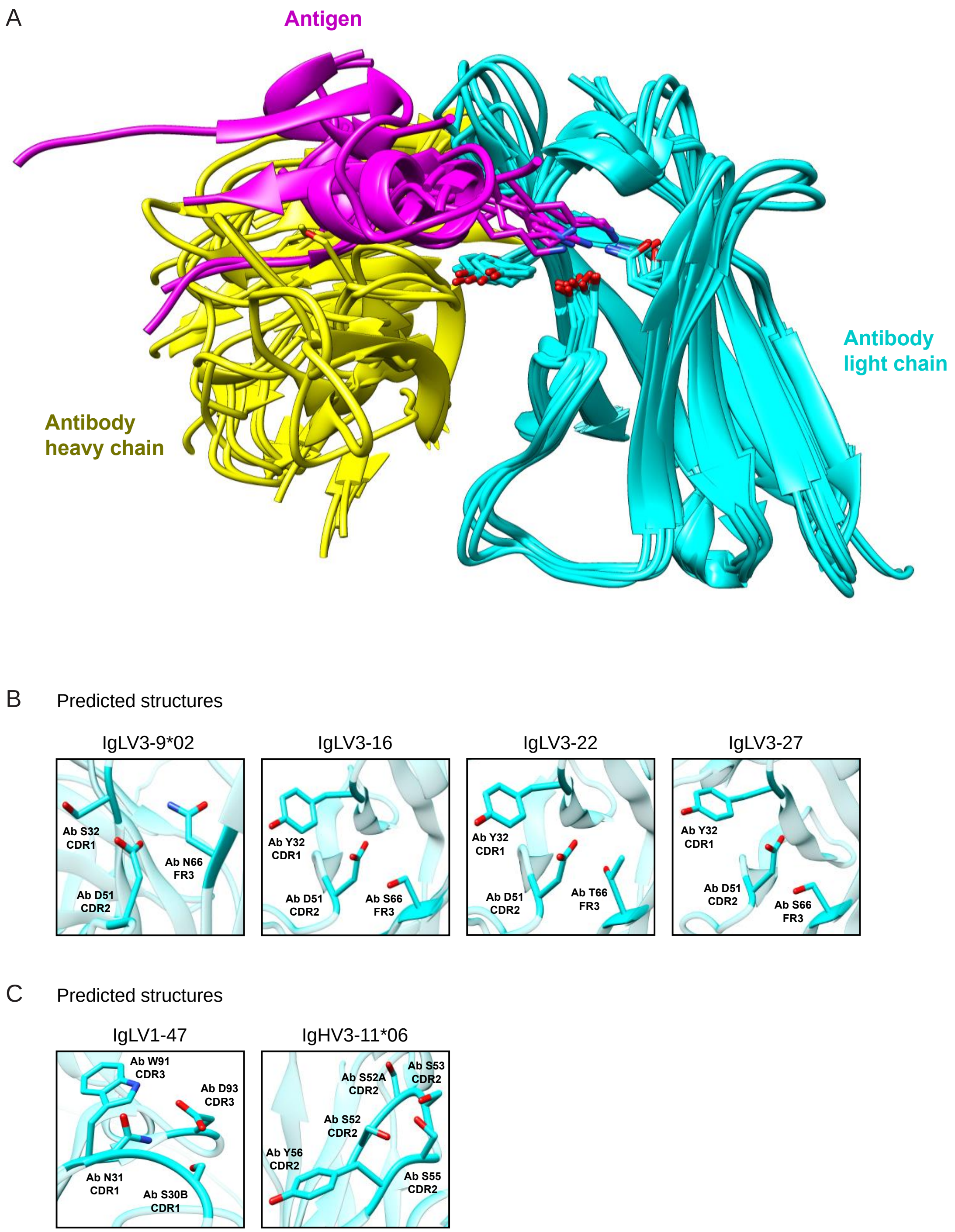

Figure S3.15. GRAB motif interactions are closely aligned in different Ab–Ag structures. (**A**) Structural alignment of all unique PDB Ab–Ag structures involving lambda V gene segments of the family described in Fig. 3.5D and table S3.11, tab A, that feature lysine GRAB motif interactions with peptide antigens (table S3.11A; table S3.10**)**. Alignment was performed in UCSF Chimera (3.72). (**B-C**) Structural predictions of antibodies with V gene segments that share conserved residues with known GRAB motifs. The residues of the hypothetical GRAB motifs are

shown as sticks. Images are labeled as in Fig. 3.5, D and E. Structural predictions were made using AlphaFold2 (3.33, 3.34).

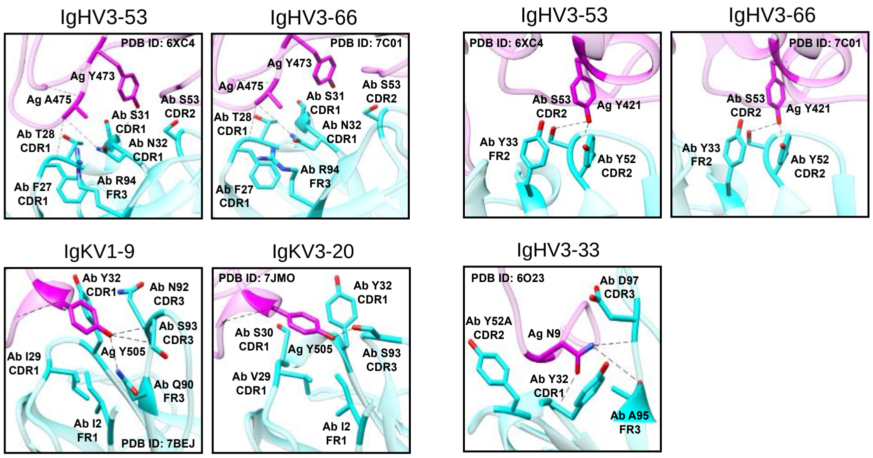

Figure S3.16. Additional recurrent germline-mediated interactions in PDB Ab–Ag structures. (**A**) Representative images of recurrent germline-mediated interactions for the human V gene segments indicated at the top of each image (table S3.10). The images are labeled as in Fig. 3.5, D and E.

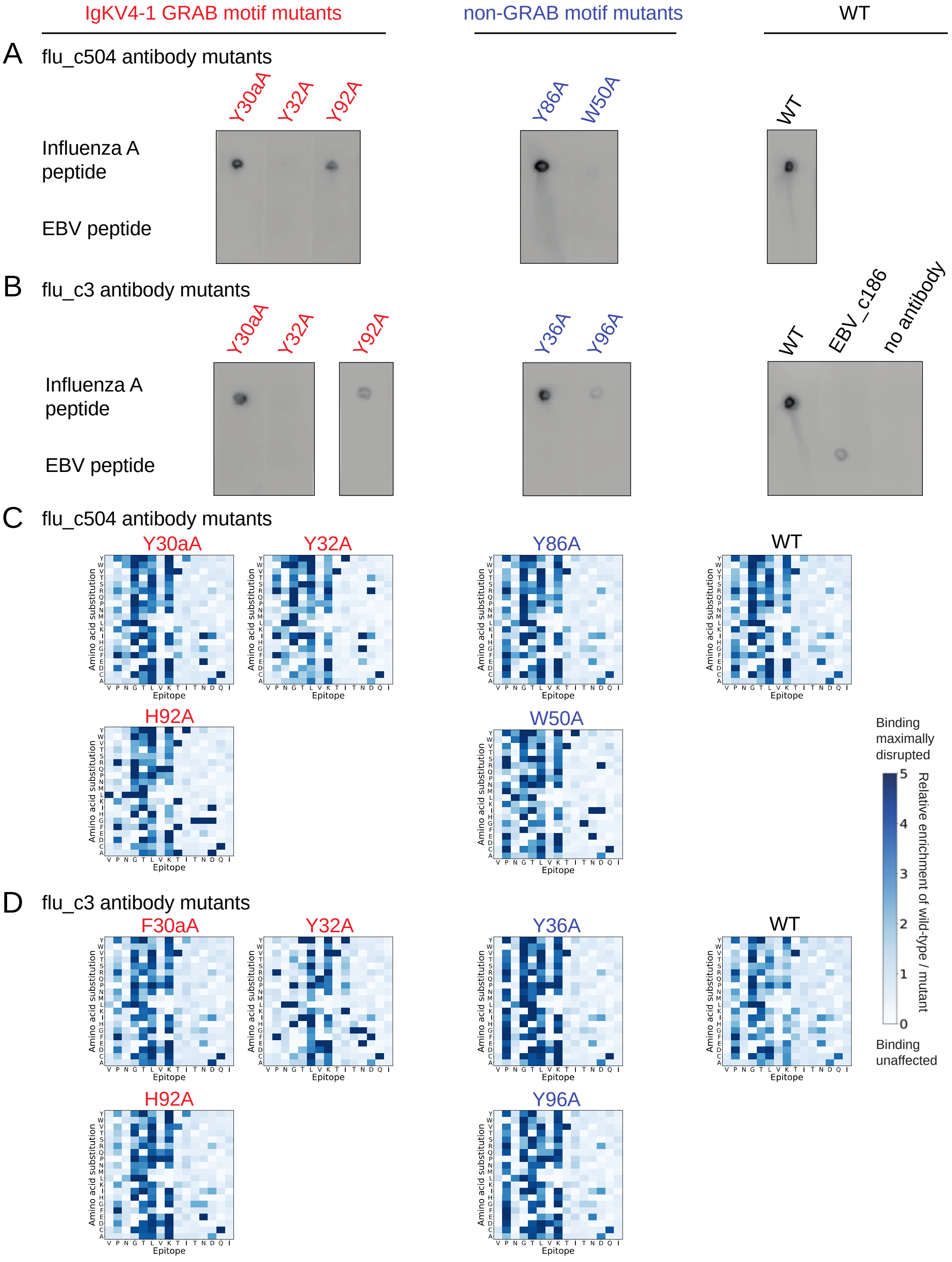

Figure S3.17. IgKV4-1 GRAB motif likely mediates specificity for proline in influenza A public epitope. (**A-B**) Dot blots to assess binding of mutant versions of flu_c504 and flu_c3 to the influenza A minimal peptide. The mutation is listed at the top of each dot blot. Influenza A and EBV minimal peptides were spotted on the nitrocellulose membrane. The EBV minimal peptide

and EBV_c186 antibody were included as controls. (**C-D**) High-resolution footprints for the monoclonal antibodies from panels (A-B). The axes for the high-resolution footprints are as described in Fig. 3.2B.

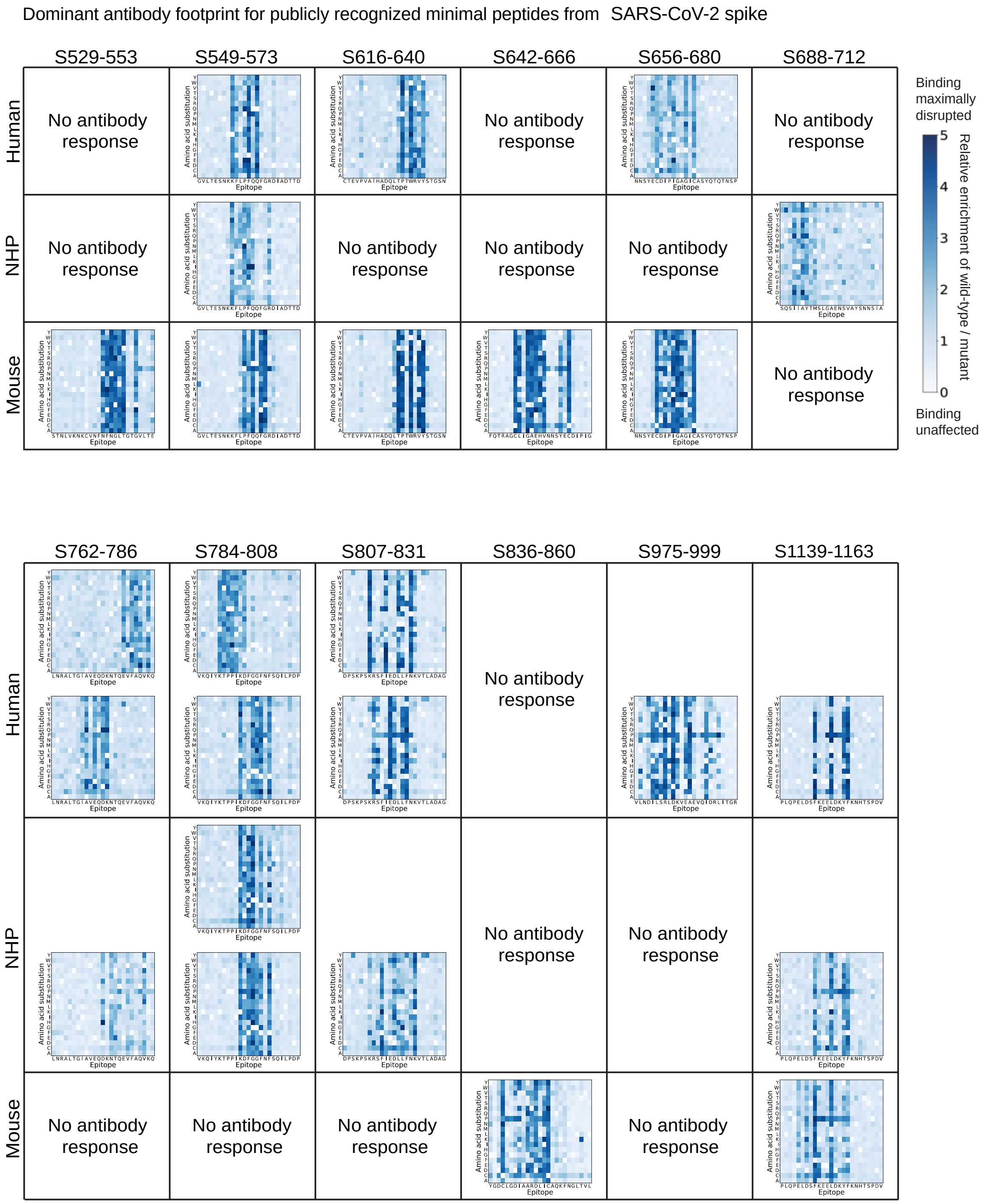

Figure S3.18. SARS-CoV-2 spike public epitopes recognized by humans, NHPs, and mice. Representative high-resolution footprints from human, NHP, and C57BL/6 mouse antibody responses to SARS-CoV-2 public epitopes. The positions of the minimal peptides within spike are labeled at the top of the panel. The heatmaps are labeled as in Fig. 3.2B.

Figure S19

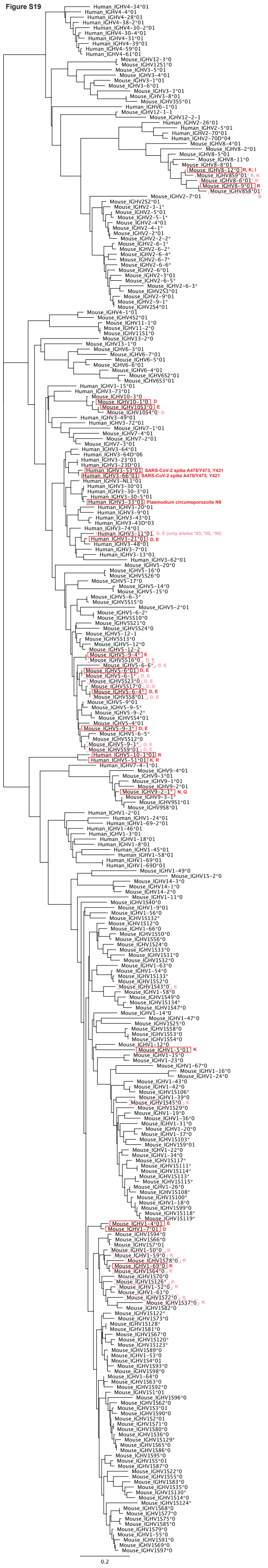

Figure S3.19. Tree of human and mouse IgHV gene segments. Tree depicting the relatedness of human and mouse IgHV gene segments. The AA sequences of the V gene segments were used to create the tree. Only one allele of each human and mouse IgHV gene segment was included for simplicity. The tree was created using Geneious. V gene segments in which we identified GRAB motifs are boxed in red. V gene segments with hypothetical GRAB motifs are boxed in pink dashed lines. The AA specificity of each GRAB motif is indicated to the right of the relevant V gene segment.

Figure S20

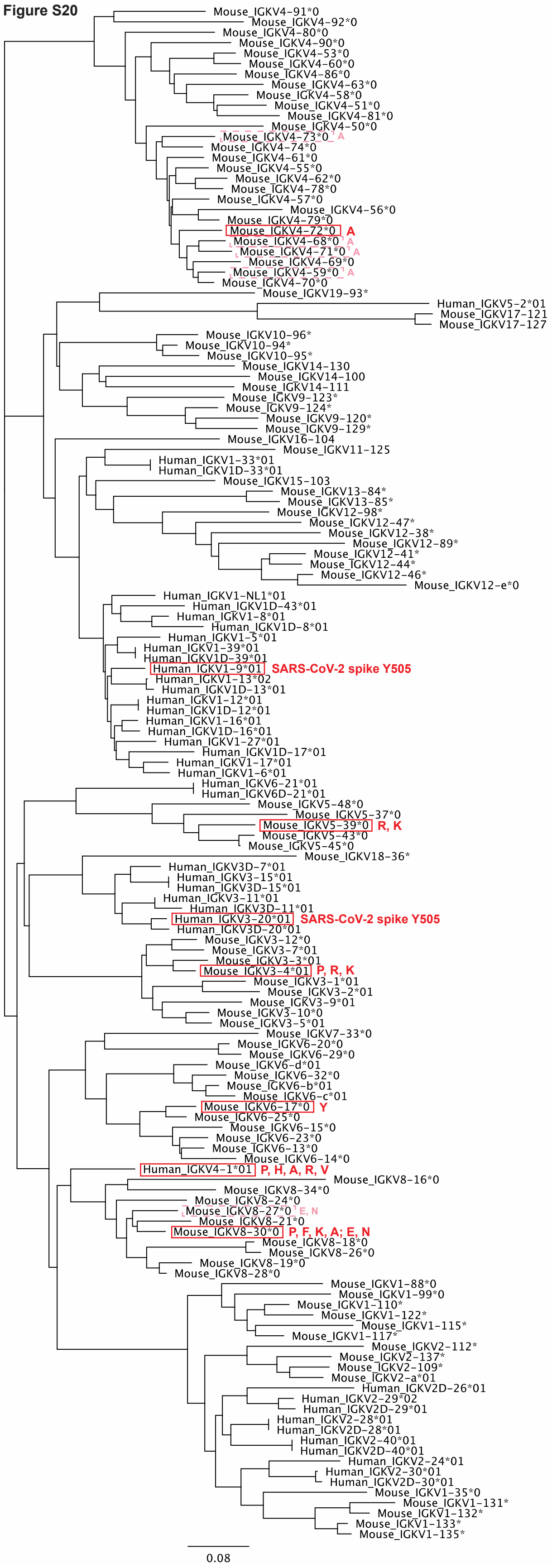
Mouse_IGKV4-91*0
Mouse_IGKV4-92*0
Mouse_IGKV4-80*0
Mouse_IGKV4-90*0
Mouse_IGKV4-53*0
Mouse_IGKV4-60*0
Mouse_IGKV4-86*0
Mouse_IGKV4-63*0
Mouse_IGKV4-58*0
Mouse_IGKV4-51*0
Mouse_IGKV4-81*0
Mouse_IGKV4-50*0
Mouse_IGKV4-73*0 A
Mouse_IGKV4-74*0
Mouse_IGKV4-61*0
Mouse_IGKV4-55*0
Mouse_IGKV4-62*0
Mouse_IGKV4-78*0
Mouse_IGKV4-57*0
Mouse_IGKV4-56*0
Mouse_IGKV4-79*0
Mouse_IGKV4-72*0 A
Mouse_IGKV4-68*0 A
Mouse_IGKV4-71*0 A
Mouse_IGKV4-69*0
Mouse_IGKV4-59*0 A
Mouse_IGKV4-70*0
Mouse_IGKV19-93*
Human_IGKV5-2*01
Mouse_IGKV17-121
Mouse_IGKV17-127
Mouse_IGKV10-96*
Mouse_IGKV10-94*
Mouse_IGKV10-95*
Mouse_IGKV14-130
Mouse_IGKV14-100
Mouse_IGKV14-111
Mouse_IGKV9-123*
Mouse_IGKV9-124*
Mouse_IGKV9-120*
Mouse_IGKV9-129*
Mouse_IGKV16-104
Mouse_IGKV11-125
Human_IGKV1-33*01
Human_IGKV1D-33*01
Mouse_IGKV15-103
Mouse_IGKV13-84*
Mouse_IGKV13-85*
Mouse_IGKV12-98*
Mouse_IGKV12-47*
Mouse_IGKV12-38*
Mouse_IGKV12-89*
Mouse_IGKV12-41*
Mouse_IGKV12-44*
Mouse_IGKV12-46*
Mouse_IGKV12-e*0
Human_IGKV1-NL1*01
Human_IGKV1D-43*01
Human_IGKV1-8*01
Human_IGKV1D-8*01
Human_IGKV1-5*01
Human_IGKV1-39*01
Human_IGKV1D-39*01
Human_IGKV1-9*01 SARS-CoV-2 spike Y505
Human_IGKV1-13*02
Human_IGKV1D-13*01
Human_IGKV1-12*01
Human_IGKV1D-12*01
Human_IGKV1-16*01
Human_IGKV1D-16*01
Human_IGKV1-27*01
Human_IGKV1D-17*01
Human_IGKV1-17*01
Human_IGKV1-6*01
Human_IGKV6-21*01
Human_IGKV6D-21*01
Mouse_IGKV5-48*0
Mouse_IGKV5-37*0
Mouse_IGKV5-39*0 R, K
Mouse_IGKV5-43*0
Mouse_IGKV5-45*0
Mouse_IGKV18-36*
Human_IGKV3D-7*01
Human_IGKV3-15*01
Human_IGKV3D-15*01
Human_IGKV3-11*01
Human_IGKV3D-11*01
Human_IGKV3-20*01 SARS-CoV-2 spike Y505
Human_IGKV3D-20*01
Mouse_IGKV3-12*0
Mouse_IGKV3-7*01
Mouse_IGKV3-3*01
Mouse_IGKV3-4*01 P, R, K
Mouse_IGKV3-1*01
Mouse_IGKV3-2*01
Mouse_IGKV3-9*01
Mouse_IGKV3-10*0
Mouse_IGKV3-5*01
Mouse_IGKV7-33*0
Mouse_IGKV6-20*0
Mouse_IGKV6-29*0
Mouse_IGKV6-d*01
Mouse_IGKV6-32*0
Mouse_IGKV6-b*01
Mouse_IGKV6-c*01
Mouse_IGKV6-17*0 Y
Mouse_IGKV6-25*0
Mouse_IGKV6-15*0
Mouse_IGKV6-23*0
Mouse_IGKV6-13*0
Mouse_IGKV6-14*0
Human_IGKV4-1*01 P, H, A, R, V
Mouse_IGKV8-16*0
Mouse_IGKV8-34*0
Mouse_IGKV8-24*0
Mouse_IGKV8-27*0 E, N
Mouse_IGKV8-21*0
Mouse_IGKV8-30*0 P, F, K, A; E, N
Mouse_IGKV8-18*0
Mouse_IGKV8-26*0
Mouse_IGKV8-19*0
Mouse_IGKV8-28*0
Mouse_IGKV1-88*0
Mouse_IGKV1-99*0
Mouse_IGKV1-110*
Mouse_IGKV1-122*
Mouse_IGKV1-115*
Mouse_IGKV1-117*
Mouse_IGKV2-112*
Mouse_IGKV2-137*
Mouse_IGKV2-109*
Mouse_IGKV2-a*01
Human_IGKV2D-26*01
Human_IGKV2-29*02
Human_IGKV2D-29*01
Human_IGKV2-28*01
Human_IGKV2D-28*01
Human_IGKV2-40*01
Human_IGKV2D-40*01
Human_IGKV2-24*01
Human_IGKV2-30*01
Human_IGKV2D-30*01
Mouse_IGKV1-35*0
Mouse_IGKV1-131*
Mouse_IGKV1-132*
Mouse_IGKV1-133*
Mouse_IGKV1-135*
0.08

Figure S3.20. Tree of human and mouse IgKV gene segments. Tree, created and annotated as in figure S3.19, depicting the relatedness of human and mouse IgKV gene segments.

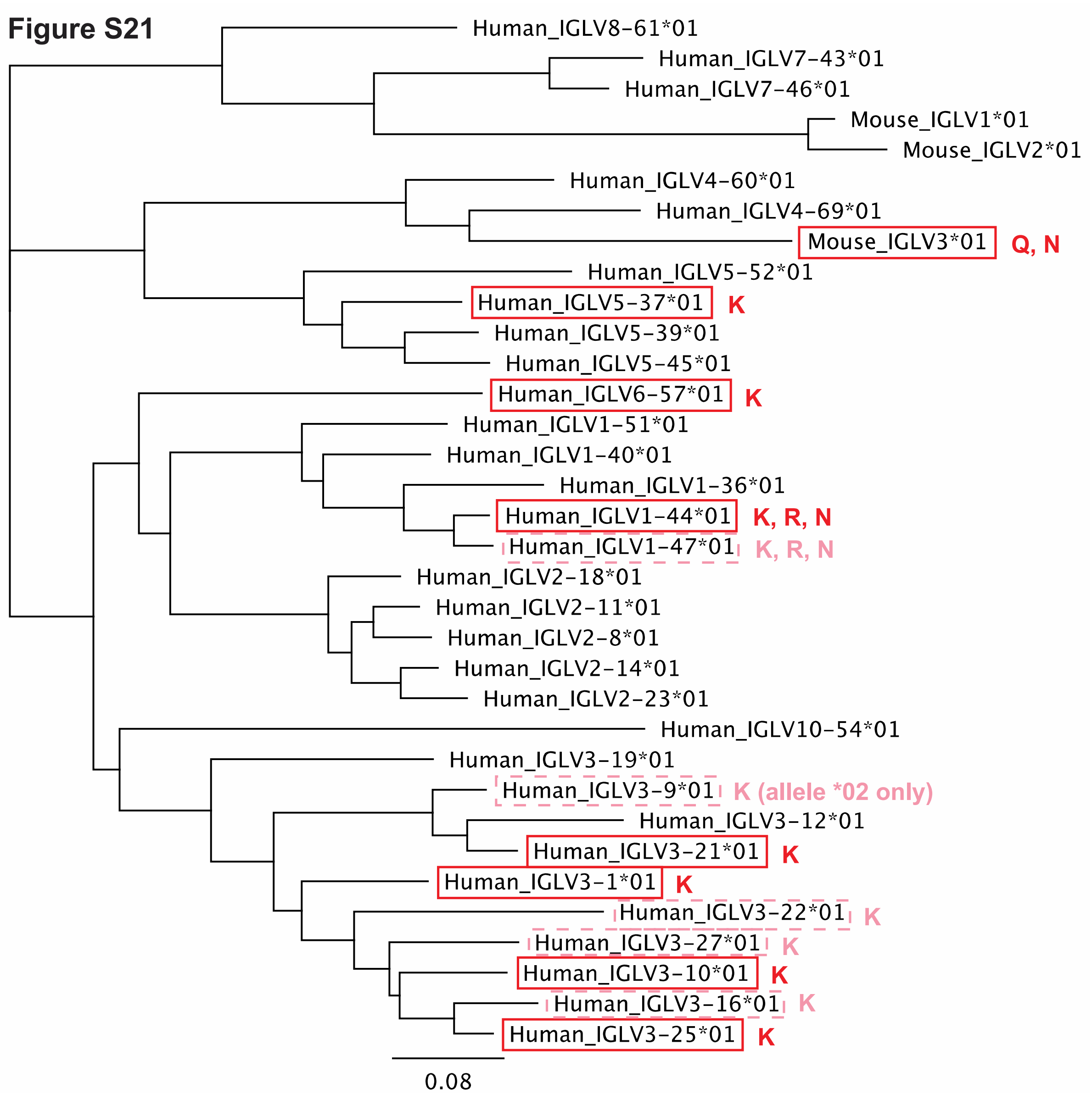

Figure S3.21. Tree of human and mouse IgLV gene segments. Tree, created and annotated as in figure S3.19, depicting the relatedness of human and mouse IgLV gene segments.

Table S3.1. Publicly recognized 56-AA peptides from the original VirScan library. The numbers in the index column refer to the peptide IDs from the human virome VirScan library (3.14-3.16). The column 'fraction_positive' refers to the fraction of individuals seropositive for the relevant viral species who exhibit antibody responses to the given 56-AA peptide.

Table S3.2. Sequences and annotations for the public epitope truncation and alanine scanning library. This file contains concatenated annotations for the human virome VirScan library and the public epitope truncation and alanine scanning library, since VirScan samples were run with these two libraries in combination. Rows with the values "pub_epit_tiling" under column "source" are part of the public epitope truncation and alanine scanning library.

Table S3.3. Minimal public epitope peptide sequences and annotations.

Table S3.4. Sequences and annotations for the saturating mutagenesis public epitope library. The column 'to_synthesize' contains the sequence of the peptide displayed on the phage.

Table S3.5. Consensus critical residues of kappa public epitopes. Tab A. Critical residues are shown in the position of the original peptide. **Tab B.** Critical residues are left-shifted, so the first critical residue is positioned at the first column. Total count of critical residues and span from first to last critical residue are shown at right for each public epitope.

Table S3.6. Consensus critical residues of lambda public epitopes. Tab A. Critical residues are shown in the position of the original peptide. **Tab B.** Critical residues are left-shifted, so the first critical residue is positioned at the first column. Total count of critical residues and span from first to last critical residue are shown at right for each public epitope.

Table S3.7. Counts and proportions of kappa or lambda IP samples that recognize each minimal peptide and that share the consensus critical residues (i.e., are part of the dominant footprint group). The column 'count_total_positive_samples' refers to the number of kappa or, separately, lambda IP samples that recognize the given minimal peptide. Technical replicates were counted separately for this analysis. The column 'count_dominant_footprint_group' refers to the subset of those samples that recognize the consensus critical residues of the kappa or lambda public epitope or only differ by one position.

Table S3.8. Sequence characteristics of all antibodies tested for binding to the influenza A minimal peptide 146786. The column 'validated' refers to whether the antibody was found to bind to the influenza A minimal peptide (peptide 146786 in table S3.3) in validation experiments or not. In cases where a BCR sequence mapped to more than one gene segment with equivalent percent identity, both or all gene segments were listed.

Table S3.9. Sequence characteristics of all antibodies tested for binding to the EBV minimal peptide 72153. The column 'validated' refers to whether the antibody was found to bind to the EBV minimal peptide (peptide 72153 in table S3.3) in validation experiments or not. In cases where a BCR sequence mapped to more than one gene segment with equivalent percent identity, both or all gene segments were listed.

Table S3.10. References for PDB structures.

Table S3.11. Summary of human GRAB motifs. Tab A. GRAB motif interactions observed in PDB Ab–Ag structures for the family of human lambda V gene segments with lysine-specific GRAB motifs. See methods section "PDB analysis to identify GRAB motifs" for column

descriptors**.** Note that the analysis resulting in the 'Interacts_With_Germline_SC' column did not examine CDR3-encoded residues; however, some of the CDR3-encoded residues in the column 'Interacts_With_SC' were germline-encoded and therefore were truly part of the GRAB motif. **Tab B**. Additional human GRAB motif interactions observed in PDB Ab–Ag structures. While mutation of proline was not predicted to destabilize IgKV4-1 Ab–Ag complexes, this could be explained by the fact that the IgKV4-1 GRAB motif can also recognize alanine. **Tab C.** Abbreviated summary of observed and hypothetical human GRAB motifs. **Tab D**. Recurrent germline-encoded interactions observed in anti-SARS-CoV-2 spike antibodies and anti-circumsporozoite protein antibodies.

Table. S3.12. Sequences and annotations for the SARS-CoV-2 public epitope saturating mutagenesis library. This library was developed from a set of minimal SARS-CoV-2 public epitope peptides recognized by humans and mice (3.18).

Table S3.13. Summary of mouse GRAB motifs. Tab A. Mouse GRAB motif interactions observed in PDB Ab–Ag structures, annotated as in table S3.11. Mouse V gene segment sequences were accessed from IMGT (3.49, 3.50) (IMGT Web resources > IMGT Repertoire (IG and TR) > 1. Locus and genes) (https://www.imgt.org/IMGTrepertoire/index.php?section=LocusGenes&repertoire=genetable&species=Mus_musculus&group=IGHV) **Tab B.** Abbreviated summary of observed and hypothetical mouse GRAB motifs.

Table S3.14. Sample legend for public epitope truncation and alanine scanning VirScan dataset.

Table S3.15. Public epitope truncation and alanine scanning VirScan dataset. Each row is a peptide from table S3.2, i.e., the concatenated human virome and public epitope truncation and alanine scanning libraries. Each column is a sample. The values in each cell are the sequencing read counts that correspond to the given peptide in a given sample.

Table S3.16. Sample legend for saturating mutagenesis public epitope VirScan dataset.

Table S3.17. Saturating mutagenesis public epitope VirScan dataset. Each row is a peptide from the saturating mutagenesis public epitope library. Each column is a sample. The values in each cell are the sequencing read counts that correspond to the given peptide in a sample.
